# Longitudinal monitoring of disease burden and response using ctDNA from dried blood spots in xenograft models

**DOI:** 10.15252/emmm.202215729

**Published:** 2022-06-13

**Authors:** Carolin M Sauer, Katrin Heider, Jelena Belic, Samantha E Boyle, James A Hall, Dominique‐Laurent Couturier, Angela An, Aadhitthya Vijayaraghavan, Marika AV Reinius, Karen Hosking, Maria Vias, Nitzan Rosenfeld, James D Brenton

**Affiliations:** ^1^ Cancer Research UK Cambridge Institute University of Cambridge Cambridge UK; ^2^ Cancer Research UK Major Centre–Cambridge University of Cambridge Cambridge UK; ^3^ Medical Research Council Biostatistics Unit University of Cambridge Cambridge UK; ^4^ Cambridge University Hospitals NHS Foundation Trust and National Institute for Health Research Cambridge Biomedical Research Centre Addenbrooke's Hospital Cambridge UK; ^5^ Department of Oncology University of Cambridge Cambridge UK

**Keywords:** circulating tumour DNA, copy number aberrations, liquid biopsies, PDX models, preclinical treatment study, Cancer

## Abstract

Whole‐genome sequencing (WGS) of circulating tumour DNA (ctDNA) is now a clinically important biomarker for predicting therapy response, disease burden and disease progression. However, the translation of ctDNA monitoring into vital preclinical PDX models has not been possible owing to low circulating blood volumes in small rodents. Here, we describe the longitudinal detection and monitoring of ctDNA from minute volumes of blood in PDX mice. We developed a xenograft Tumour Fraction (xTF) metric using shallow WGS of dried blood spots (DBS), and demonstrate its application to quantify disease burden, monitor treatment response and predict disease outcome in a preclinical study of PDX mice. Further, we show how our DBS‐based ctDNA assay can be used to detect gene‐specific copy number changes and examine the copy number landscape over time. Use of sequential DBS ctDNA assays could transform future trial designs in both mice and patients by enabling increased sampling and molecular monitoring.

## Graphical abstract

A novel approach is developed for longitudinal monitoring of tumour burden in patient‐derived xenograft (PDX) models using dried blood spots from minute volumes of blood.

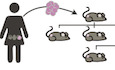

**The paper explained**

**Problem**

Whole‐genome sequencing (WGS) of circulating tumour DNA (ctDNA) has enabled non‐invasive disease stratification and monitoring of disease progression and response in the clinic. Patient‐derived xenograft (PDX) mice are frequently used as models to study new treatment approaches for human cancers. However, WGS‐based ctDNA assays have not been possible in small rodents owing to constraints on the volume of blood that can be sampled.

**Result**

We developed shallow WGS (sWGS) of ctDNA from serial and minimally invasive dried blood spot (DBS) samples. We show that copy number changes are detected over multiple time points and DBS ctDNA recapitulates the biological features of ctDNA in patients. Sequential DBS ctDNA accurately predicts treatment response and disease outcome in PDX mouse models.

**Impact**

Our approach enables minimally invasive sampling and sWGS‐based detection of ctDNA over time from minute volumes of whole blood (~ 50 μl) in preclinical animal models. It strongly conforms with the 3Rs of animal welfare and has the potential to revolutionise study design in both small rodents and patients.

## Introduction

Liquid biopsies are routinely used in the clinic to sensitively detect and quantify disease burden, and have critical roles for therapeutic decision making in precision medicine (Wan *et al*, 2017; Cohen *et al*, 2018; Heitzer *et al*, 2019; Rothwell *et al*, 2019; Kilgour *et al*, 2020; Deveson *et al*, 2021). Plasma circulating tumour DNA (ctDNA) is the most widely studied circulating analyte for disease monitoring and molecular genotyping of tumours (Cescon *et al*, 2020; Kilgour *et al*, 2020). Technical advances in next generation sequencing (NGS) now achieve unprecedented sensitivities for the detection of ctDNA using 6–10 ml of whole blood (Deveson *et al*, 2021; Rolfo *et al*, 2021). To enable very accurate monitoring of disease burden and progression, several whole‐genome sequencing (WGS)‐based strategies have been developed detecting combinations of single‐nucleotide variants, small insertions/deletions and somatic copy number aberrations (SCNAs) (Adalsteinsson *et al*, 2017; Chen & Zhao, 2019; Wan *et al*, 2020; Zviran *et al*, 2020; Abbosh & Swanton, 2021; Paracchini *et al*, 2021). In addition, deriving other biochemical features of ctDNA from WGS, including fragment size and chromosome accessibility, can further enhance detection sensitivity and infer biological information about tumour site of origin (Mouliere *et al*, 2018; Cristiano *et al*, 2019; Ulz *et al*, 2019; Keller *et al*, 2021; preprint: Markus *et al*, 2021).

Modelling therapeutic response in mice bearing patient‐derived xenografts (PDX) is a critical step to test treatment regimens and pharmacogenomics during drug development (Williams, 2018; Ireson *et al*, 2019). However, WGS‐based ctDNA assays cannot be used in small rodents as the circulating blood volume of a mouse is only ~ 1.5–2.5 ml. Consequently, detailed ctDNA assays can only be obtained from terminal bleeding of mice, preventing longitudinal analyses and more efficient therapeutic study designs. Manual measurements of tumour volumes in subcutaneous models are the commonest surrogate to estimate treatment response and disease burden (Pearson *et al*, 2016; Ice *et al*, 2019). These measures are often poorly reproducible and can be biased by treatment‐induced tissue necrosis and oedema. Using imaging as an alternative to estimate response in PDXs is more time‐consuming, requires general anaesthesia and may also need the introduction of *in vivo* reporter genes (Weissleder, 2002; Koessinger *et al*, 2020).

Therefore, bringing WGS‐based ctDNA assays into mice would have two major benefits. Firstly, more efficient and accurate serial measurements across multiple animals, and secondly, the direct translation of biological and biochemical observations from mouse ctDNA studies into patient studies and vice versa. We recently illustrated the detection of ctDNA in dried blood spots (DBS) from minute volumes of whole blood using a size selection approach to enrich for cell‐free DNA (cfDNA) (Heider *et al*, 2020b). Using a modified approach in PDX mice, we now demonstrate that shallow WGS (sWGS) of DBS from 50 µl of whole blood can be used for serial ctDNA measurements, longitudinal disease monitoring and copy number analyses in preclinical studies. The work presented here provides important proof‐of‐principle data and further supports the application and feasibility of DBS‐based ctDNA sampling both in preclinical and clinical studies.

## Results

### Development and validation of the xTF metric from DBS

**Figure 1:**
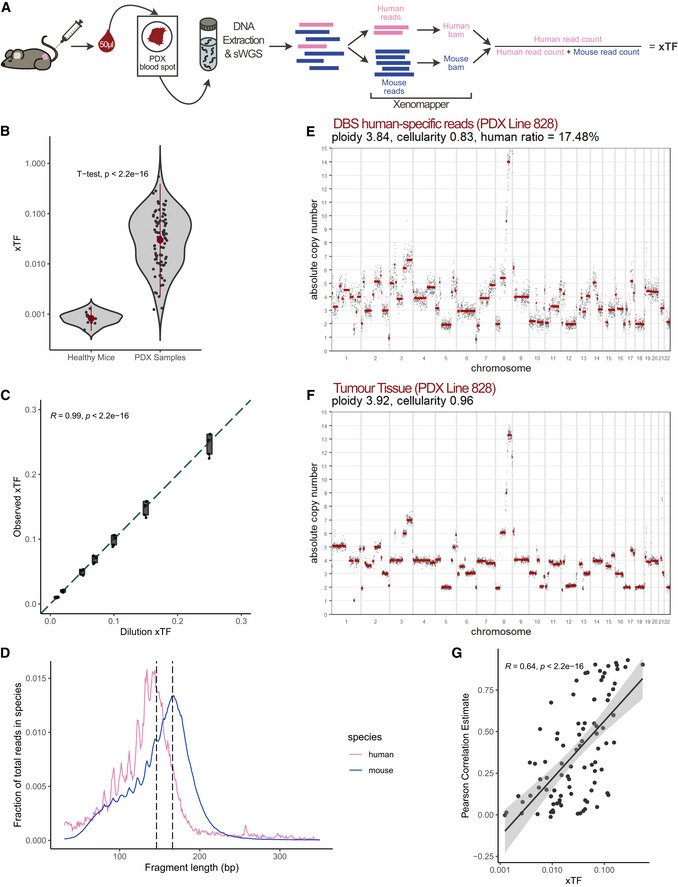
The xTF metric is highly specific and sensitive to detect and quantify ctDNA from dried blood spots Workflow of the dried blood spot (DBS)‐based xenograft Tumour Fraction (xTF). DBS are generated by collecting and depositing 50 µl of blood from the tail vein of the mouse onto FTA filter cards. DNA is extracted from blood spots, processed and sequenced as described previously (Heider *et al*, 2020b). Human‐specific reads and mouse‐specific reads were separated into species‐specific bam files using Xenomapper (Wakefield, 2016). The xTF is then calculated by dividing the number of human‐specific reads by the total number of human and mouse‐specific reads in a given sample.Comparison of xTF values obtained from healthy non‐tumour‐bearing mice DBS (*n* = 10, from 5 individual mice) and PDX DBS (*n* = 91, from 35 individual mice at day 1, 16 or 29) samples (Welch *t*‐test, *P* < 2.2 × 10^−16^). Sensitivity testing using the Mann–Whitney *U* Wilcoxon test (Wilcoxon test, *P* = 2.5 × 10^−7^) showed similar results. Mean ± SD are indicated in red.xTF dilution series. Dilution xTFs (0.01, 0.02, 0.05, 0.07, 0.1, 0.15 and 0.25) were computationally generated by mixing blood spot sequencing data obtained from five ovarian cancer patients and a healthy control mouse. Each dilution therefore contains five biological replicates. The generated dilution series was analysed using Xenomapper and resulting xTF values were compared with the dilution xTFs (Spearman correlation *R* = 0.99, *P* < 2.2 × 10^−16^). Boxplots indicate first quartiles, medians (vertical line) and third quartiles. Whiskers indicate minima and maxima.Fragment length distributions of human‐ (pink) and mouse‐ (blue) specific reads from a DBS sample. Two vertical lines indicate 146 and 166 bp, the observed peaks for ctDNA and cfDNA, respectively.Example of an absolute copy number (ACN) profile successfully generated from human‐specific reads from a DBS collected from a PDX mouse of patient line 828.Matching ACN profile generated from sWGS of PDX tumour tissue. Appendix Figs S2 and S3 show representative ACN profiles for all four patient lines and the correlation of each copy number bin for the DBS and tissue sample pairs.Correlation of Pearson correlation estimates (comparing ACN bins between tumour tissue and DBS) and xTFs from DBS samples (Spearman *R* = 0.64, *P* < 2.2 × 10^−16^).

To detect and accurately quantify ctDNA from minute volumes of blood in preclinical PDX studies, we developed a xenograft Tumour Fraction (xTF) metric, which is estimated from shallow whole‐genome sequencing (sWGS) of DBS samples (Fig 1A). Briefly, 50 µl of blood is collected from the tail vein, deposited onto a filter card, and left to air dry. DNA is extracted, contaminating genomic DNA is removed (Heider *et al*, 2020b) and subsequently sequenced at low coverage following library preparation. Human‐ and mouse‐specific reads are identified using Xenomapper (Wakefield, 2016), and the xTF is calculated as the ratio of human‐specific reads divided by total reads (human and mouse‐specific reads) per sample (see Methods).

To test both the specificity and sensitivity of the xTF metric, we established a preclinical study using PDX mice derived from four high‐grade serous ovarian cancer (HGSOC) patients (see next section). We collected a total of 10 DBS samples from five healthy non‐tumour‐bearing mice and 91 DBS samples from 35 tumour‐bearing PDX mice. Reads from healthy control mice showed < 0.1% assignment as human‐specific sequences (false‐positive background). In addition, healthy control mice had significantly lower xTF values compared to tumour‐bearing PDX mice, independent of tumour size and disease burden, indicating the high specificity of the xTF metric (Welch *t*‐test, *P* = 2.2 × 10^−16^, Fig 1B). To confirm the linearity and sensitivity of our approach, we prepared an *in silico* 7‐point dilution series (see Methods) by combining sequencing reads from a healthy mouse DBS and DBS samples collected from five independent ovarian cancer patients at different ratios. We were able to accurately detect human reads for all seven dilution points, and observed a strong correlation between measured xTFs and spiked‐in human reads at human:mouse proportions of 1–25% (Spearman’s *R* = 0.99, *P* < 2.2 × 10^−16^, Fig 1C).

Next, we examined the fragment size distributions of human‐ and mouse‐specific reads from sWGS of DBS samples. In human plasma samples, ctDNA has a modal size of approximately 145 bp, which is shorter than cfDNA with a prominent mode of approximately 165 bp (Jahr *et al*, 2001; Underhill *et al*, 2016; Mouliere *et al*, 2018). These fragment size properties were recapitulated in the human‐ and mouse‐specific reads from DBS samples (Fig 1D). By contrast, human‐specific reads incorrectly identified in non‐tumour‐bearing control mice (false‐positive background; see Fig 1B) displayed significantly smaller fragment sizes, with the majority of fragment sizes < 50 bp (Appendix Fig 1) suggesting non‐specific alignment.

Given the high specificity and sensitivity of our approach, we were able to derive absolute copy number (ACN) data from as little as 500,000 human‐specific DBS reads using QDNAseq (Scheinin *et al*, 2014) followed by Rascal (Sauer *et al*, 2021) (Fig 1E). The observed absolute somatic copy number aberrations (SCNAs) (Fig 1F) and their extent were strongly correlated with sWGS of PDX tumour tissues from the same patient (Appendix Figs S2 and S3A–D). Unsurprisingly, the ability to accurately detect SCNAs in DBS strongly correlated with increasing xTF values (Fig 1G). No correlations were observed when comparing blood spot ACN profiles from healthy non‐tumour‐bearing mice to any of the four patient tumour tissues (Appendix Fig S3E–G). Using the definitions of copy number gains and losses outlined by the Catalogue of Somatic Mutations In Cancer (COSMIC), amplifications of driver SCNAs were detectable in blood spot samples with xTFs ranging from 0.6–54.4% (Appendix Figs S2 and S3H).

### The xTF allows accurate monitoring of disease progression

**Figure 2:**
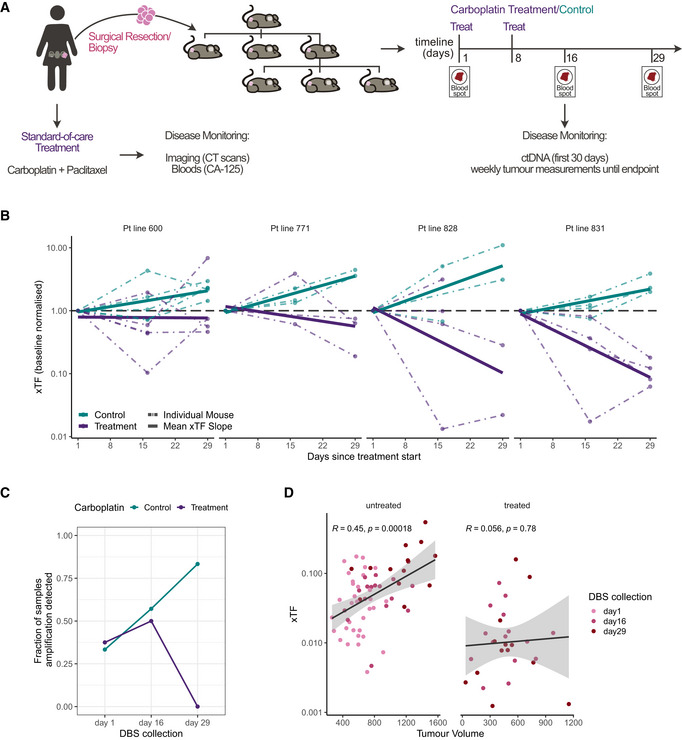
The DBS‐based xTF allows longitudinal monitoring of disease progression and treatment response in preclinical studies Preclinical PDX study overview. HGSOC patients underwent surgery and standard‐of‐care chemotherapy with carboplatin and paclitaxel. Disease progression was monitored over time using the CA‐125 biomarker, CT scans, as well as ctDNA where available. The treatment‐naïve surgical tumour or biopsy specimens were engrafted into NSG mice. Second or third generation PDX mice were then treated with either carboplatin or vehicle control via tail vein injection on day 1 and day 8. Tumour volumes were measured weekly, and blood spots were collected on day 1 (prior to treatment initiation), day 16 and 29.xTF change from baseline during the first 29 days since start of treatment for each PDX patient line. xTFs were normalised to baseline (day 1) xTF values for each mouse (dashed lines). Carboplatin‐treated mice are shown in purple, control mice are shown in teal. Bold lines show the linear‐model fitted across all mice within the same treatment and patient group. Horizontal dashed lines at *y* = 1 indicate normalised baseline.Fraction of blood spot samples in which putative driver amplifications were detected over time. The fraction of samples with detected gene amplifications decreases in the carboplatin‐treated group, while increasing in the control group over time.Correlation between xTF values and tumour volumes of the nearest matched time point for both untreated (Pearson’s *R* = 0.45, *P* = 0.0002), and carboplatin‐treated (Pearson’s *R* = 0.056, *P* = 0.78) PDX mice.
Source data are available online for this figure.

**Figure EV1:**
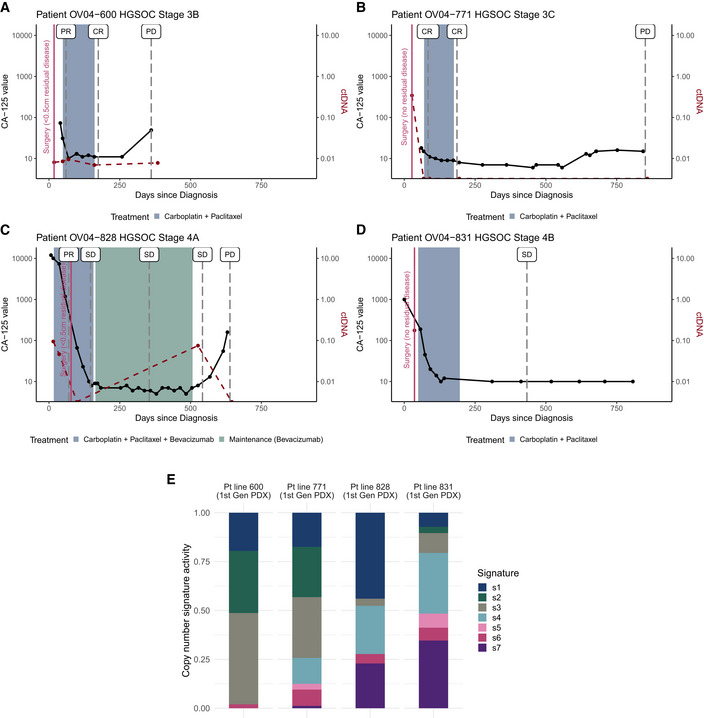
Clinical treatment response, surgery outcome, time until progression and copy number signatures for preclinical study patients A–DCA‐125 values (black line), treatment response assessments estimated via CT scans (vertical grey dashed lines), and ctDNA data, where available (red dashed line), for HGSOC patients 600, 771, 828 and 831, respectively, over time. Surgery and additional treatment regimens are indicated by a pink vertical line and shaded boxes, respectively (CR, Complete Response; PR, Partial Response; SD, Stable Disease; PD, Progressive Disease).EStacked bar plots showing copy number signature activities for first generation PDX tissues derived from the four patients (patient 600, 771, 828 and 831) used in the preclinical HGSOC study.

We next investigated whether the DBS‐based xTF assay could be used for longitudinal monitoring of disease progression and treatment response. An overview of our preclinical PDX study is shown in Fig 2A. The PDX models were selected from four patients with different clinical responses to platinum‐based chemotherapy and distinct copy number signatures (Macintyre *et al*, 2018) for homologous recombination deficiency (HRD) that are predictive of sensitivity to carboplatin (Fig EV1). All PDXs were derived from tumour samples prior to systemic therapy and histological and molecular features were shown to be highly similar to the primary tumour (Appendix Figs S4 and S5). PDX mice were treated with either 50 mg/kg carboplatin or control on day 1 and 8. Tumour volumes were measured weekly, and blood spots were collected on day 1 (prior to treatment start), day 16 and 29 (Fig 2A).

We observed a progressive increase in xTF in all 17 untreated PDX control mice. In contrast, the 18 mice that were treated with carboplatin showed PDX‐specific decreases in xTFs from DBS samples collected at day 16 and 29 in comparison to pretreatment (day 1) samples (Fig 2B). Similarly, the fraction of samples in which we were able to detect human gene‐level amplifications from DBS reads (e.g. *MYC* and *MCM10* amplifications in patients 828 and 771, respectively) increased in untreated and decreased in carboplatin‐treated mice over time (Fig 2C). When correlating xTF values to tumour volumes obtained from weekly tumour measurements, we found that xTFs increased with increasing tumour volumes and thus disease burden (Pearson’s *R* = 0.48, *P* = 1.2 × 10^−6^, Appendix Fig S6A). This correlation was strongly observed in all untreated mice (Pearson’s *R* = 0.45, *P* = 0.0002, Fig 2D), but not in all treated mice (Pearson’s *R* = 0.056, *P* = 0.78, Fig 2D), mostly related to responses in PDX mice from patient line 831 (Appendix Fig S6B). This could be due to treatment‐induced tissue necrosis and oedema biasing manual tumour volume measures, and suggests that ctDNA measures could offer a more accurate readout of initial treatment response (during the first 30 days) as less prone to confounding factors on manual size measurements.

### The xTF rate of change is predictive of disease outcome

**Figure EV2:**
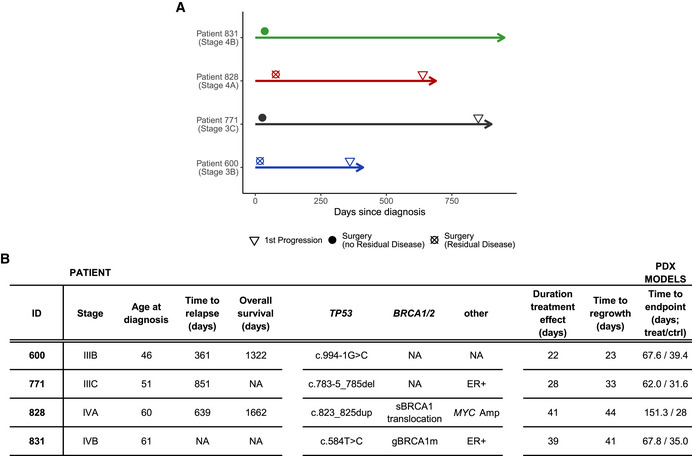
Overview of preclinical patients and PDX lines Overview of clinical timelines until first disease progression in the four HGSOC patients.Table summarising clinical, genomic and PDX tumour growth kinetics information for all four patients included in this study. Patient 600, 771 and 831 underwent primary surgery prior to treatment, whereas patient 828 received neoadjuvant treatment prior to interval debulking surgery and further treatment. Both patient 600 and 828 had residual disease following surgery.

Early dynamic change in ctDNA can predict progression‐free survival and provide real‐time assessment of treatment efficacy (O’Leary *et al*, 2018). Similar predictive measures in mice could also improve the efficiency of PDX study designs. All four PDX lines in our cohort were from patients with platinum‐sensitive disease, and PDX 828 and 831 were predicted to have the best response to carboplatin treatment owing to somatic and germline *BRCA1* mutations, respectively (Figs EV1 and EV2). PDX 600 and 771 had less marked HRD signatures (Figs EV1E and EV2). Clinical progression‐free survival (PFS) and overall survival (OS) (Fig EV2) could not be used as response predictors as the four patients have important differences in prognostic variables for stage and residual disease after surgery (Fig EV1).

**Figure 3:**
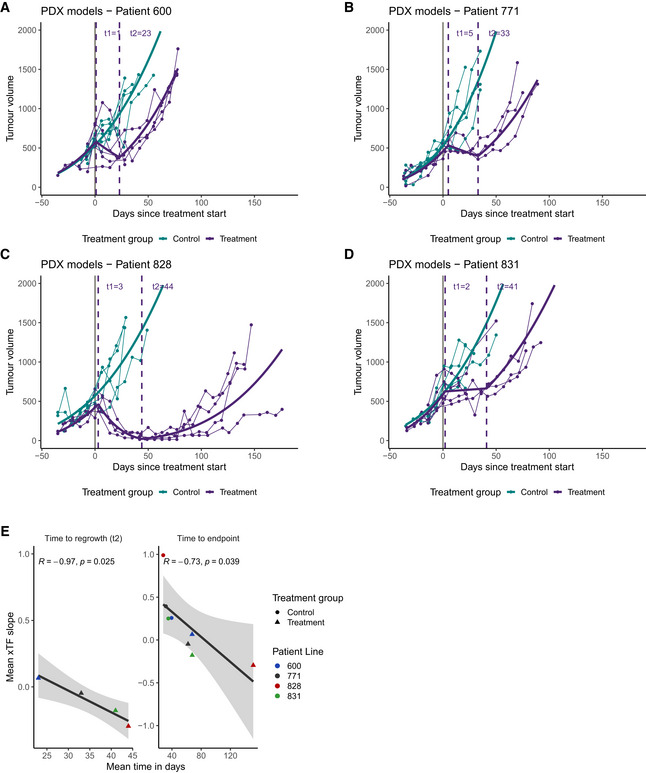
Change in xTF over time predicts disease outcome A–DWeekly measured tumour volumes (mm^3^) for PDX mice over time. Treatment start is indicated by solid grey vertical line. Solid coloured lines show modelled tumour growth curves using heteroscedastic point‐wise random intercept linear mixed models (see Methods). Growth curve inflection points were determined (see Methods) to estimate the start of treatment effect and tumour regrowth (dashed vertical purple lines labelled *t*_1_ and *t*_2_, respectively).EThe mean xTF slope was estimated for each treatment group across the four patient lines (Fig 2B) and compared with the mean time to endpoint (Pearson’s *R* = −0.73, *P* = 0.039) and tumour regrowth (Pearson’s *R* = −0.97, *P* = 0.025). The four different patient lines are indicated by different colours.
Source data are available online for this figure.

We asked whether the rate of change in xTFs during the first 30 days following initiation of treatment was predictive of disease outcome in our PDX cohort. Given the poor correlation between xTFs and tumour volumes (Fig 2D), we explored tumour growth kinetics from weekly tumour measurements taken from the time of tumour engraftment until study endpoint for carboplatin‐treated and untreated mice (see Methods, Fig 3A–D). Tumour volumes and growth rates were not significantly different between treatment and control mice across the four lines prior to start of treatment (Appendix Table S1). Importantly, the rates of tumour regrowth in treated mice were not significantly different from initial growth rates after engrafting and prior to treatment start (Appendix Table S1), providing evidence that carboplatin treatment (and potential clonal selection) did not change tumour growth kinetics. We then inferred inflection points representing treatment‐induced changes in tumour growth rates, allowing estimation of both the time of treatment response (t_1_) and time of tumour regrowth (t_2_) (Fig 3A–D). t_2_−t_1_ therefore represents the duration of treatment effect, and t_2_ is comparable with PFS, the commonest clinically validated surrogate endpoint for clinical trials. As predicted, t_2_−t_1_ measures were longest (representing best response) for PDX 828 and 831 and the worst response was seen in PDX 600.

Importantly, there was a strong negative correlation between the xTF change rate (see Fig 2B) during the first 30 days of treatment and t_2_ (tumour regrowth; Pearson’s *R* = −0.97, *P* = 0.025; Fig 3E). The xTF change rate was also strongly correlated with study endpoint (a surrogate for overall survival; Pearson’s *R* = −0.73, *P* = 0.039; Fig 3E).

## Discussion

We demonstrate for the first time that minimally invasive sampling of DBS can be used to accurately monitor disease progression and treatment response in PDX mice using sWGS of ctDNA. The low volume of blood required allows repeated serial collection of ctDNA samples from living, non‐anaesthetized mice and removes the need for terminal bleeding. Further, detailed modelling of tumour response indicates that the initial change in xTF in response to treatment is predictive of PFS and OS in mice (see Fig 3E), even when size measurements were stable (Fig 3D). The major limitation of manual size measurements in PDX experiments are confounding effects related to tissue necrosis, oedema and manual measurement error. Because of these factors, we do not expect to show precise correlation between ctDNA assays and manual tumour size measurements. Our data suggest that ctDNA measures (xTF values) provide an earlier and more sensitive readout of treatment response than tumour volume measures. This advocates the use of the xTF metric as a reliable and minimally invasive tool to monitor disease progression and to study treatment response in preclinical settings.

DBS are derived from whole blood: sensitive detection of ctDNA from DBS therefore requires removal of contaminating genomic DNA which otherwise significantly dilutes ctDNA signal (Heider *et al*, 2020b). In comparison to plasma samples, however, DBS have clear sampling advantages, since they do not require prompt centrifugation, and provide stable and space‐efficient storage of DNA for many years (Chaisomchit *et al*, 2005). DBS therefore have the potential to simplify sample collection and revolutionise study designs in both mice and patients:

In mice, the use of DBS has already been illustrated in pharmacokinetic studies (Wickremsinhe & Perkins, 2015) and has proven to conform with the 3Rs of animal welfare (Prescott & Lidster, 2017) by reducing the number of animals required per study, allowing sample collection at multiple time points, and improving the quality and quantity of data collected from a given mouse. In this study, blood samples were collected from the tail vein, which is considered a simple, humane and anaesthesia‐free approach (Durschlag *et al*, 1996). Alternative methods include submandibular or saphenous bleeding (Golde *et al*, 2005; Abatan *et al*, 2008) which, in contrast to tail vein bleeding, do not require the use of a mouse restrainer and will preserve the tail vein for drug administration. Although tail vein blood sampling has previously been optimised for disease monitoring, ctDNA assays were limited to PCR‐based experiments from plasma (Rago *et al*, 2007). In contrast, sWGS of DBS can be used to simultaneously assay ctDNA features and the copy number landscape of engrafted tumours at relatively low cost. We demonstrated sensitive and specific detection of xTFs from as a little as 1% of ctDNA. However, future work will determine sensitivity limits regarding both ctDNA concentration and the minimum sequencing depth/number of reads required for accurate ctDNA and copy number analyses from DBS samples. In addition, all experiments were performed in 3^rd^ or 4^th^ generation PDX animals and the potential effects on sensitivity from human stroma in that may be present in earlier PDX generations remains to be investigated. Further, we show that mouse‐ and human‐specific reads recapitulated the fragment size properties of human cfDNA and ctDNA, respectively (Underhill *et al*, 2016; Mouliere *et al*, 2018; Heider *et al*, 2020a), suggesting that mechanisms of cfDNA/ctDNA release into the blood stream are comparable in mice and humans. Our approach therefore provides a promising platform to study factors influencing ctDNA shedding, as well as other biochemical features of ctDNA, such as methylation and nucleosome profiles.

In the clinic, DBS‐based technologies may allow self‐collection at home (via a simple finger‐prick), obviating the need for additional phlebotomy or hospital visits, and thus improving test acceptability and study participation. While our approach proved to be highly sensitive for the detection and quantification of disease burden in PDX mice, it relied on the ability to identify tumour‐specific (human) ctDNA reads from DBS sequencing data using species‐specific read alignment. This will not be possible in DBS samples collected from cancer patients. However, similar sensitivities might be achieved by implementing other approaches that can improve sensitivity of ctDNA detection including fragmentomic (Mouliere *et al*, 2018; Cristiano *et al*, 2019) or epigenomic analyses (Lehmann‐Werman *et al*, 2016) and enrichment for patient‐specific mutations (personalised sequencing panels) (Abbosh *et al*, 2017; Parsons *et al*, 2020; Wan *et al*, 2020; Zviran *et al*, 2020; Kurtz *et al*, 2021), facilitating sensitive disease monitoring from small blood volumes in the clinic (Keller *et al*, 2021).

In summary, the use of DBS‐based WGS of ctDNA in murine models provides a powerful tool for preclinical disease monitoring and allows accurate monitoring of treatment response and the copy number landscape over time. Our approach provides new opportunities to study copy number‐driven tumour evolution and to investigate how treatment‐induced selection of copy number changes may result in treatment resistance. Most importantly, the use of DBS‐based ctDNA assays can simplify and improve study design in both mice and patients.

## Materials and Methods

### Generation of PDX mouse models

Solid tumour samples were obtained from patients enrolled in the OV04 study (CTCR‐OV04; REC ID: 08/H0306/61) at Addenbrooke’s Hospital, Cambridge. Informed consent was obtained from all study subjects, and all experiments conformed to the principles set out in the World Medical Association (WMA) Declaration of Helsinki and the Department of Health and Human Services Belmont Report.

Tumour samples were processed following standardised operating protocols as outlined in the OV04 study design and as previously described (preprint: Sauer *et al*, 2021) before surgically engrafting into 6 to 8‐week‐old female NOD. Cg‐Prkdc^scid^ Il2rg^tm1WjI^/SzJ (NSG) mice obtained from Charles River Laboratories. Mice were housed in ventilated cages with access to water and food *ad libitum*. All mouse work conducted was approved by and performed in accordance with the ethical regulations and guidelines of the Home Office UK and the CRUK CI Animal Welfare and Ethics Review Board (PPL number: PP7478310). Xenograft tissue processing and PDX passaging were performed as previously described (preprint: Sauer *et al*, 2021). In short, xenografting was performed either by subcutaneous surgical implantation (for first generation PDX mice) or subcutaneous injection of tumour cells from dissociated tumour tissues (for later PDX generations). Tumour‐bearing mice that reached their endpoint (tumour volumes of no more than 1,500 mm^3^) were culled via cervical dislocation or CO_2_ overexposure. Tumour tissues were dissected, processed as described above and re‐transplanted for expansion in serial generations for PDX biobank maintenance and model generation. All treatment experiments were performed on 3^rd^ generation (for patient lines 600, 828 and 831) or 4^th^ generation (for patient line 771) PDX mice.

### Treatment of mice

Treatment was initiated when engrafted tumours reached a size of approximately 500 mm^3^. Mice were randomised to either receive 50 mg/kg of carboplatin (dissolved in water for injections [WFI] and mannitol [10 mg/μl]), or 100 µl carboplatin vehicle/control (10 mg/ml of WFI diluted mannitol).

Mice were treated by tail vein injection on day 1 and day 8 and monitored until they reached their endpoint of 1,500 mm^3^ tumour volume, or if another health concern was raised.

### Measurement of tumour volume

Using callipers, the height (*h*), width (*w*) and depth (*d*) of the mouse tumours were measured in millimetres once a week and the tumour volume (mm^3^) was determined using the following formula:

$$\text{Tumour}\;\text{Volume}=\frac{1}{6}\pi \times hwd$$

### PDX tumour growth curve modelling

Heteroscedastic point‐wise random intercept linear mixed models were used to model the tumour growth (on the cube root scale) of both control and treated mice for each of the four patients included in this study. Heteroscedastic models were preferred as the (tumour growth) variance of treated mice appeared larger than the variance observed in the control mice.

For carboplatin‐treated mice within each patient group, the time points of the following two inflection points were determined by minimising the residual sum of squares (defined as the observed values minus the population expectation at a given time point) on the transformed scale:

- *t*_1_ = first inflection point: time point at which a treatment‐induced change in tumour growth can be observed for an average mouse of a given patient line, and
- *t*_2_ = second inflection point: time point at which a second (revertant) change in tumour growth (due to the end of treatment) could be observed for an average mouse of a given patient line,

where 0 corresponds to the day of start of treatment for each PDX mouse.

Different model checks were performed to ensure that the selected model for each patient showed homoscedastic and normally distributed random effect predictions and residuals. Since no obvious violation of the model assumptions were noted, chosen models were taken forward and statistical inference results (*P*‐values) trusted. *P*‐values were subjected to multiplicity correction adjustments for within‐patient analyses and comparisons.

### Collection and processing of dried blood spots

Blood spots were collected on day 1 (immediately before treatment start), 16 and 29 for PDX mice. Mice were immobilized in a stretcher/restrainer before ticking the tail with a needle. Upon squeezing the tail, ~ 50 µl of blood were collected using a capillary lined with EDTA. The capillary was emptied into a 1.5 ml microfuge tube and the blood was spotted onto Whatman FTA™ Classic Cards (Merck), and left to air dry for at least 15 min before storing at room temperature. For control experiments, blood spot samples were also collected from non‐tumour‐bearing (healthy) NSG mice during terminal bleeds via cardiac puncture. Collection of DBS from the tail vein of living non‐tumour‐bearing mice was not possible owing to project licence limitations. Instead, terminal bleeds were performed on non‐genetically modified mice that were removed from genotyping experiments via Schedule 1 procedures using syringes pre‐flushed with EDTA, and 50 µl of collected blood was subsequently spotted onto Whatman FTA™ Classic Cards. Again, cards were left to dry for 15 min.

In addition, dried blood spot samples were derived from five independent HGSOC patients (for use in dilution experiment; see Appendix Table S2 for patient information) by applying ~ 50 µl of blood collected in K2‐EDTA tubes to Whatman FTA™ Classic Cards.

DBS samples were stored at room temperature for a median of 124 days (IQR 80–168 days) prior to further processing. Nucleic acids have been shown to remain stable for years on filter paper cards (Chaisomchit *et al*, 2005), allowing safe storage of DBS samples over several months to years for batch processing and sequencing.

### Shallow Whole‐Genome Sequencing (sWGS)

#### Fresh frozen tumour tissue samples

Fresh frozen tissue pieces were homogenised using soft tissue homogenising CK14 tubes containing 1.4 mm ceramic beads (Bertin) on the Precellys tissue homogeniser instrument (Bertin). Lysates were subjected to DNA extraction using the AllPrep DNA/RNA Mini Kit (Qiagen) following manufacturer’s recommendations, and DNA was sheared to a fragment length of 200 bp using the Covaris LE220 (120 s at room temperature; 30% duty factor; 180 W peak incident power; 50 cycles per burst).

Using the SMARTer Thruplex DNA‐seq kit (Takara), 50 ng of sheared DNA was prepared for sequencing following the recommended instructions with samples undergoing five PCR cycles for unique sample indexing and library amplification. Subsequently, AMPure XP beads were used (following manufacturer’s recommendations) to clean prepared libraries, which were then quantified and quality‐checked using the Agilent 4200 TapeStation System (G2991AA). Pooled libraries were sequenced at low coverage on the HiSeq 4000 with single 50 bp reads, at the CRUK CI Genomic Core Facility. Sequencing reads were aligned to the 1000 Genomes Project GRCh37‐derived reference genome using the “BWA” aligner (v.0.07.17) with default parameters.

#### Dried blood spot samples

DNA from dried blood spots was extracted using the Qiagen Investigator kit (Qiagen) as previously described (Heider *et al*, 2020b) and eluted in 50 µl elution buffer. High molecular weight genomic DNA (gDNA) was removed using right‐side size selection with AMPure XP beads at a 1:1 and 7:1 bead:sample ratio (Beckman Coulter) described previously (Heider *et al*, 2020b), and eluted in 25 µl water.

Before undergoing ThruPLEX Tag‐seq library preparation (Takara), samples were concentrated to 10 µl using a vacuum concentrator (SpeedVac). Samples were amplified for 14–16 cycles before undergoing the recommended bead clean up to remove remaining adapters. Quality control for library generation and quantification was done using a TapeStation (Agilent) before samples were submitted for sequencing on a NovaSeq 6000 SP (Illumina, paired‐end 150 bp) at the CRUK CI Genomic Core Facility.

### Analysis of dried blood spot sequencing data

Blood spot sequencing data was aligned to the human (hg19) and mouse genome (mm10) using Xenomapper (Wakefield, 2016). Reads overlapping with black‐listed regions for both human and mouse genomes were removed using the bedtools intersect function. Using Picard CollectInsertSizeMetrics, insert sizes were determined for the specific output files for each species. We computed a human ratio, that we call xenograft Tumour Fraction (xTF), for each sample by taking the total number of human reads > 30 bp fragment length and divided it by all reads (mouse and human) > 30 bp fragment length. Fragments below 30 bp fragment length were excluded from the analysis as they tended to be noisy.

### Dilution series

To test the sensitivity and specificity of the human ratio metric, an *in silico* dilution experiment was performed using dried blood spot sequencing reads from five independent OV04 HGSOC patients (i.e. human reads only) and a healthy (non‐tumour‐bearing) NSG mouse (i.e. mouse reads only). First, fastq files were aligned to the human (hg19) and mouse (mm10) reference genomes, respectively, to account for differences in sample quality, and to remove unmappable and duplicate reads. Resulting bam files were converted back to paired‐end fastq files using the bedtools bamToFastq conversion utility. Mouse and human fastq files were then downsampled and merged to generate a seven‐point dilution series containing 1, 2, 5, 7, 10, 15 and 25% of human reads diluted in mouse reads for each of the 5 OV04 patients (35 samples in total) with a total read count of 6.5 × 10^6^. Paired‐end fastq file pairs were then analysed with the Xenomapper pipeline, and human ratios (xTFs) were estimated as described above. Estimated xTFs were then compared with expected human ratios based on *in silico* dilution mixtures.

### Absolute copy number analyses

We used the QDNAseq R package (Scheinin *et al*, 2014) (v1.24.0) to count reads within 30 and 500 kb bins, followed by read count correction for sequence mappability and GC content, and copy number segmentation. Resulting relative copy number data were then subjected to downstream analyses using the Rascal R package (preprint: Sauer *et al*, 2021) for ploidy and cellularity estimation and absolute copy number fitting as previously described (preprint: Sauer *et al*, 2021). For dried blood spot (DBS) samples, ploidy information from fitted tumour tissue samples from the same patient line were used to guide accurate ACN fitting. Note that DBS samples from healthy (non‐tumour‐bearing) mice were automatically fitted to diploid ACN fits due to the absence of tumour reads and detectable somatic copy number aberrations (SCNAs).

Following ploidy and cellularity estimation, absolute copy number (ACN) profiles were generated for tumour tissues and DBS samples and subsequently correlated/compared across each 500 kb bin.

Putative driver amplifications were detected and identified using the Catalogue of Somatic Mutations In Cancer (COSMIC; https://cancer.sanger.ac.uk/cosmic/help/cnv/overview) definitions and thresholds for high level amplifications and homozygous deletions: Gain: average genome ploidy ≤ 2.7 *and* total copy number ≥ 5; or average genome ploidy > 2.7 *and* total copy number ≥ 9. Loss: average genome ploidy ≤ 2.7 *and* total copy number = 0; or average genome ploidy > 2.7 *and* total copy number < (average genome ploidy − 2.7). Copy number signatures, as shown in Fig EV1E, were estimated as previously described (Macintyre *et al*, 2018).

### Tagged‐Amplicon Sequencing (TAm‐Seq)

Small indels and single‐nucleotide variants were assessed across the coding regions of *TP53*, *BRCA1*, *BRCA2*, *MLH1*, *MSH2*, *MSH6*, *NF1*, *PMS2*, *PTEN*, *RAD51B*, *RAD51C*, *RAD51D* and mutation hot spot regions for *BRAF*, *EGFR*, *KRAS* and *PIK3CA* using the Tagged‐Amplicon deep sequencing technology as previously reported (Forshew *et al*, 2012). Briefly, libraries were prepared in 48.48 Juno Access Array Integrated Fluidic Circuits chips (Fluidigm, PN 101‐1926) on the IFC Controller AX instrument (Fluidigm), and libraries were sequenced by the CRUK CI Genomics Core Facility using 150 bp paired‐end mode on either the NovaSeq 6000 (SP flowcell) or HiSeq 4000 system. Reads were aligned to the GRCh37 reference genome using the ‘BWA‐MEM’ aligner and variant calling was performed as previously described (Piskorz *et al*, 2016).

### Haematoxylin and Eosin (H&E) and immunohistochemical p53 staining

H&E and immunohistochemical staining of p53 were carried out by the CRUK CI Histopathology Core Facility. H&E sections were stained following the Harris H&E staining protocol using a multistainer instrument (Leica ST5020). p53 staining was performed on 3 µm FFPE sections using the Leica Bond Max fully automated IHC system. Antigen retrieval was performed using sodium citrate for 30 min, and p53 was stained using the M7001 Dako p53 antibody (1:1,000; RRID:AB_2206626).

## Author contributions

**Carolin M Sauer:** Conceptualization; Resources; Data curation; Software; Formal analysis; Supervision; Funding acquisition; Validation; Investigation; Visualization; Methodology; Writing—original draft; Project administration; Writing—review & editing. **Katrin Heider:** Conceptualization; Resources; Data curation; Software; Formal analysis; Supervision; Funding acquisition; Validation; Investigation; Visualization; Methodology; Writing—original draft; Project administration; Writing—review & editing. **Jelena Belic:** Resources; Data curation; Investigation. **Samantha E Boyle:** Resources; Investigation; Methodology. **James A Hall:** Resources; Investigation; Methodology. **Dominique‐Laurent Couturier:** Resources; Formal analysis; Writing—review & editing. **Angela An:** Resources; Investigation; Writing—review & editing. **Aadhitthya Vijayaraghavan:** Resources; Data curation; Software; Formal analysis; Methodology. **Marika AV Reinius:** Data curation; Writing—review & editing. **Karen Hosking:** Data curation. **Maria Vias:** Resources; Supervision; Investigation; Methodology; Project administration; Writing—review & editing. **Nitzan Rosenfeld:** Conceptualization; Supervision; Funding acquisition; Writing—original draft; Project administration; Writing—review & editing. **James Derek Brenton:** Conceptualization; Supervision; Funding acquisition; Writing—original draft; Project administration; Writing—review & editing.

## Disclosure and competing interests statement

Several of the authors are inventors and contributors on patents relating to methods for ctDNA analysis including methods described and used in this study. N.R. is an officer of Inivata Ltd. which commercialises ctDNA assays. J.D.B. is a founder of Tailor Bio. Both, Inivata and Tailor Bio, had no role in the conceptualisation or design of the preclinical study, statistical analysis, or decision to publish the manuscript.

## Supporting information



Appendix

Expanded View Figures PDF

Source Data for Appendix

Source Data for Figure 2

Source Data for Figure 3

## Data Availability

Sequencing data from DBS and tumour tissue samples have been deposited in the European Genome‐phenome Archive (EGA) database with study accession number EGAS00001006134 (https://ega‐archive.org/studies/EGAS00001006134). Estimated xTF values and tumour volumes for all mice included in this study can be downloaded from the source data files.
