# Supplementary material for: Longitudinal monitoring of disease burden and response using ctDNA from dried blood spots in xenograft models
Source: EMBO Mol Med. 2022 Jun 13;14(8):e15729. doi: 10.15252/emmm.202215729 (PMC9358392; doi:10.15252/emmm.202215729)
Supplement: Supplementary file 1 — Appendix [file EMMM-14-e15729-s001.pdf]

APPENDIX

Longitudinal monitoring of disease burden and response using ctDNA from dried blood spots in xenograft models

Carolin M Sauer[1,2#*], Katrin Heider[1,2#], Jelena Belic[1,2], Samantha E Boyle[1,2], James A Hall[1,2], Dominique-Laurent Couturier[1,3], Angela An[1,2], Aadhitthya Vijayaraghavan[1,2], Marika AV Reinius[1,2], Karen Hosking[2], Maria Vias[1,2], Nitzan Rosenfeld[1,2$*], James D Brenton[1,2,4,5$*]

Contents

Appendix Figures and Tables 2

Appendix Figure S1 – Fragment size profile of reads obtained from healthy non-tumour bearing mouse DBS. 2

Appendix Figure S2 – Examples of copy number profiles from DBS and tumour tissue samples for each of the four patient lines. 3

Appendix Figure S3 – Correlation of SCNAs and detection of driver amplifications in DBS. 4

Appendix Figure S4 – Histological features of patient and PDX tumour tissues. 6

Appendix Figure S5 – Molecular features of patient and PDX tumour tissues. 8

Appendix Figure S6 – Correlation between xTF values and tumour volumes. 9

Appendix Table S1 - Tumour growth modelling 10

Appendix Table S2 – Patient overview for DBS dilution series analysis 12

Appendix Figures and Tables

Appendix Figure S1 – Fragment size profile of reads obtained from healthy non-tumour bearing mouse DBS.

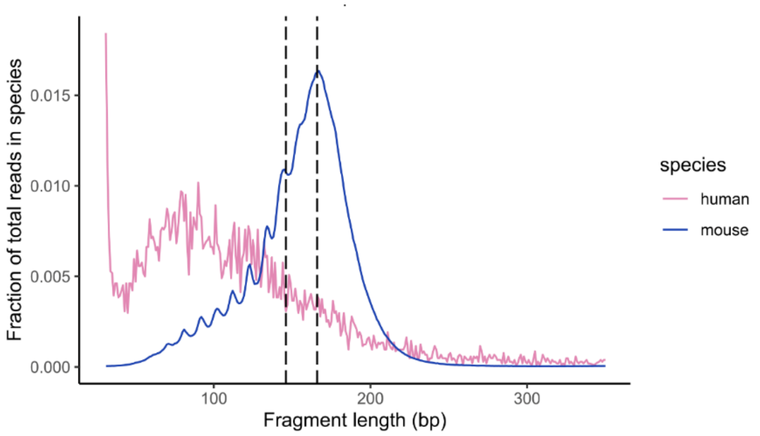

Fragment length distributions of human- (pink) and mouse- (blue) specific reads from a DBS sample collected from a non-tumour bearing healthy control mouse. Two vertical lines indicate 146 and 166 bp, the observed peaks for ctDNA and cfDNA, respectively. Note that fragments <30bp fragment length were excluded from all DBS analyses.

Appendix Figure S2 – Examples of copy number profiles from DBS and tumour tissue samples for each of the four patient lines.

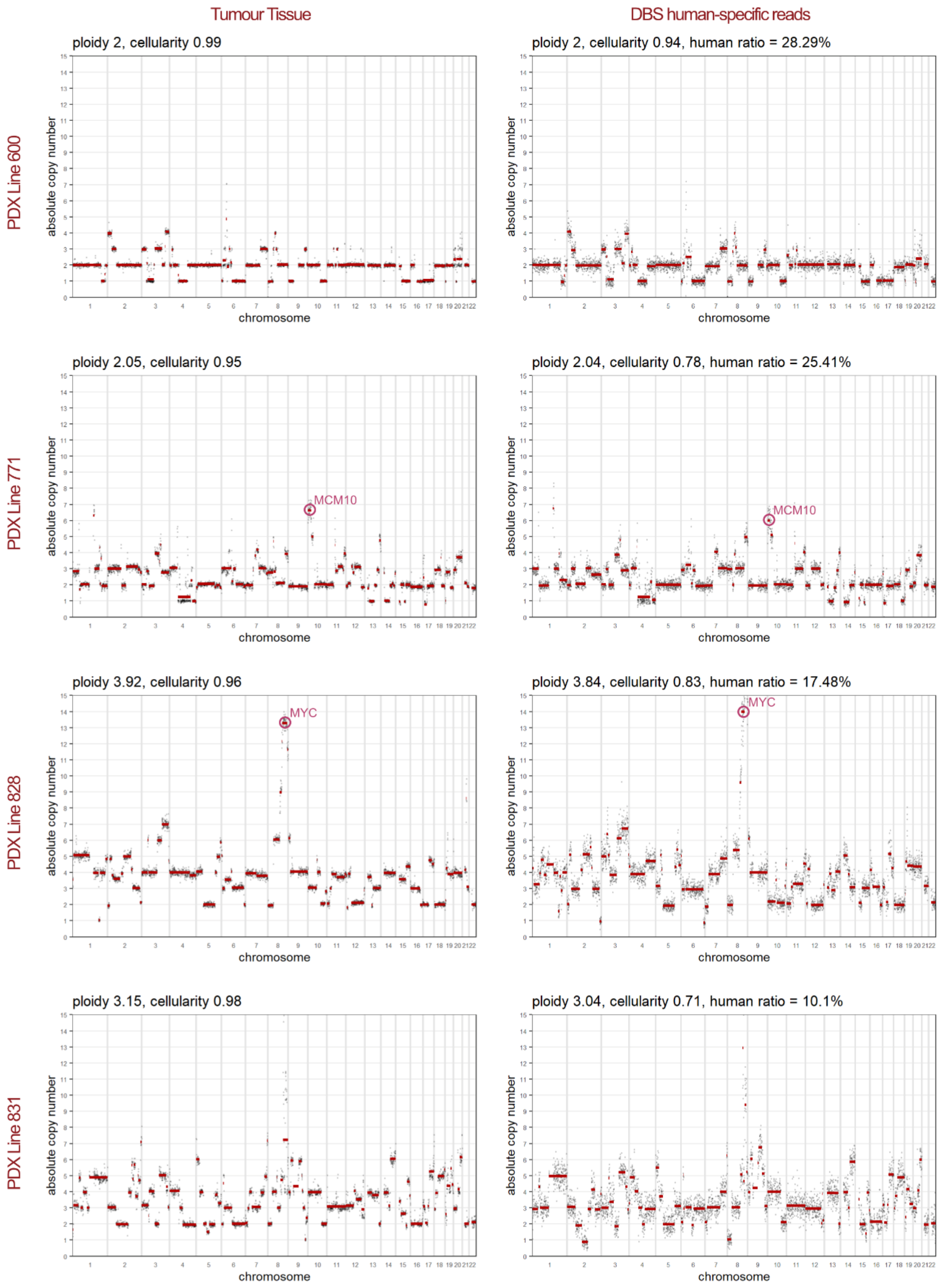

Comparison of absolute copy number (ACN) profiles obtained from sWGS data from a first generation PDX tumour tissues (left panel) and dried blood spot (DBS) samples from PDX mice (right panel) for patient 600, 771, 828, and patient 831. *MCM10* and *MYC* amplifications were detected in patients 771 and 828 samples, respectively, and are highlighted by pink circles.

Appendix Figure S3 – Correlation of SCNAs and detection of driver amplifications in DBS.

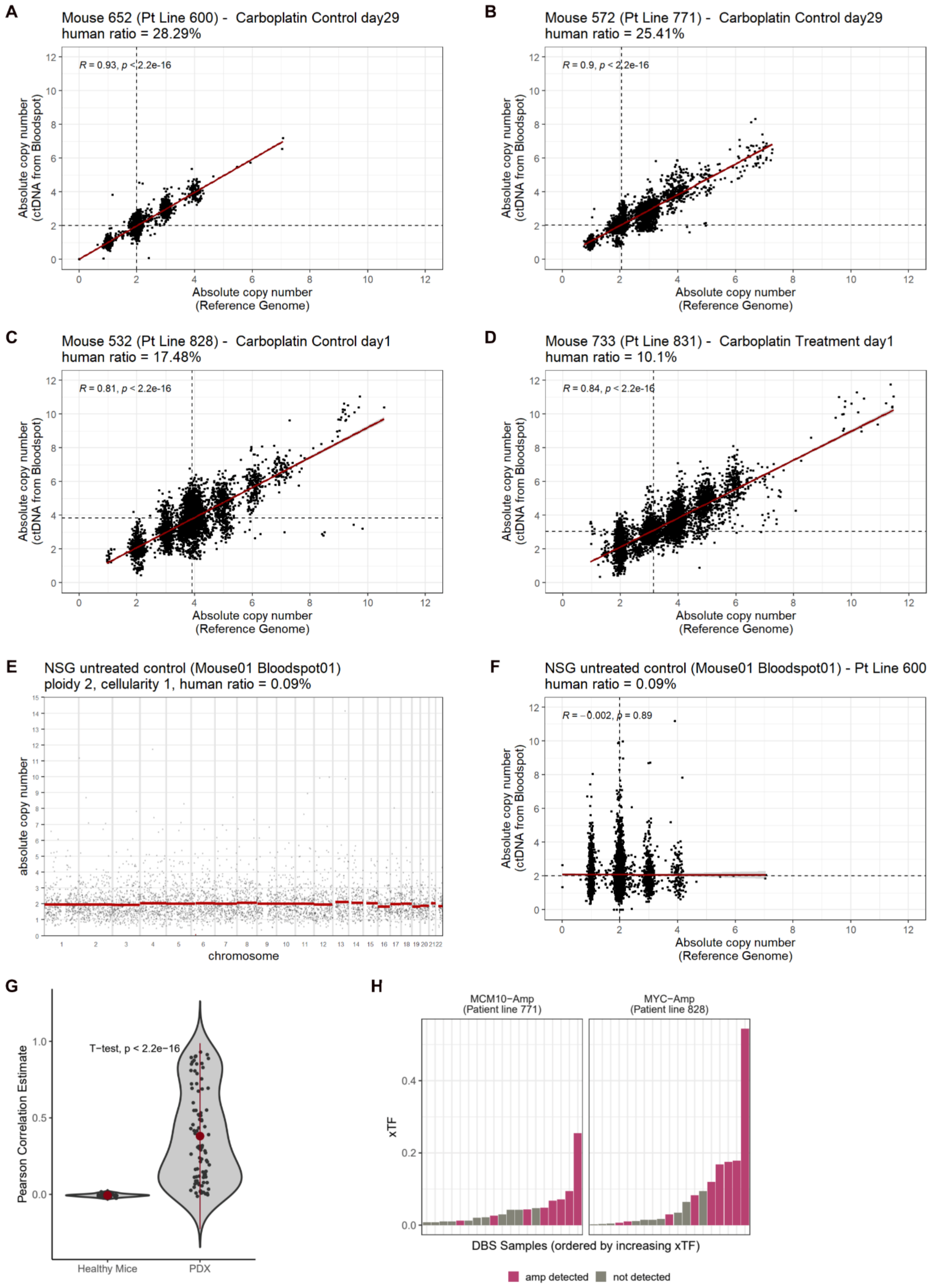

Pearson correlation plots comparing absolute copy number (ACN) profiles shown in **Supplementary Figure 1** for patient lines 600 (**A**), 771 (**B**), 828 (**C**) and 831 (**D**). Grey dashed lines indicated fitted ploidies for ACN profiles obtained from dried blood spots (horizontal line) and PDX tumour tissues

(vertical line). (**E**) Example of an ACN profile obtained from a blood spot sample from a healthy non-tumour bearing mouse with an xTF of 0.0009 (0.09%). (**F**) Example correlation plot comparing the ACN profiles obtained from a blood spot from a healthy non-tumour bearing mouse and the first-generation tumour tissue from PDX line 600. (**G**) Comparison of Pearson correlation estimates (correlating ACN profiles from blood spot and tumour tissue samples) between healthy (non-tumour bearing) and PDX mice (Welch t-test, $p < 2.2. x10^{-16}$; Wilcoxon test, $p < 2.2. x10^{-16}$). (**H**) Waterfall plot indicating samples for which putative driver amplifications were detected using COSMIC specifications (**see Methods**). Samples are arranged by increasing xTF values. Gene amplifications are detected in samples indicated in pink.

Appendix Figure S4 – Histological features of patient and PDX tumour tissues.

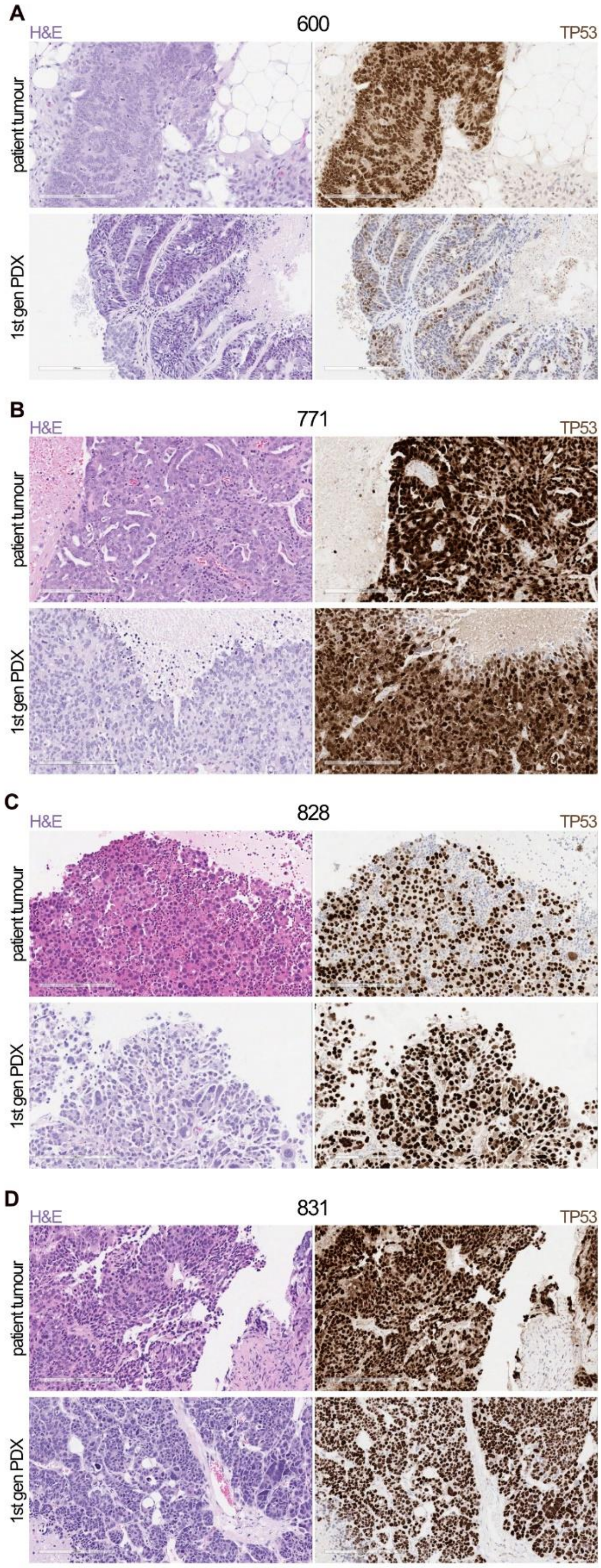

Comparison of haematoxylin and eosin (H&E, left panel) and p53 immunohistochemistry (IHC, right panel) staining between patient and PDX tumour tissues for (**A**) patient 600, (**B**) patient 771, (**C**) patient 828, and (**D**) patient 831. The patient tumour is shown on the top, and the PDX tumour is shown on the bottom of each figure panel. Representative tumour regions were selected for illustration purposes and

are from similar regions but are not exactly coregistered. Images of the full face sections and zoomed-in regions are provided in the source data. Scale = 200µm.

Appendix Figure S5 – Molecular features of patient and PDX tumour tissues.

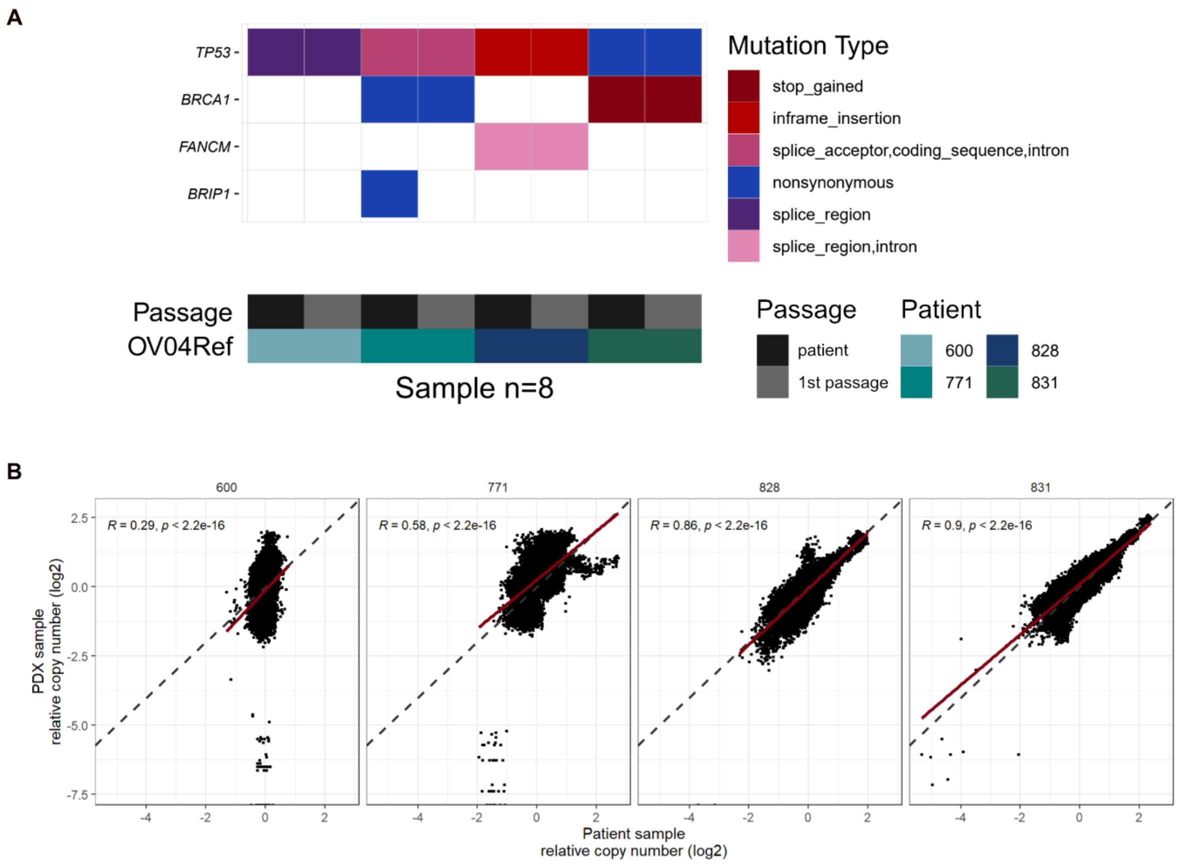

(**A**) Tagged-Amplicon Sequencing (TAm-Seq) mutational data comparing patient tumour tissues, and first generation PDX tumour tissues for each of the four patient lines, as indicated by different shades of blue/green. Patient tissues are indicated by dark grey blocks, whereas corresponding PDX tumour tissues are indicated by light grey blocks. (**B**) Comparison of relative copy number data (log2 scale) obtained from shallow whole genome sequencing between the original patient tumour tissue, and the first generation PDX tumour tissue for each of the four patient lines. Diagonal grey dashed line indicates a slope of 1. Note that the original tumour tissue for patient 600 had very low tumour purity (<10%) resulting in a mostly flat copy number profile and consequently a poorer correlation when compared to the associated pure PDX tumour tissue.

Appendix Figure S6 – Correlation between xTF values and tumour volumes.

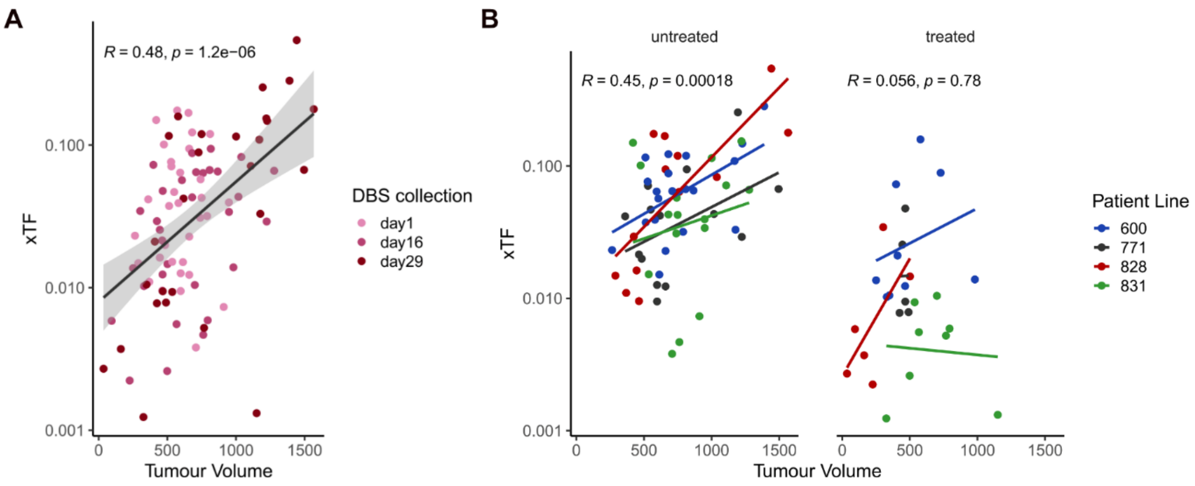

Correlation between xTF values and tumour volumes of the nearest matched time-point for (**A**) all analysed samples combined (Spearman R = 0.48, p =1.2×10^{-6}), and (**B**) untreated (Spearman R = 0.45, p = 0.00018) vs. carboplatin-treated (Spearman R = 0.056, p = 0.78) PDX mice coloured by patient line.

Appendix Table S1 - Tumour growth modelling

The following Table shows the unadjusted ('raw' columns) and adjusted ('adj' columns) p-values and star-based significance levels for each patient line corresponding to the following comparions:

Contrast 1: Difference in tumour volume between the control and treatment groups at treatment start
Contrast 2: Difference in tumour growth between the control and treatment groups before time inflection point t_1 (i.e. before start of treatment effect)
Contrast 3: Difference in tumour growth before and after time inflection point t_1 for treated mice (a significant effect would correspond to a significant tumour growth change due to treatment)
Contrast 4: Difference in tumour growth before and after inflection point t_2 for treated mice (a significant effect would correspond to a significant tumour growth change due to end of treatment)
Contrast 5: Difference in tumour growth before inflection point t_1 and after inflection point t_2 for treated mice (a significant effect would correspond to a significant tumour growth change before and after treatment effect)
Contrast 6: Difference in tumour growth between the control and treatment groups after inflection point t_2 (where the tumour growth is assumed constant through time for mice of the control group)

Patient line 600

contrasts	pval.raw	sig.raw	pval.adj	sig.adj
Contrasts1	0.700861		1	
Contrasts2	0.9252195		1	
Contrasts3	1.88528E-05	***	0.000364298	***
Contrasts4	3.53503E-10	***	5.6196E-09	***
Contrasts5	0.8592234		1	
Contrasts6	0.5726736		1	

Patient line 771

contrasts	pval.raw	sig.raw	pval.adj	sig.adj
Contrasts1	0.2710158		1	
Contrasts2	0.4120366		1	
Contrasts3	1.22636E-09	***	1.53545E-08	***
Contrasts4	3.43222E-08	***	4.72356E-07	***
Contrasts5	0.1996257		1	
Contrasts6	0.02762937	*	0.4921333	

Patient line 828

contrasts	pval.raw	sig.raw	pval.adj	sig.adj
Contrasts1	0.2054286		1	
Contrasts2	0.5641273		1	
Contrasts3	6.66134E-16	***	1.5099E-14	***
Contrasts4	0	***	0	***
Contrasts5	0.2650988		1	
Contrasts6	0.3385248		1	

Patient line 831

contrasts	pval.raw	sig.raw	pval.adj	sig.adj
Contrasts1	0.9144219		1	
Contrasts2	0.1758687		1	
Contrasts3	1.51909E-10	***	2.21377E-09	***
Contrasts4	6.17251E-08	***	1.14541E-06	***
Contrasts5	0.00387063	**	0.07970715	.
Contrasts6	0.09430599	.	1	

Appendix Table S2 – Patient overview for DBS dilution series analysis

Patient	StudyID	Histology	Stage	Barcode	SequencingID
Patient 1	1132	HGSOC	IVB	D708tp-D501tp	SLX-10615
Patient 2	461	HGSOC	IIIC	D708tp-D502tp	SLX-10615
Patient 3	1117	HGSOC	IC	D708tp-D503tp	SLX-10615
Patient 4	1020	HGSOC	IIIC	D708tp-D504tp	SLX-10615
Patient 5	628	HGSOC	IV	D708tp-D505tp	SLX-10615
