# Supplementary material for: Longitudinal monitoring of disease burden and response using ctDNA from dried blood spots in xenograft models
Source: EMBO Mol Med. 2022 Jun 13;14(8):e15729. doi: 10.15252/emmm.202215729 (PMC9358392; doi:10.15252/emmm.202215729)
Supplement: Supplementary file 2 — Expanded View Figures PDF [file EMMM-14-e15729-s004.pdf]

Expanded View Figures

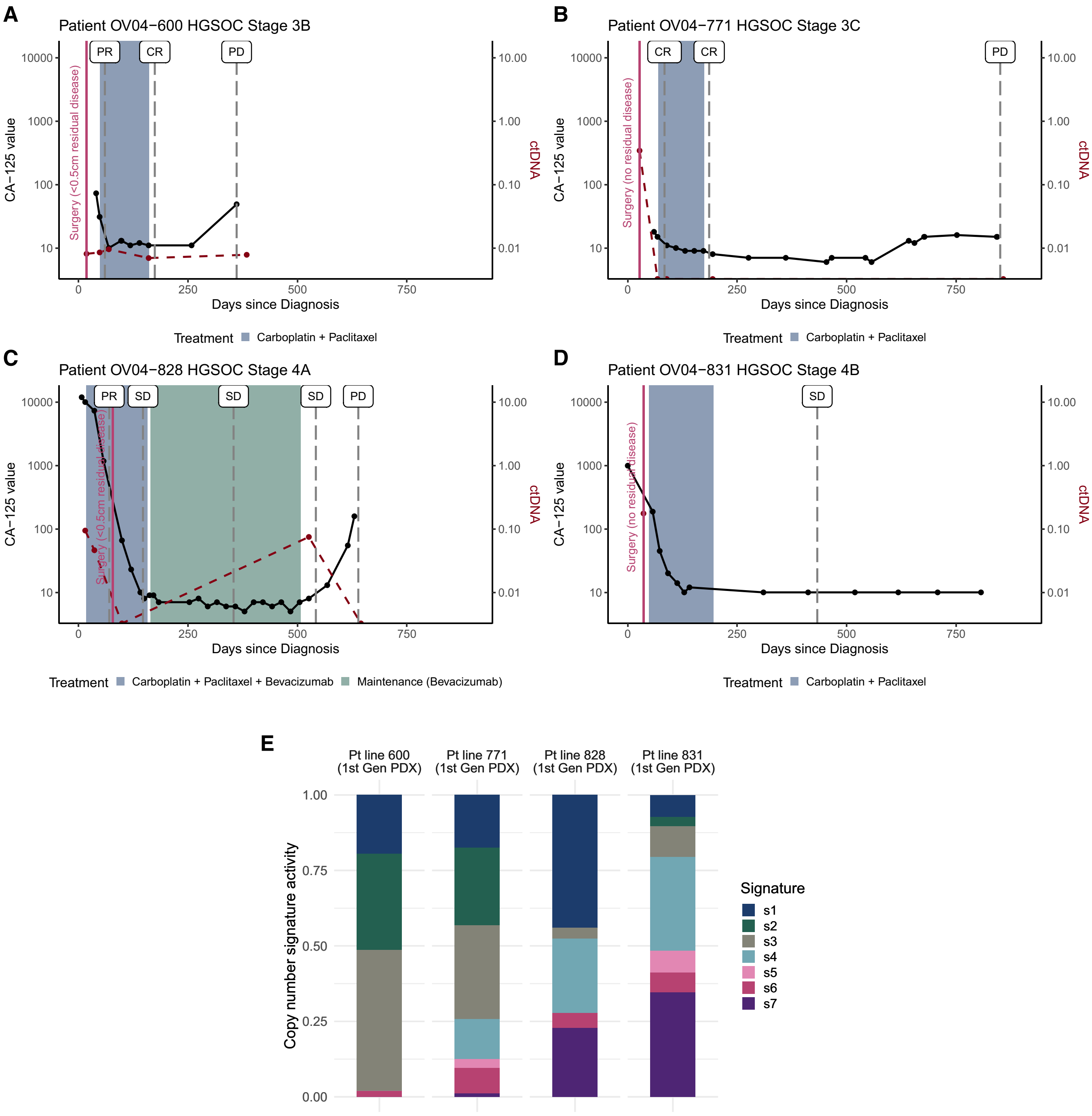

Figure EV1. Clinical treatment response, surgery outcome, time until progression and copy number signatures for preclinical study patients.

A–D CA-125 values (black line), treatment response assessments estimated via CT scans (vertical grey dashed lines), and ctDNA data, where available (red dashed line), for HGSOC patients 600, 771, 828 and 831, respectively, over time. Surgery and additional treatment regimens are indicated by a pink vertical line and shaded boxes, respectively (CR, Complete Response; PR, Partial Response; SD, Stable Disease; PD, Progressive Disease).

E Stacked bar plots showing copy number signature activities for first generation PDX tissues derived from the four patients (patient 600, 771, 828 and 831) used in the preclinical HGSOC study.

© 2022 The Authors

A

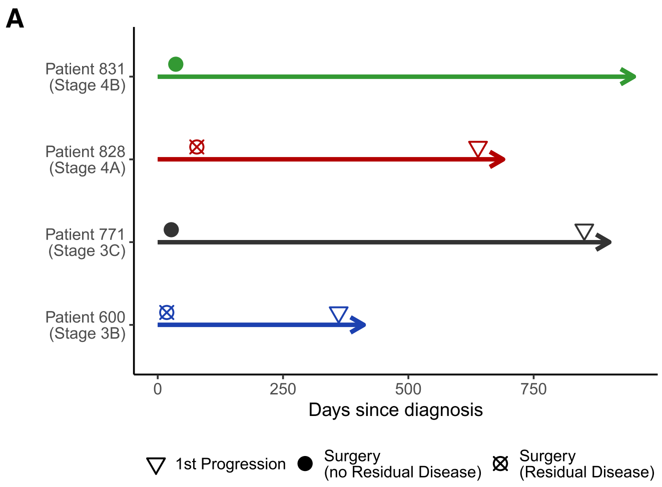

B

PATIENT								PDX MODELS		
ID	Stage	Age at diagnosis	Time to relapse (days)	Overall survival (days)	*TP53*	*BRCA1/2*	other	Duration treatment effect (days)	Time to regrowth (days)	Time to endpoint (days; treat/ctrl)
**600**	IIIB	46	361	1322	c.994-1G>C	NA	NA	22	23	67.6 / 39.4
**771**	IIIC	51	851	NA	c.783-5_785del	NA	ER+	28	33	62.0 / 31.6
**828**	IVA	60	639	1662	c.823_825dup	sBRCA1 translocation	*MYC* Amp	41	44	151.3 / 28
**831**	IVB	61	NA	NA	c.584T>C	gBRCA1m	ER+	39	41	67.8 / 35.0

Figure EV2. Overview of preclinical patients and PDX lines.

A Overview of clinical timelines until first disease progression in the four HGSOC patients.

B Table summarising clinical, genomic and PDX tumour growth kinetics information for all four patients included in this study. Patient 600, 771 and 831 underwent primary surgery prior to treatment, whereas patient 828 received neoadjuvant treatment prior to interval debulking surgery and further treatment. Both patient 600 and 828 had residual disease following surgery.

 © 2022 The Authors
