# Supplementary figures and images for: Longitudinal monitoring of disease burden and response using ctDNA from dried blood spots in xenograft models
Source: EMBO Mol Med. 2022 Jun 13;14(8):e15729. doi: 10.15252/emmm.202215729 (PMC9358392; doi:10.15252/emmm.202215729)

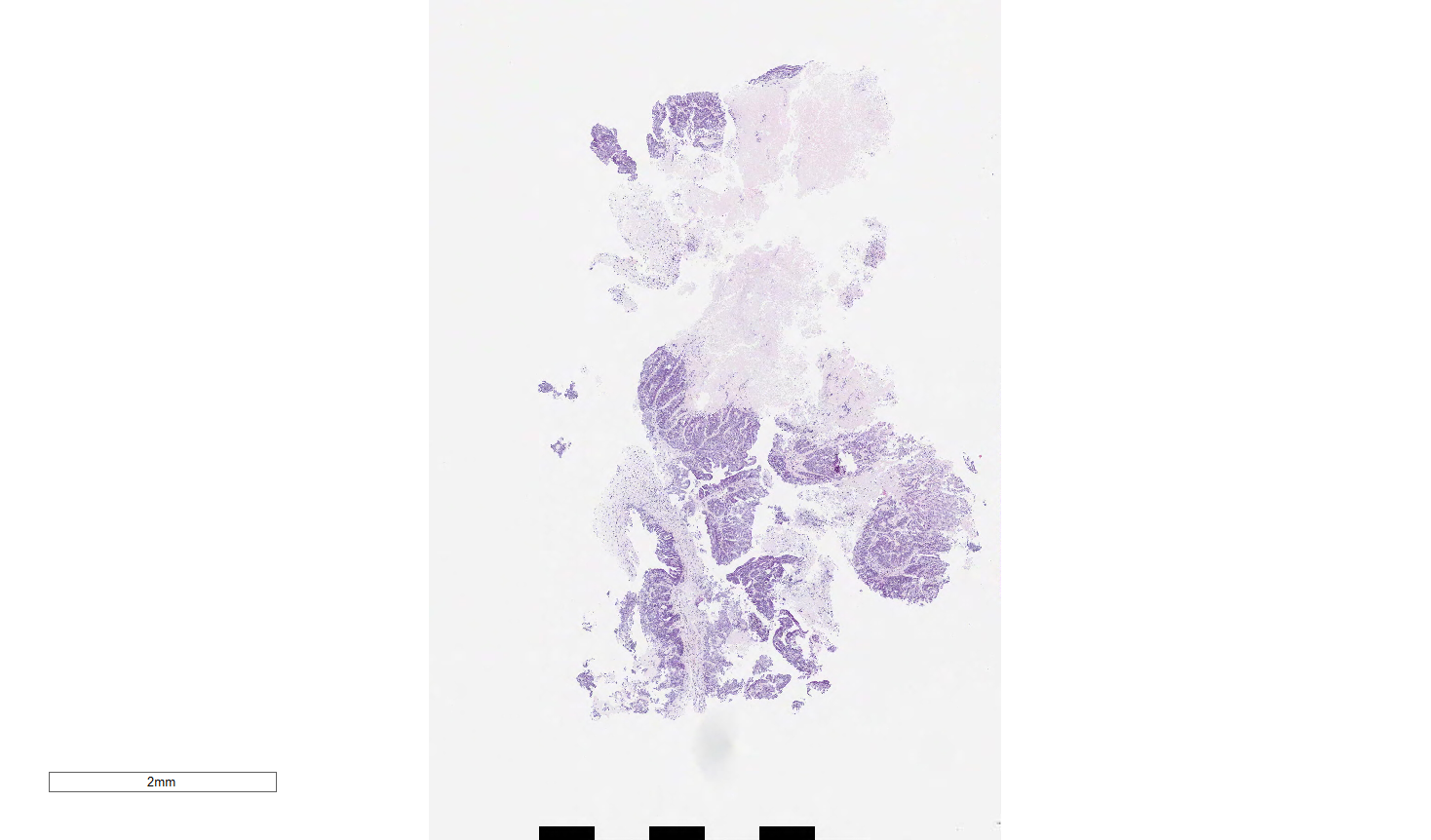

Supplement: Supplementary file 3 — Source Data for Appendix [file EMMM-14-e15729-s005.zip › 600/Mouse 83 JB257 - H+E whole.tif]

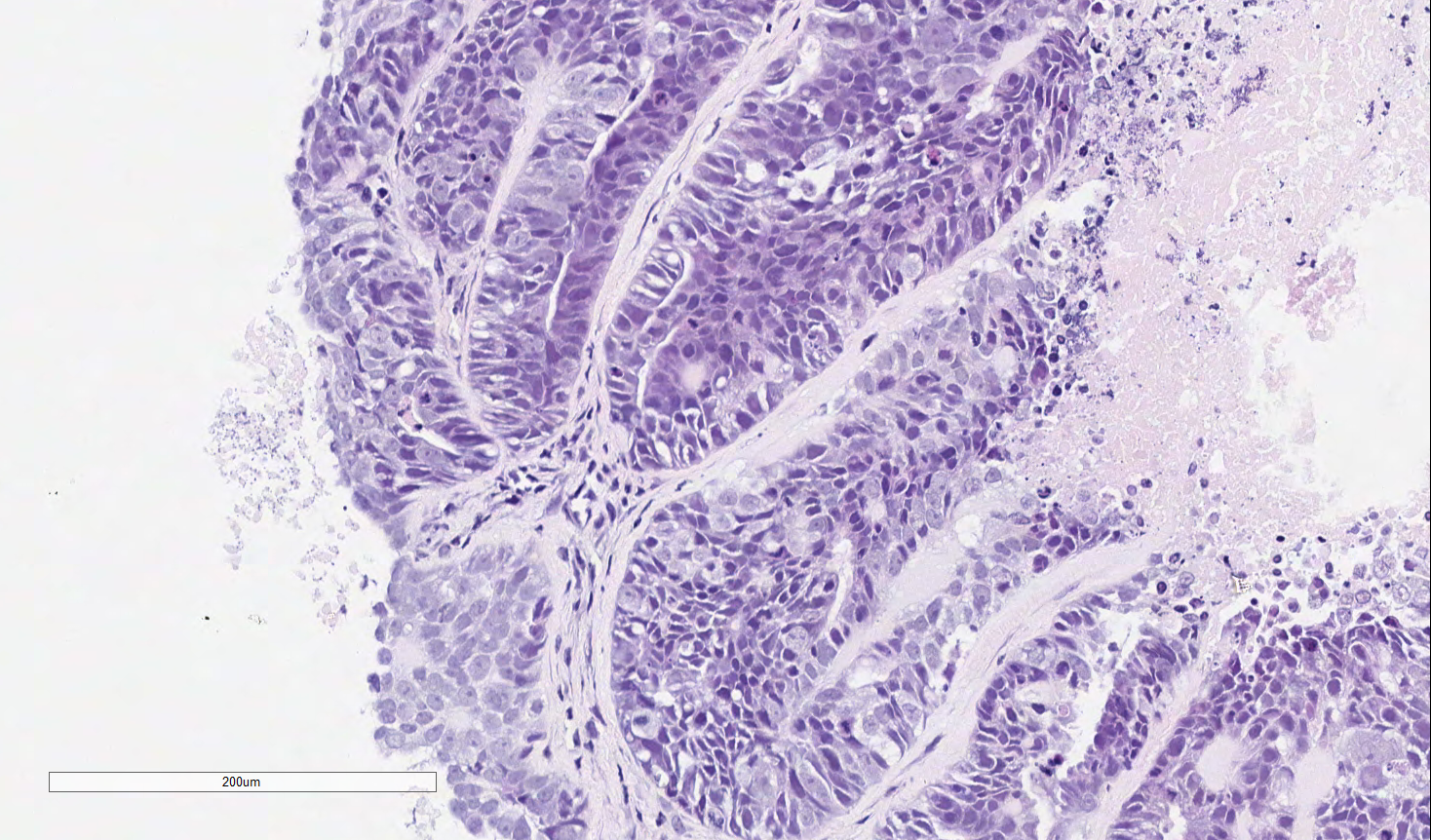

Supplement: Supplementary file 3 — Source Data for Appendix [file EMMM-14-e15729-s005.zip › 600/Mouse 83 JB257 - H+E zoom.tif]

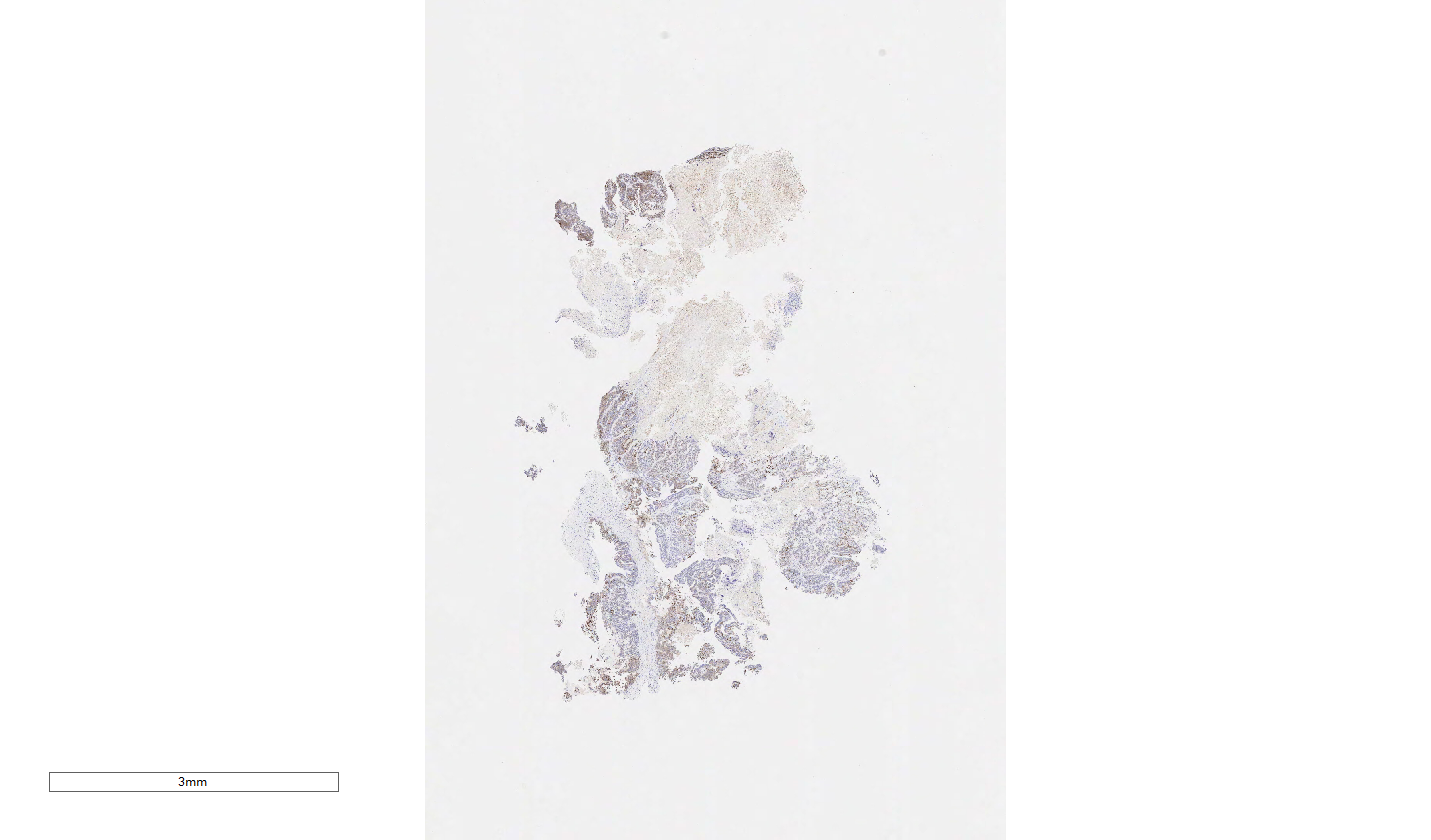

Supplement: Supplementary file 3 — Source Data for Appendix [file EMMM-14-e15729-s005.zip › 600/Mouse 83 JB257 - p53 whole.tif]

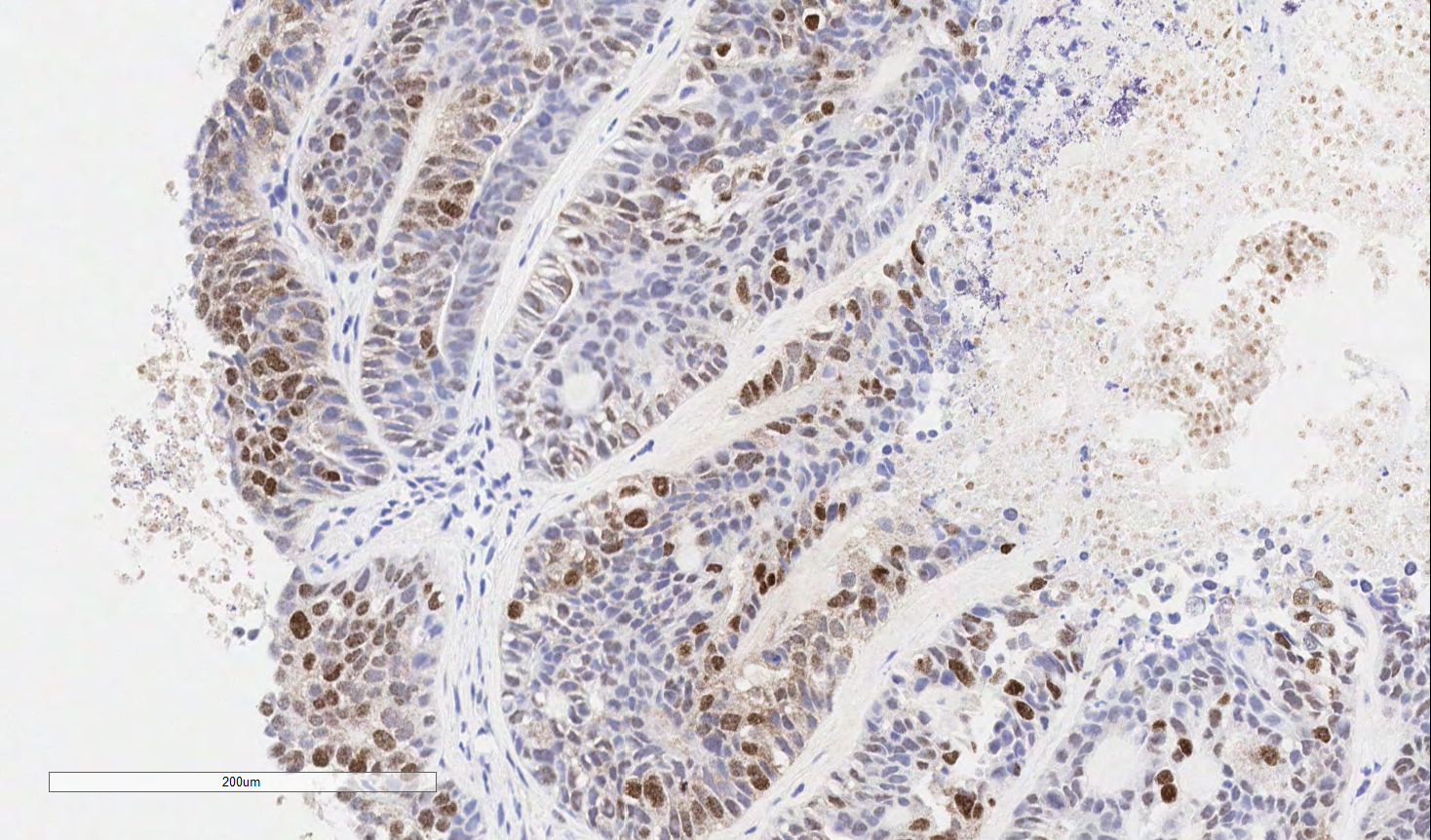

Supplement: Supplementary file 3 — Source Data for Appendix [file EMMM-14-e15729-s005.zip › 600/Mouse 83 JB257 - p53E zoom.tif]

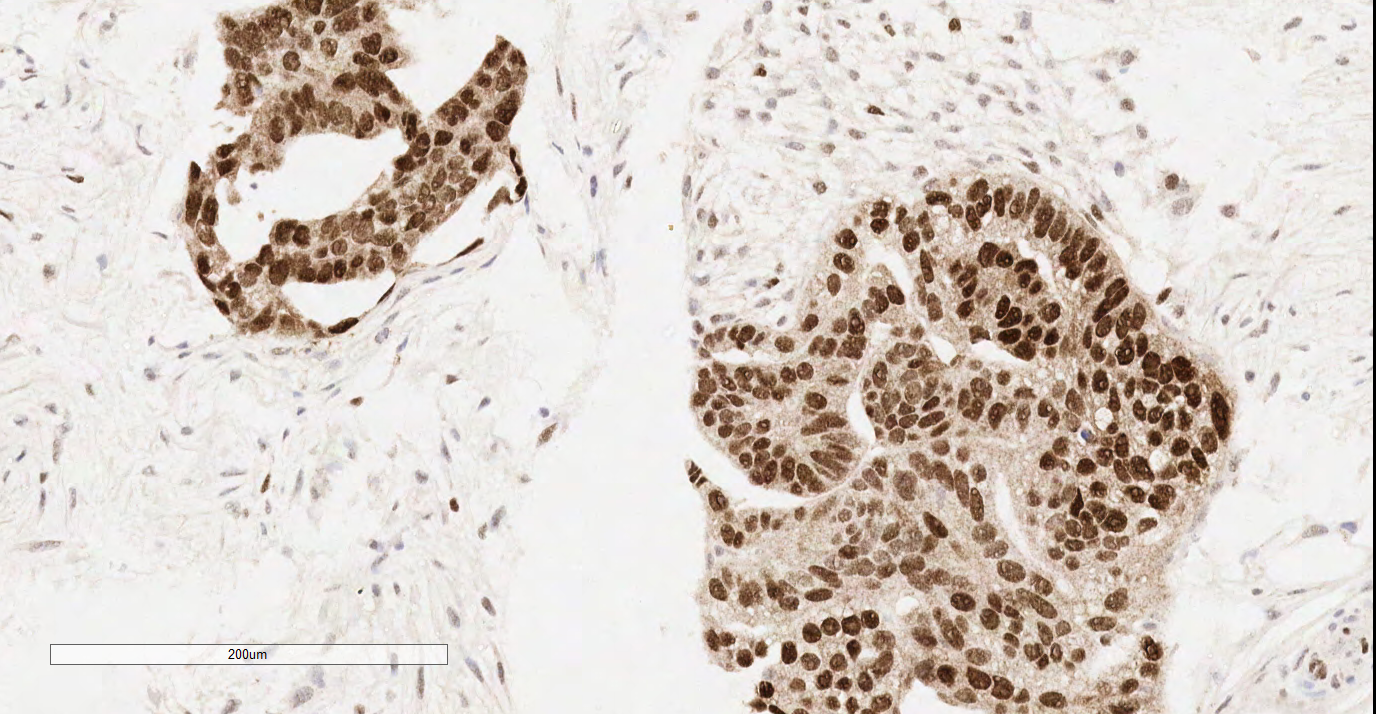

Supplement: Supplementary file 3 — Source Data for Appendix [file EMMM-14-e15729-s005.zip › 600/paitent JB257 - p53.tif]

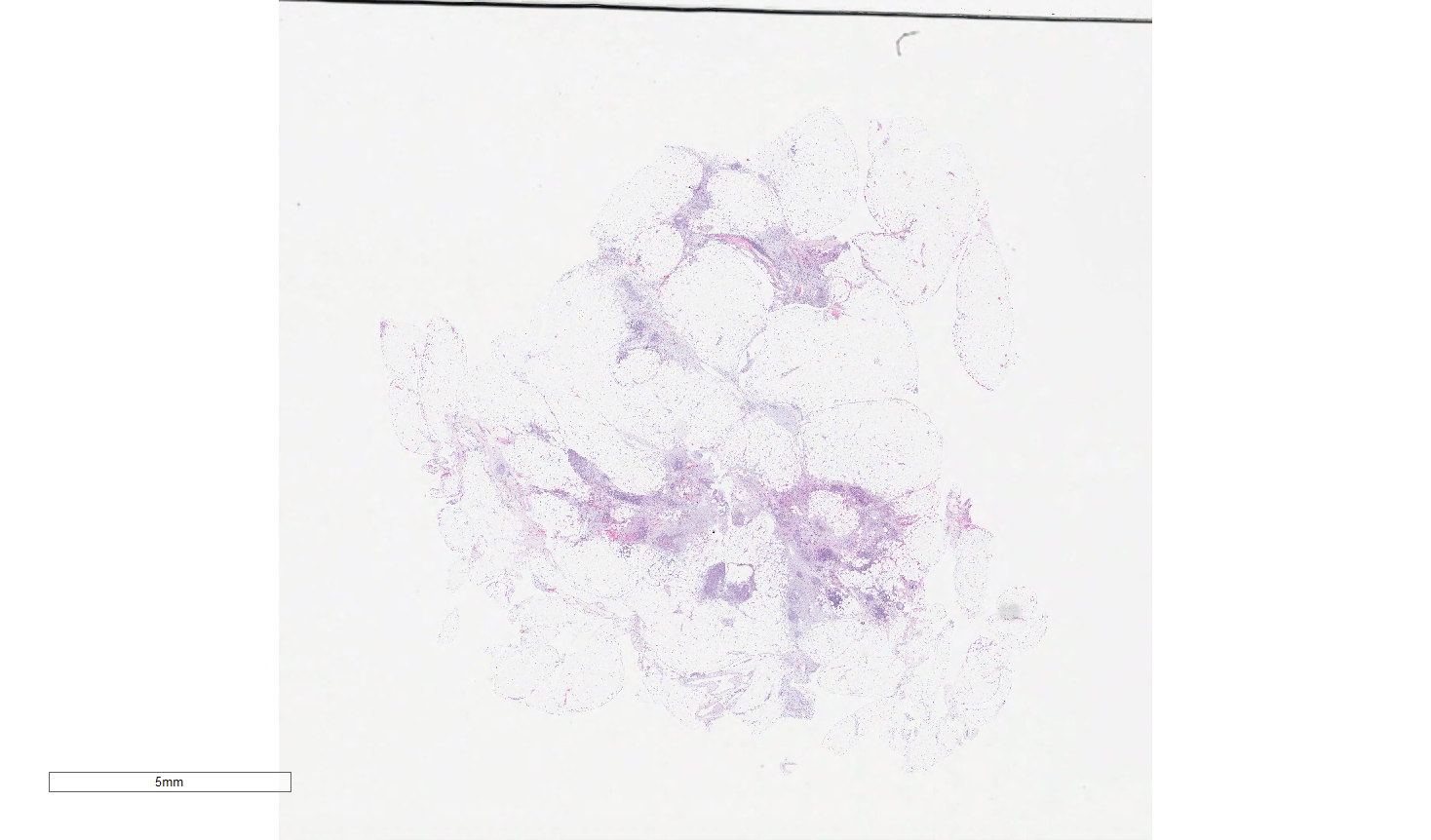

Supplement: Supplementary file 3 — Source Data for Appendix [file EMMM-14-e15729-s005.zip › 600/patient JB257 - H+E whole.tif]

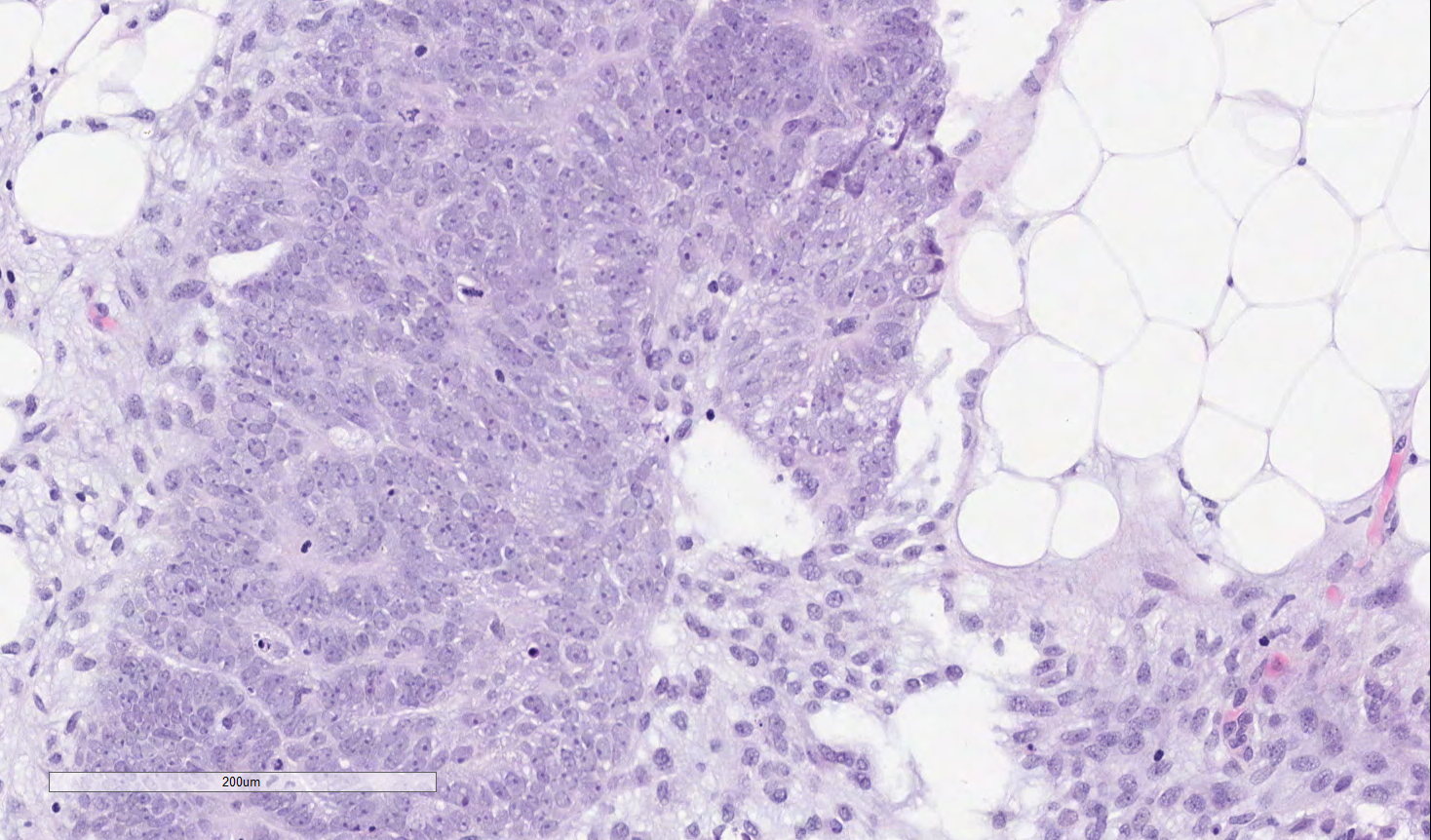

Supplement: Supplementary file 3 — Source Data for Appendix [file EMMM-14-e15729-s005.zip › 600/patient JB257 - H+E zoom.tif]

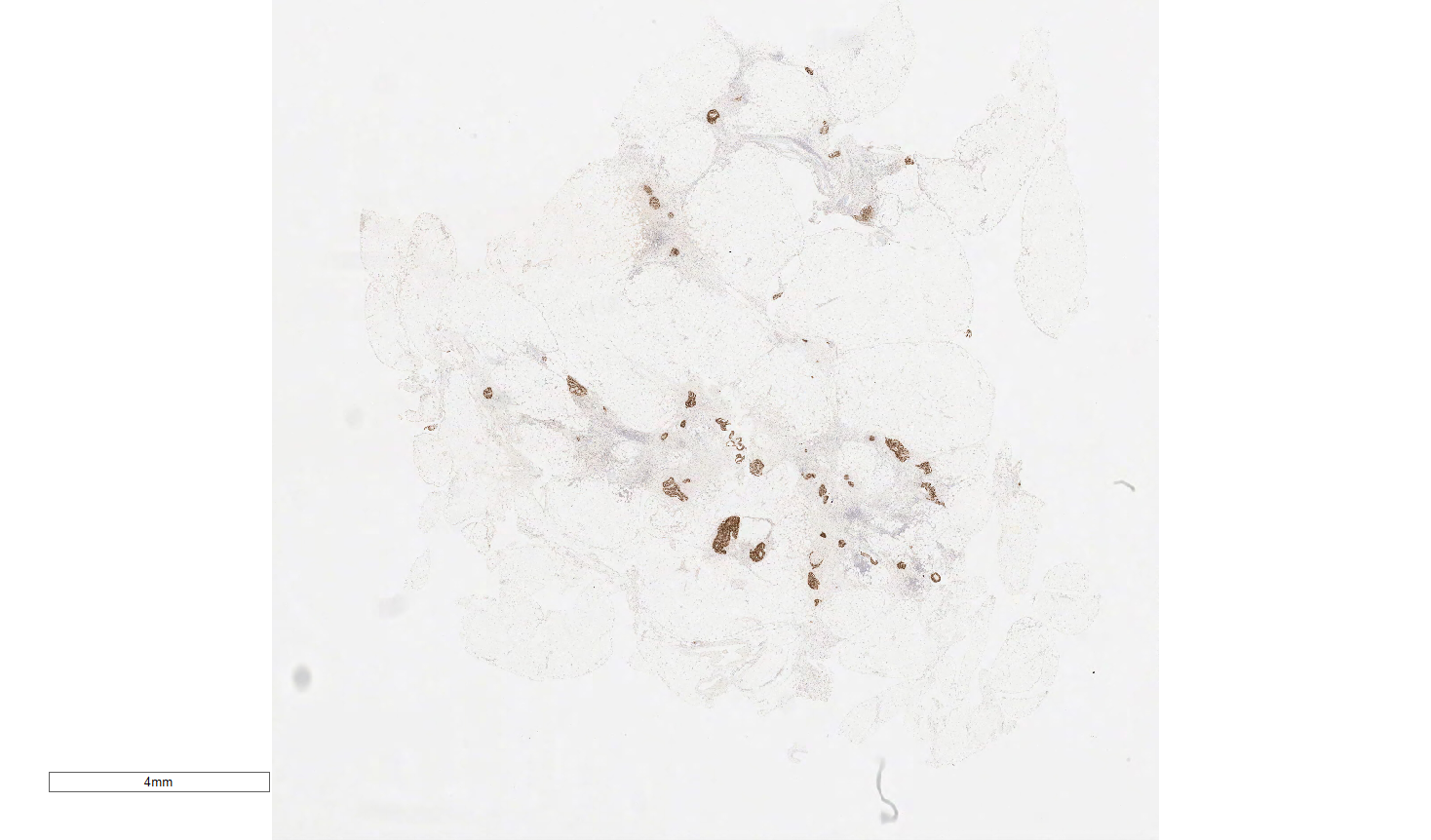

Supplement: Supplementary file 3 — Source Data for Appendix [file EMMM-14-e15729-s005.zip › 600/patient JB257 - p53 whole.tif]

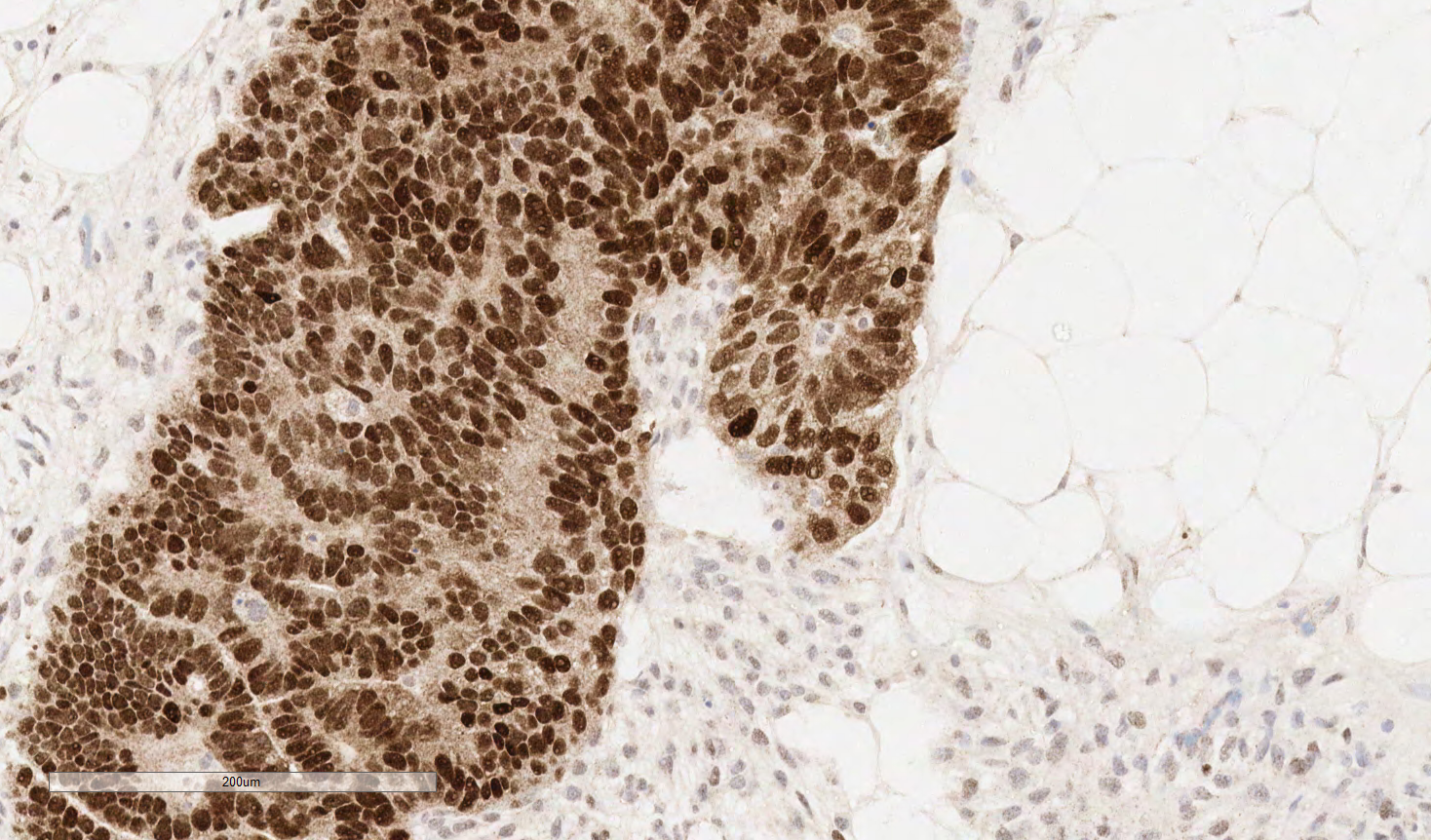

Supplement: Supplementary file 3 — Source Data for Appendix [file EMMM-14-e15729-s005.zip › 600/patient JB257 - p53 zoom.tif]

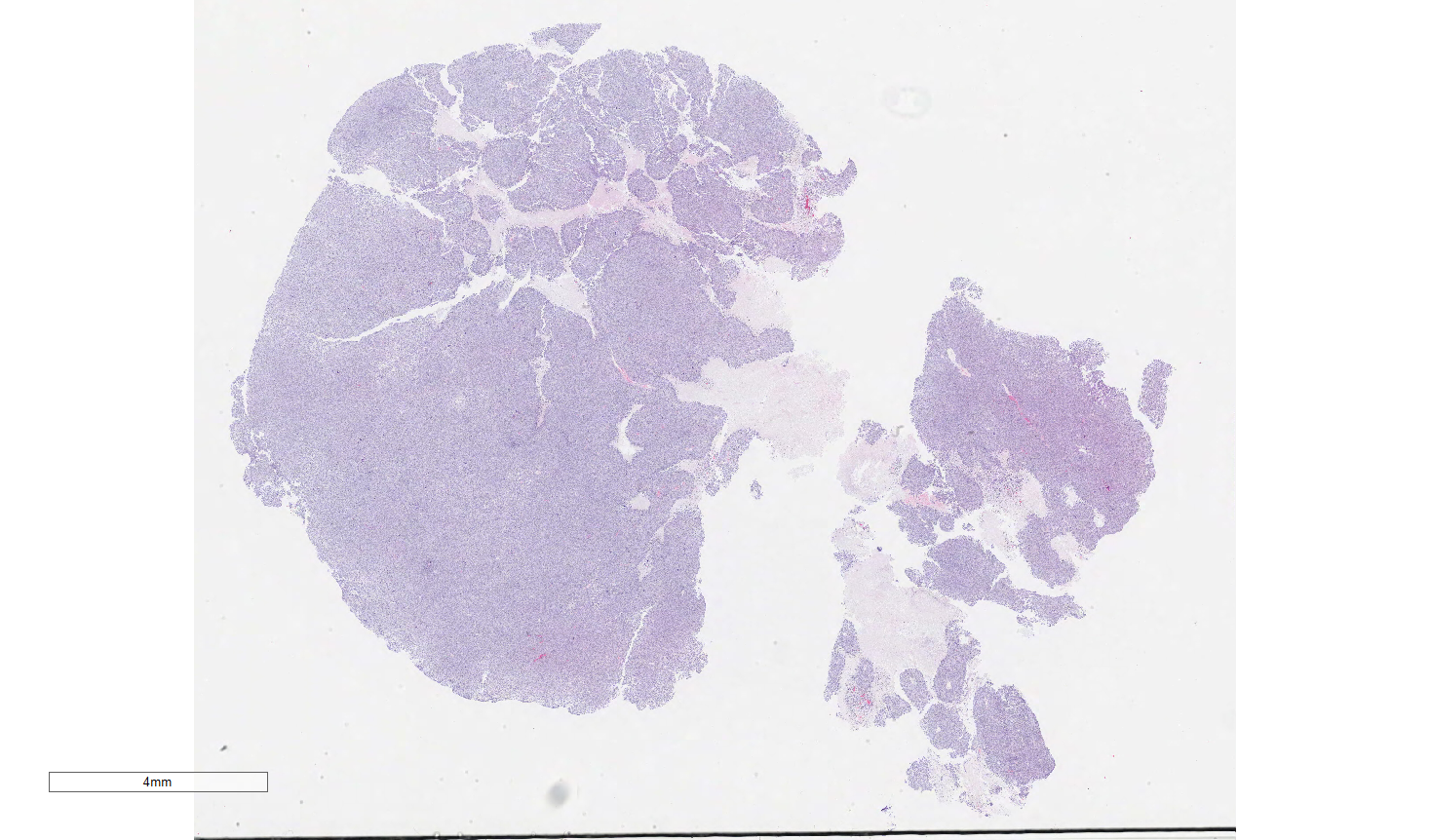

Supplement: Supplementary file 3 — Source Data for Appendix [file EMMM-14-e15729-s005.zip › 771/Mouse 82 JB326 - H+E whole.tif]

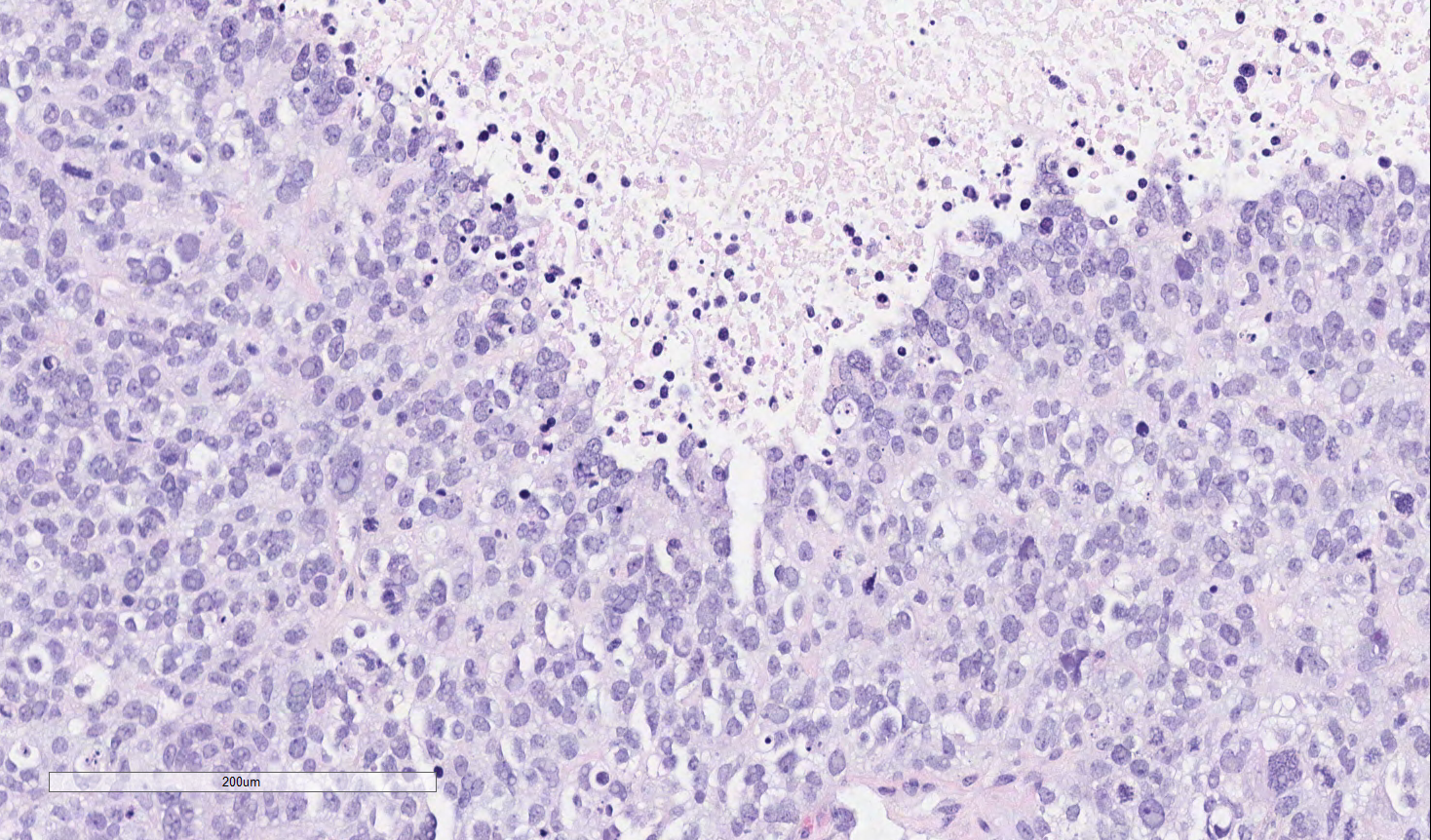

Supplement: Supplementary file 3 — Source Data for Appendix [file EMMM-14-e15729-s005.zip › 771/Mouse 82 JB326 - H+E zoom.tif]

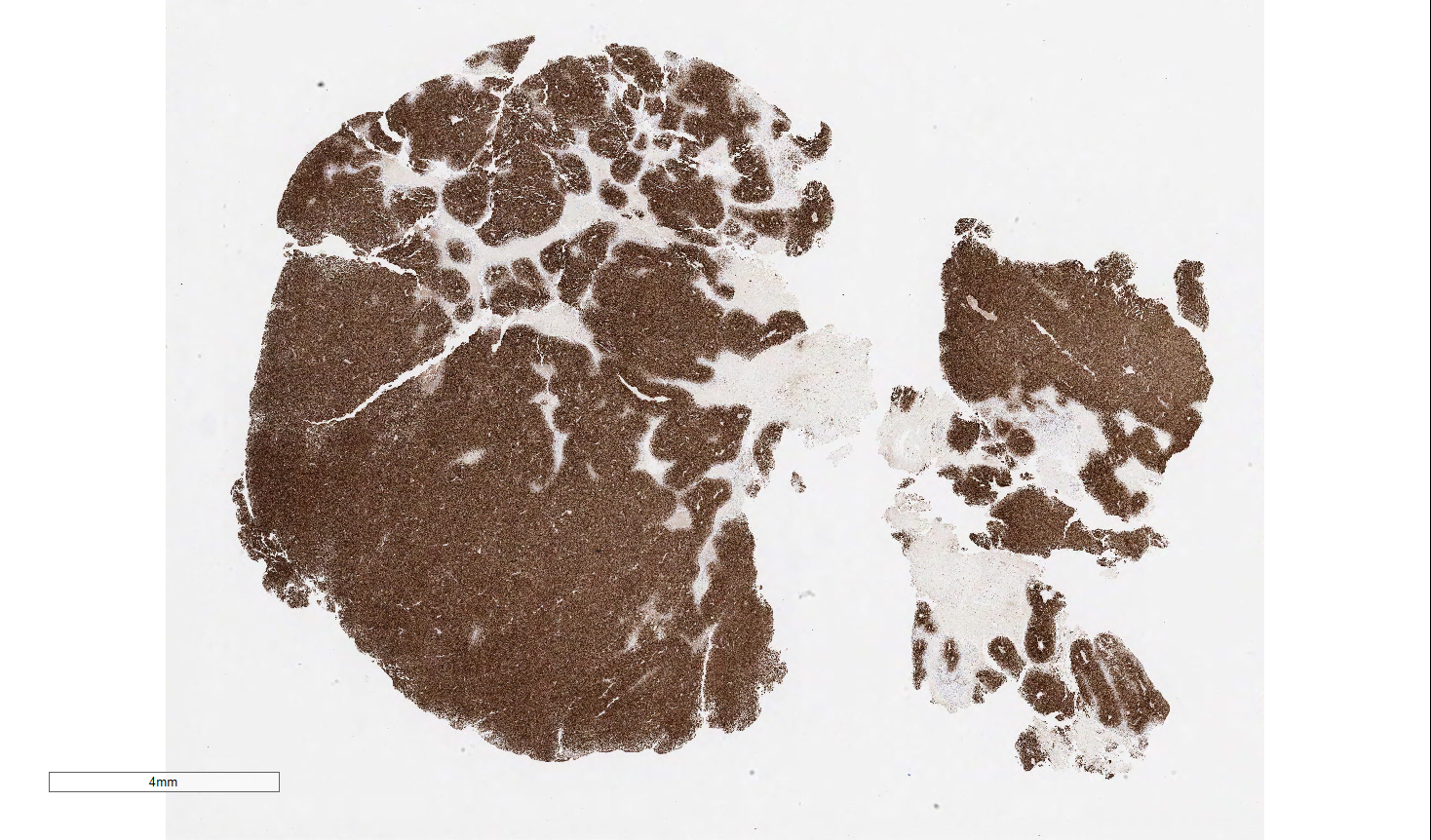

Supplement: Supplementary file 3 — Source Data for Appendix [file EMMM-14-e15729-s005.zip › 771/Mouse 82 JB326 - p53 whole.tif]

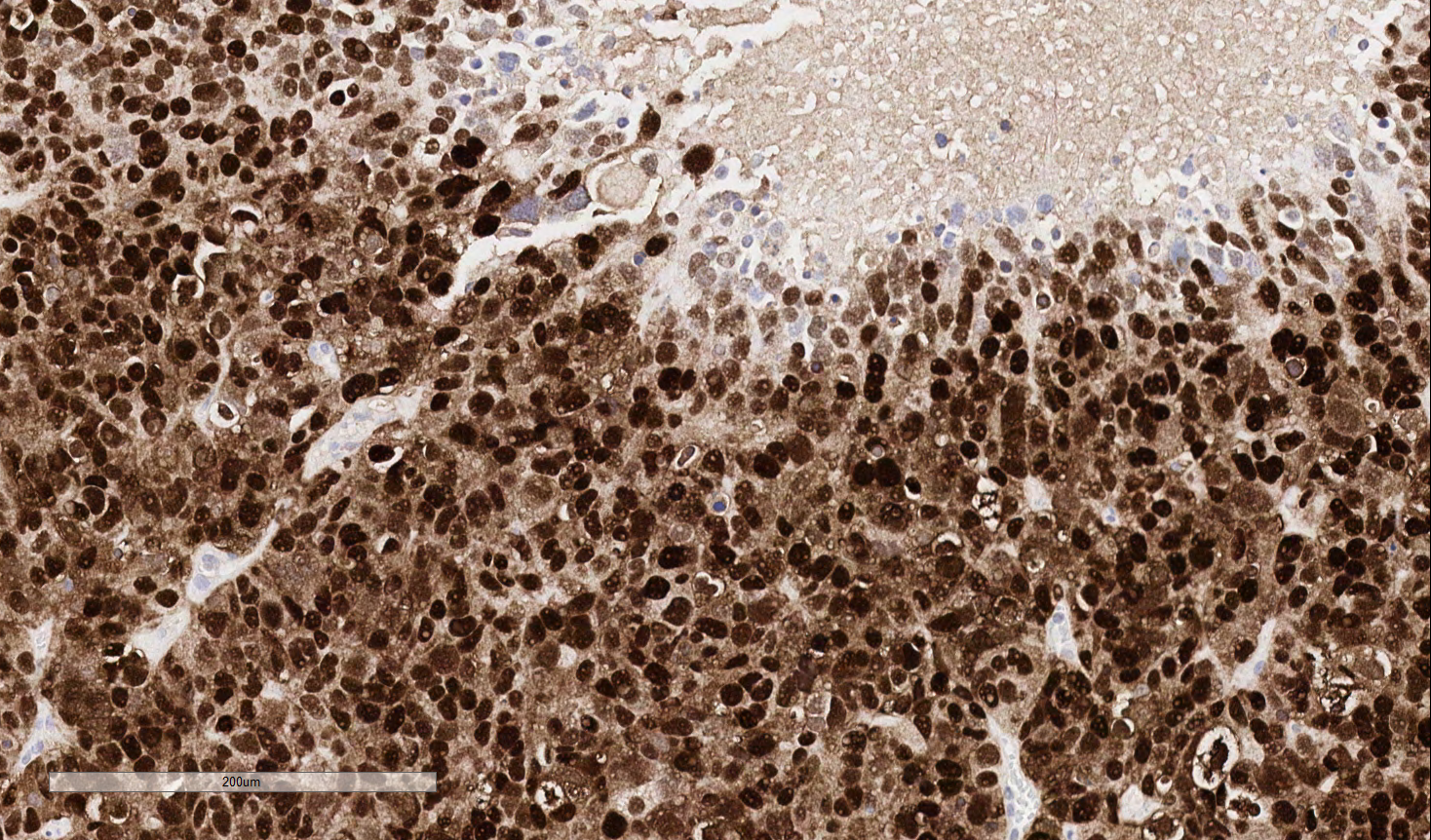

Supplement: Supplementary file 3 — Source Data for Appendix [file EMMM-14-e15729-s005.zip › 771/Mouse 82 JB326 - p53 zoom.tif]

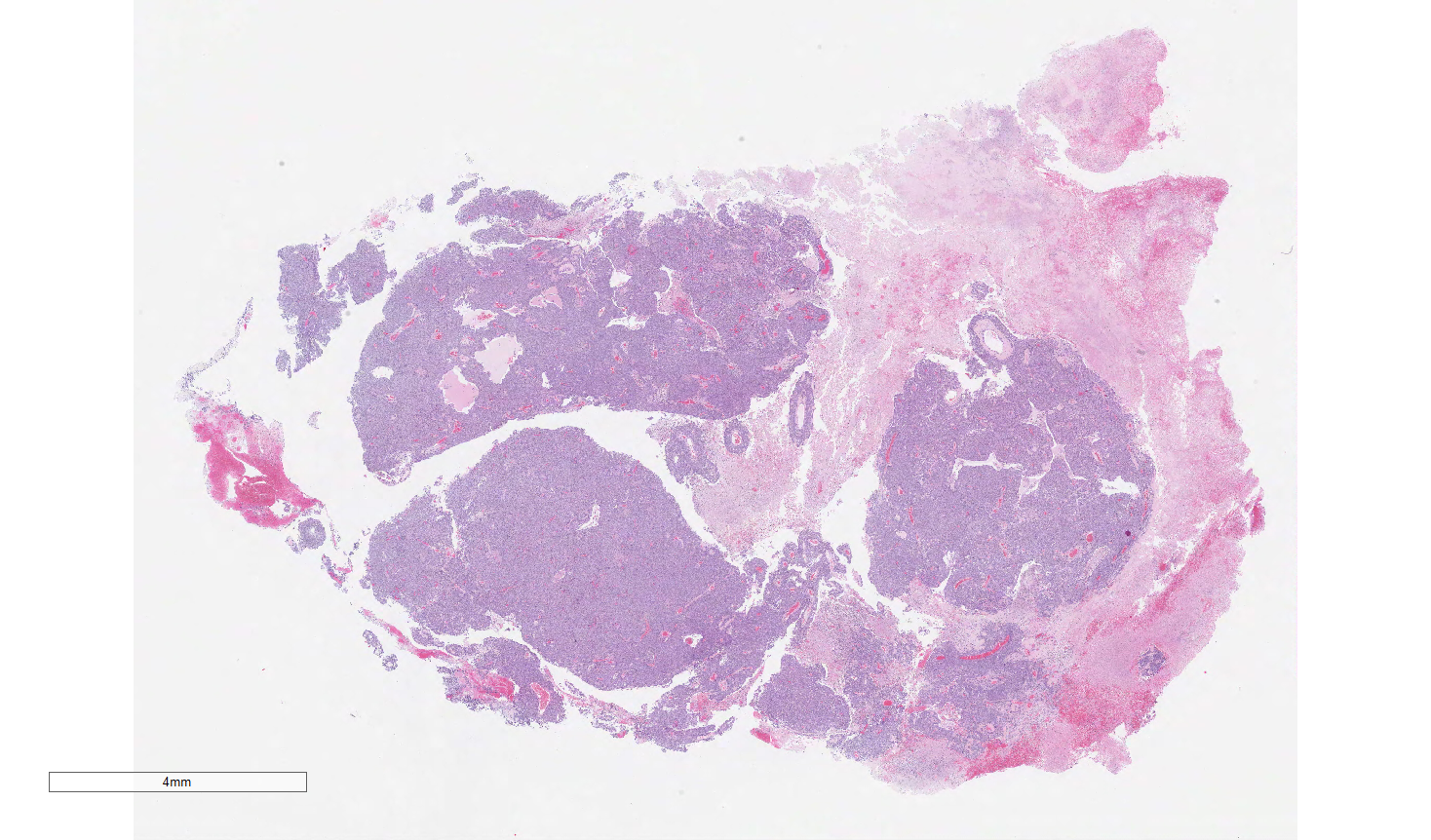

Supplement: Supplementary file 3 — Source Data for Appendix [file EMMM-14-e15729-s005.zip › 771/patient JB326 - H+E whole.tif]

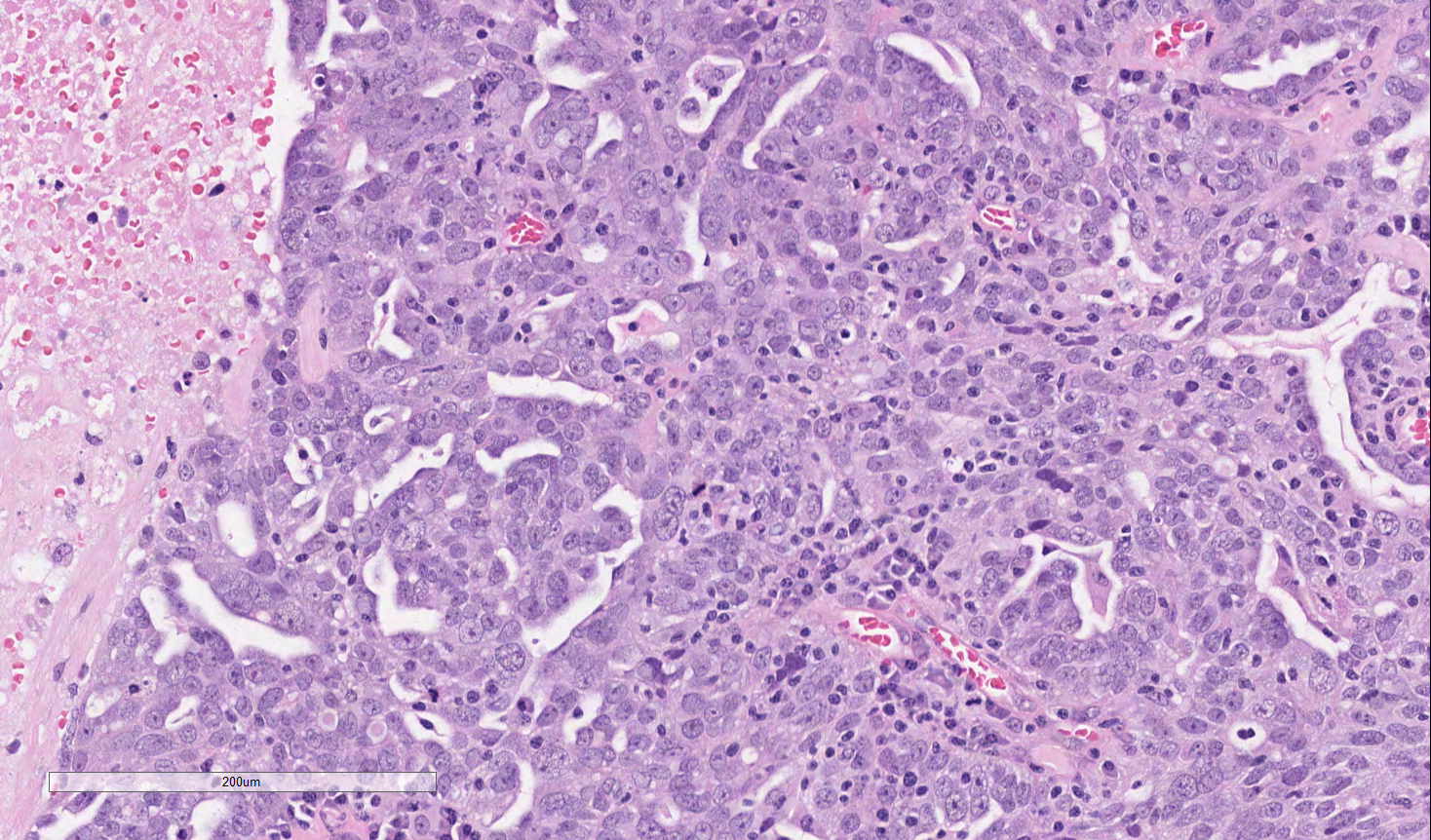

Supplement: Supplementary file 3 — Source Data for Appendix [file EMMM-14-e15729-s005.zip › 771/patient JB326 - H+E zoom.tif]

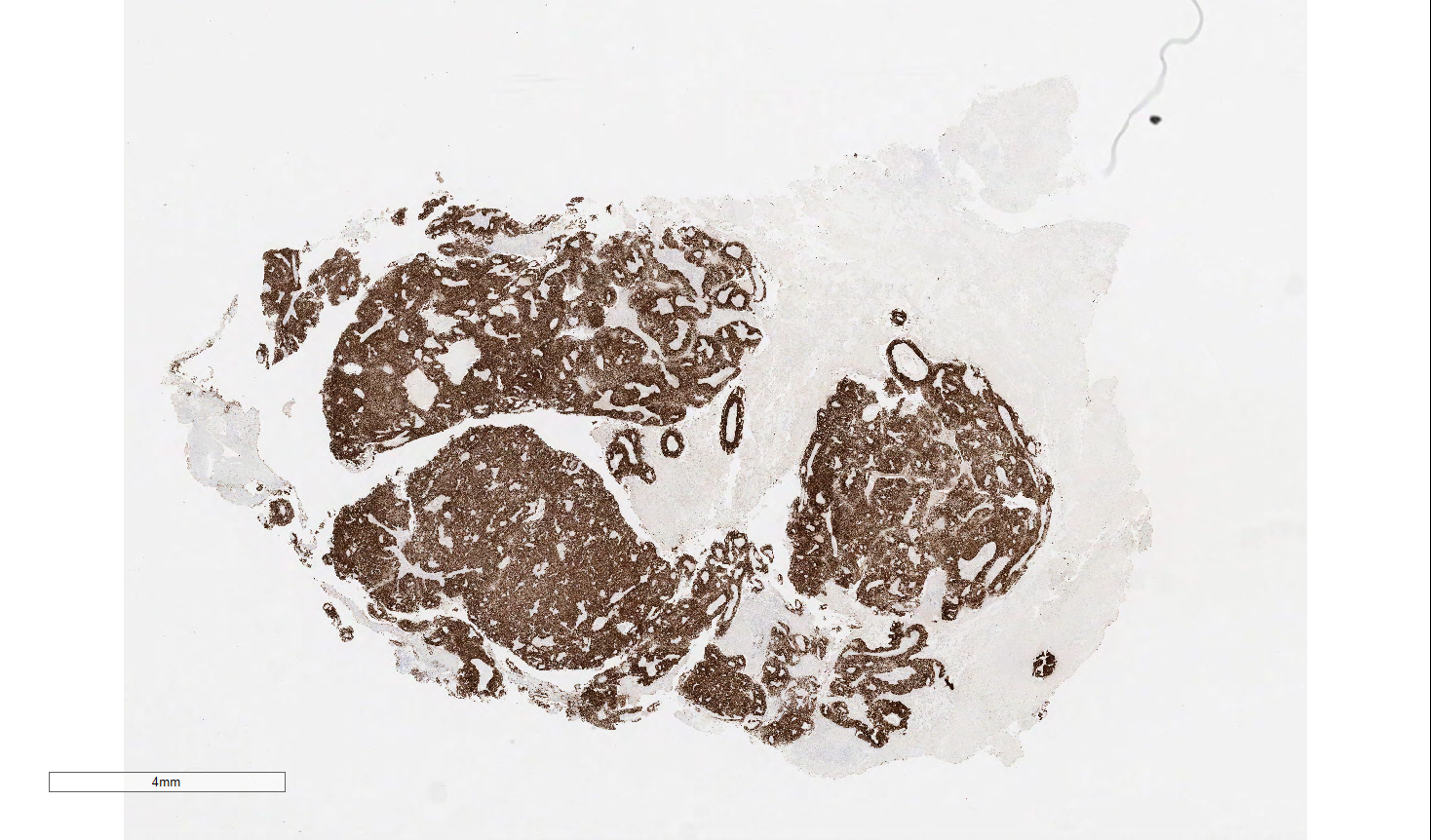

Supplement: Supplementary file 3 — Source Data for Appendix [file EMMM-14-e15729-s005.zip › 771/patient JB326 - p53 whole.tif]

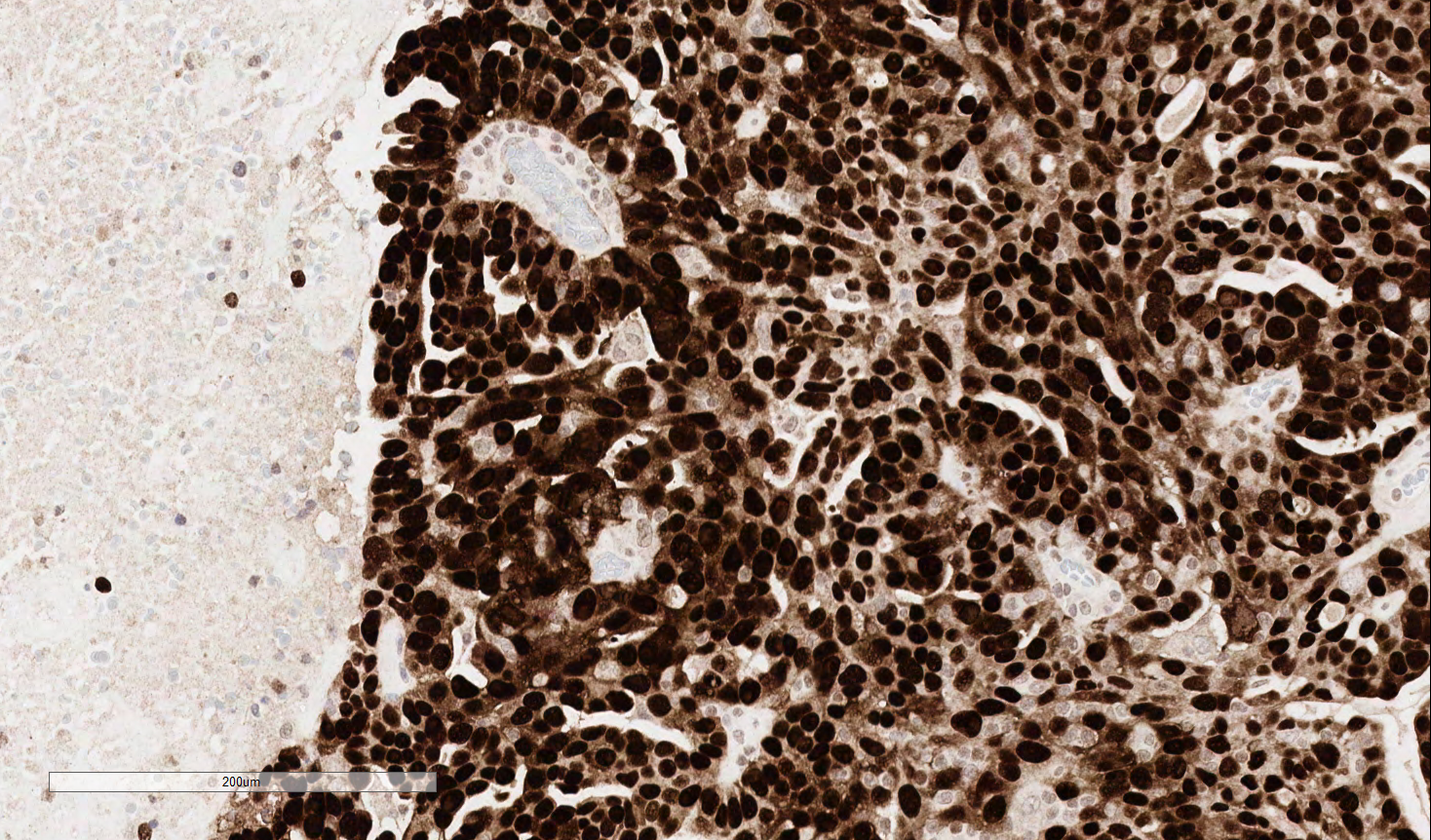

Supplement: Supplementary file 3 — Source Data for Appendix [file EMMM-14-e15729-s005.zip › 771/patient JB326 - p53 zoom.tif]

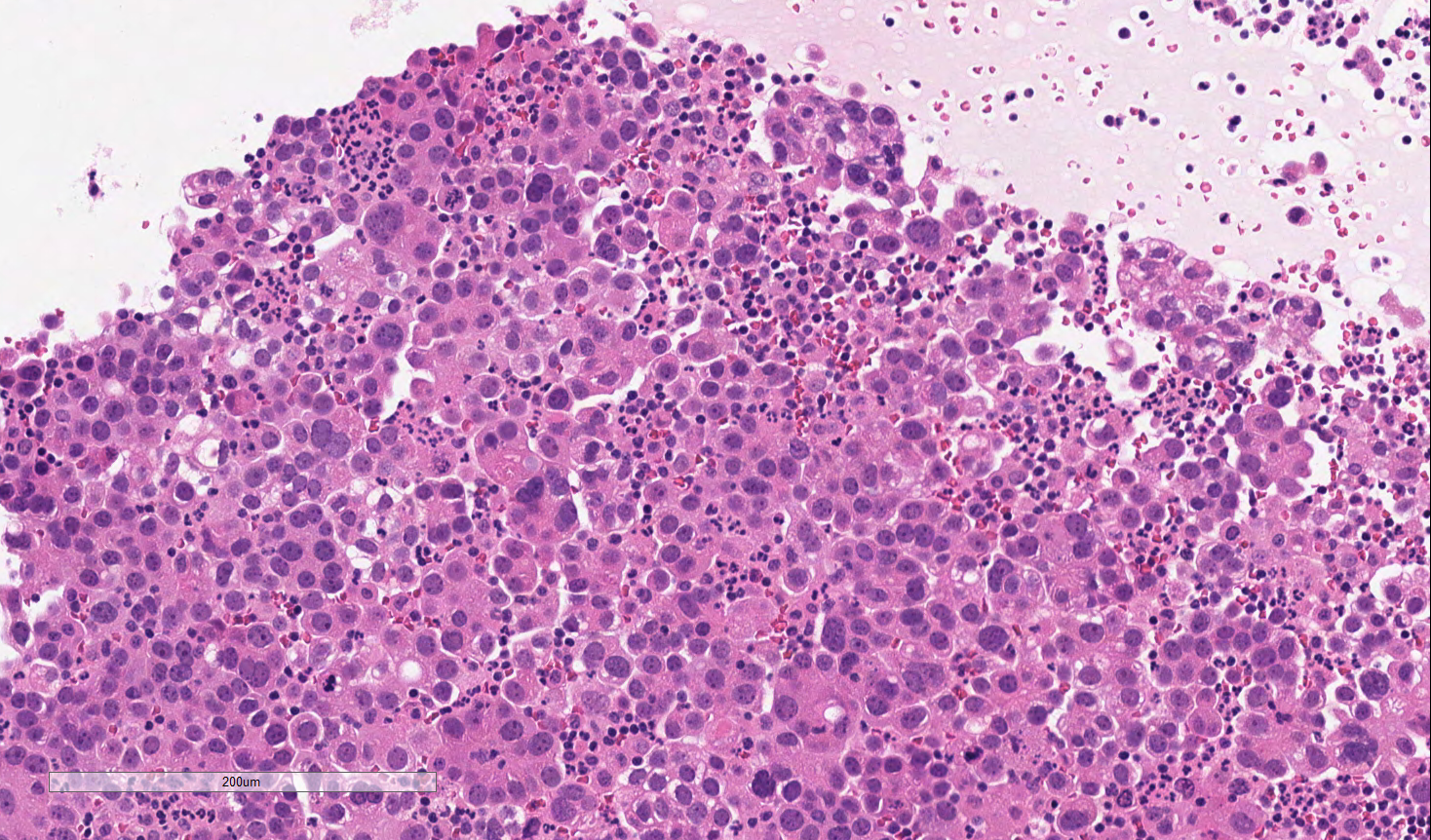

Supplement: Supplementary file 3 — Source Data for Appendix [file EMMM-14-e15729-s005.zip › 828/119011 - H+E zoom.tif]

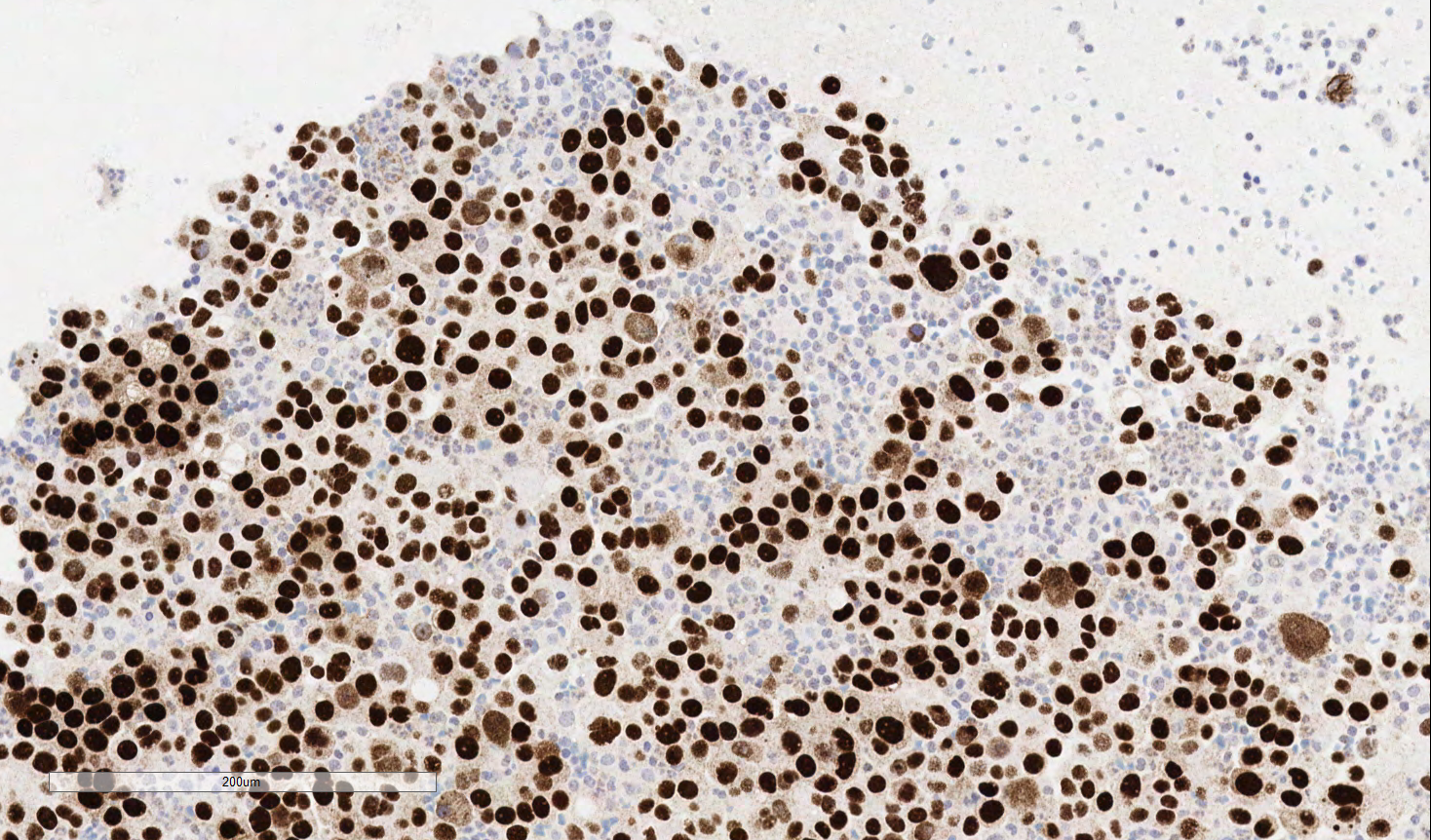

Supplement: Supplementary file 3 — Source Data for Appendix [file EMMM-14-e15729-s005.zip › 828/119011 - p53 zoom.tif]

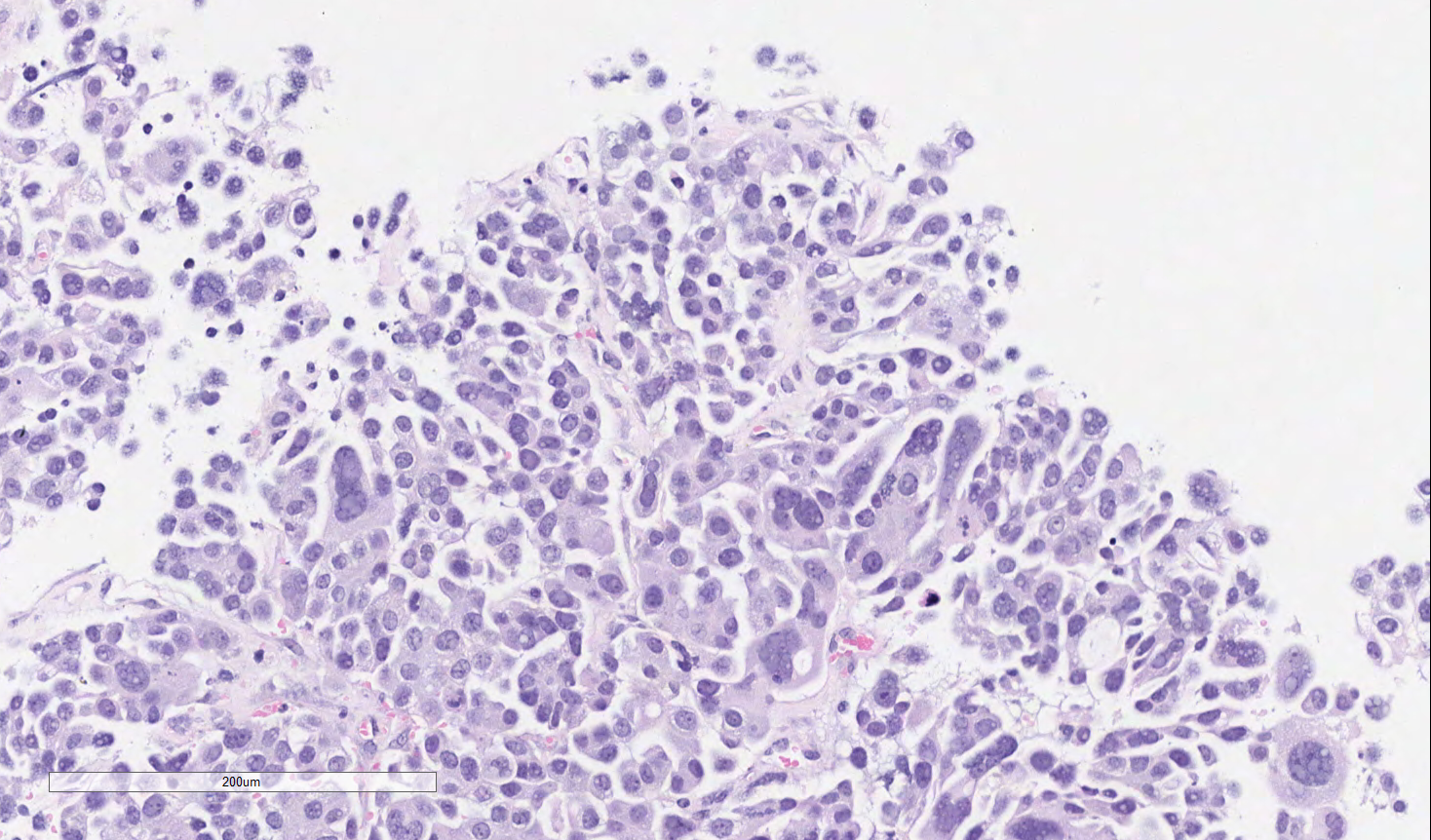

Supplement: Supplementary file 3 — Source Data for Appendix [file EMMM-14-e15729-s005.zip › 828/Mouse 58 119011 - H+E zoom.tif]

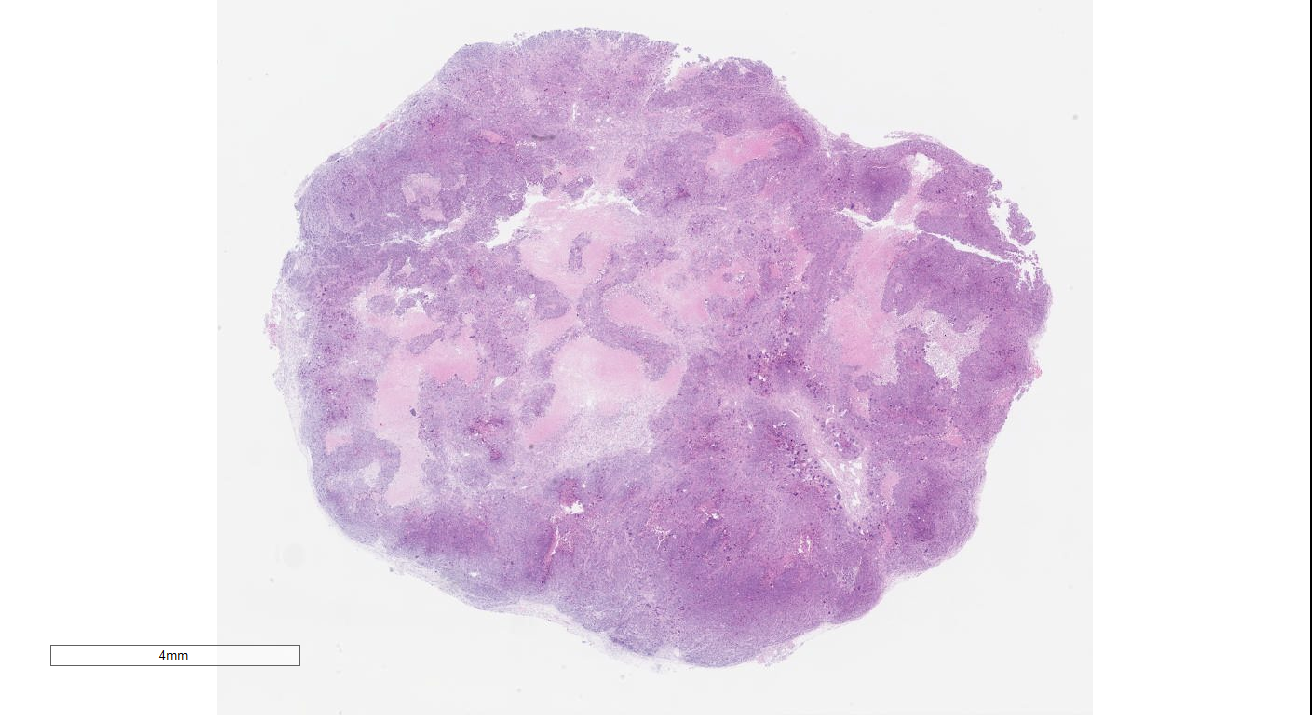

Supplement: Supplementary file 3 — Source Data for Appendix [file EMMM-14-e15729-s005.zip › 828/Mouse 58 119011 - H+E.tif]

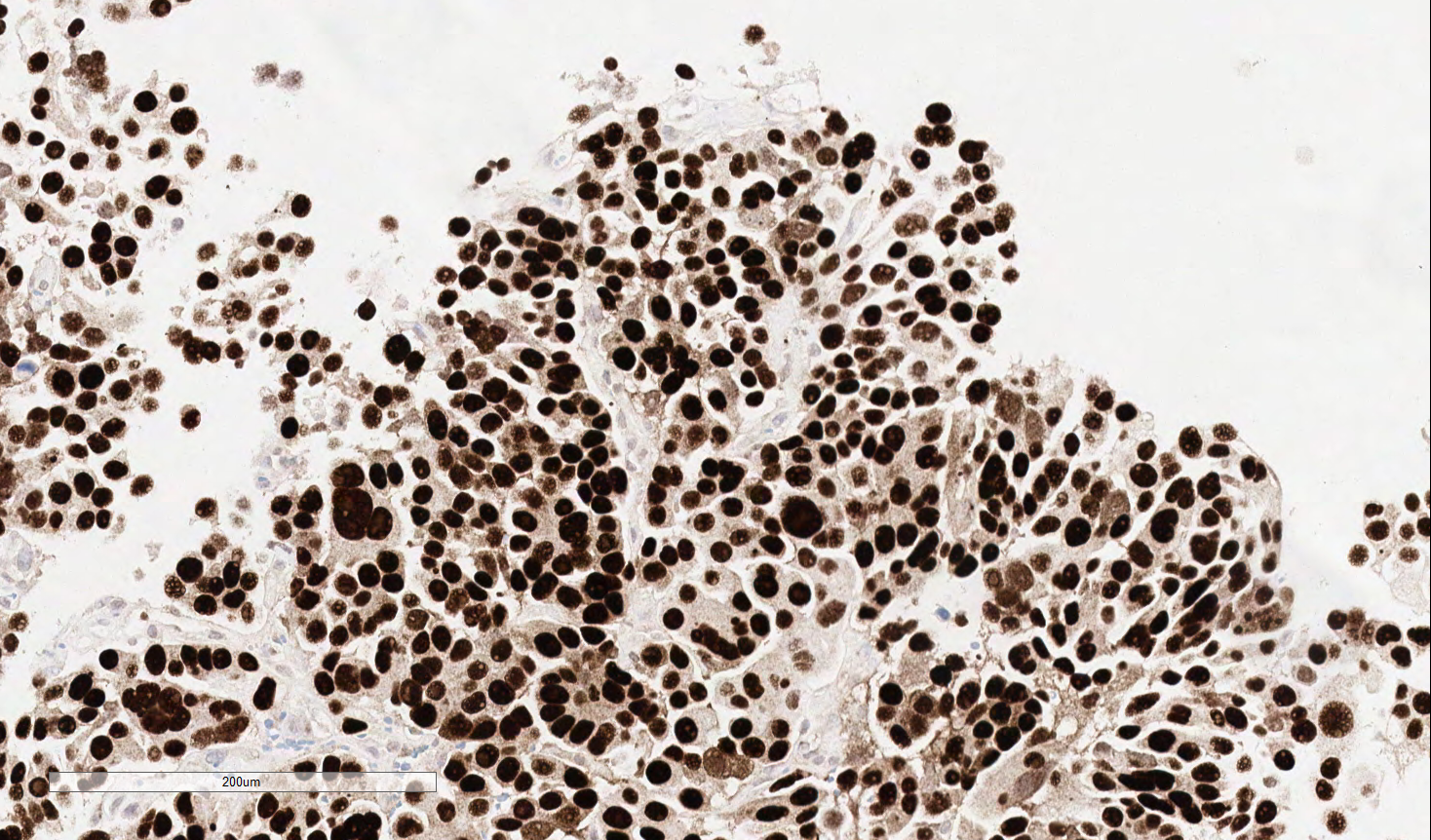

Supplement: Supplementary file 3 — Source Data for Appendix [file EMMM-14-e15729-s005.zip › 828/Mouse 58 119011 - p53 zoom.tif]

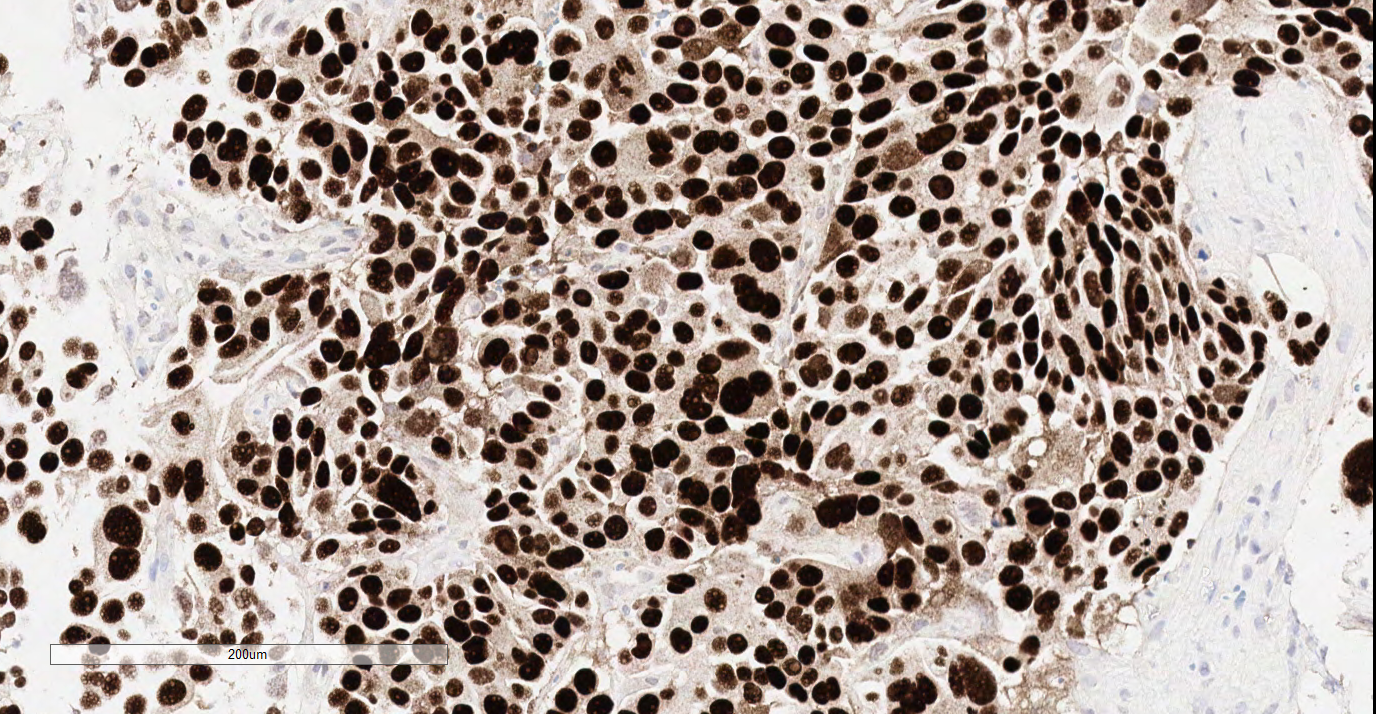

Supplement: Supplementary file 3 — Source Data for Appendix [file EMMM-14-e15729-s005.zip › 828/Mouse 58 119011 - p53.tif]

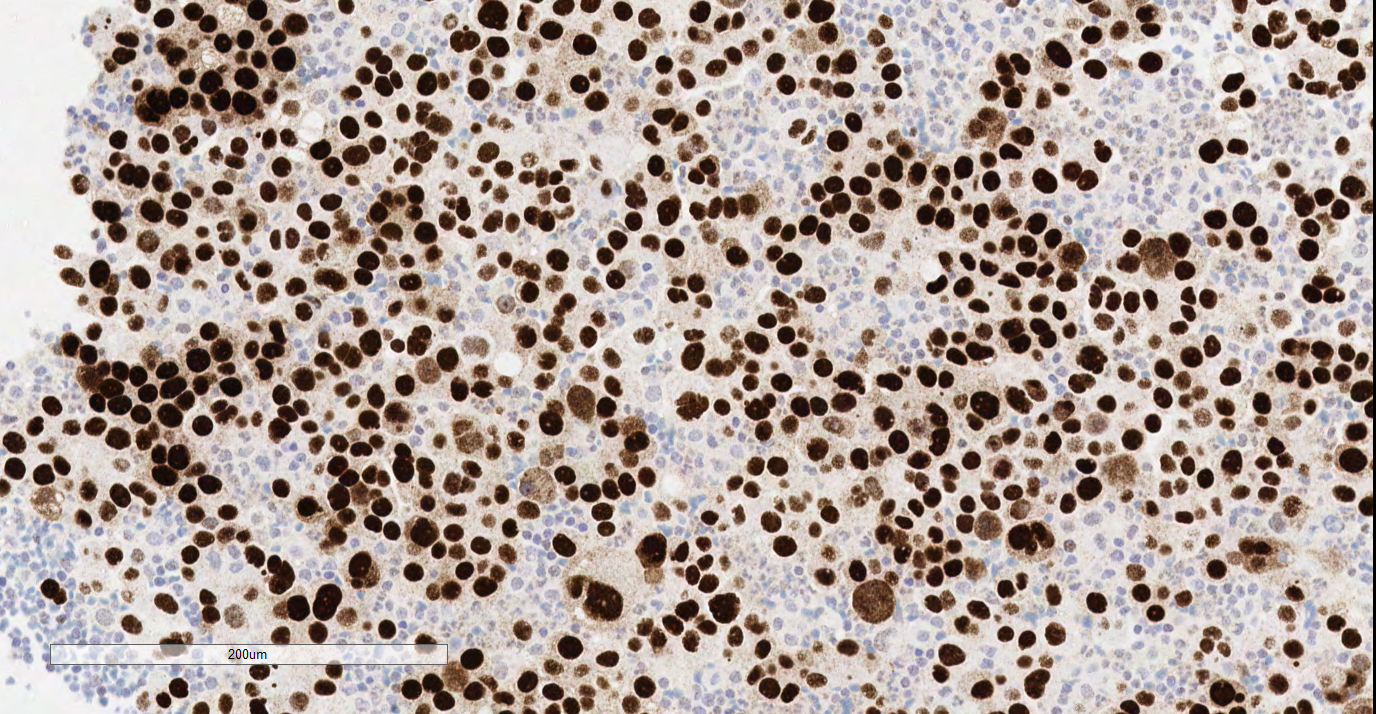

Supplement: Supplementary file 3 — Source Data for Appendix [file EMMM-14-e15729-s005.zip › 828/paitent 119011 - p53.tif]

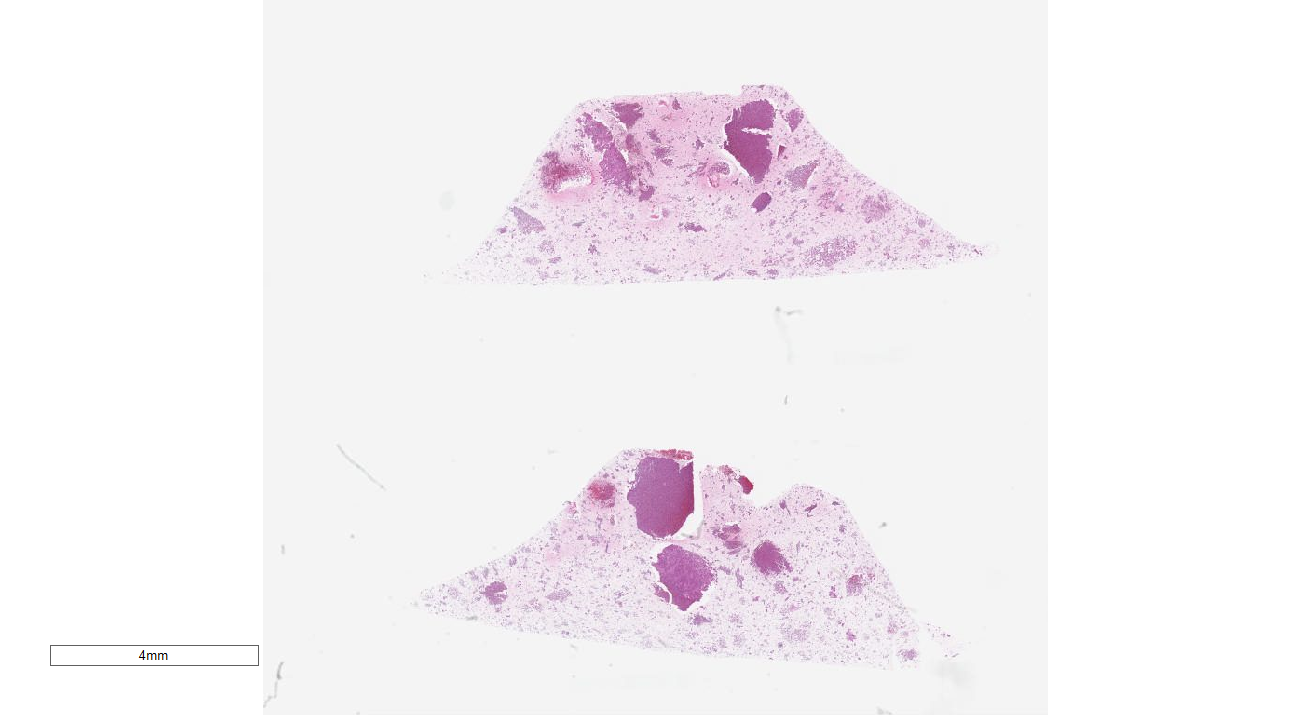

Supplement: Supplementary file 3 — Source Data for Appendix [file EMMM-14-e15729-s005.zip › 828/patient 119011 - H+E whole.tif]

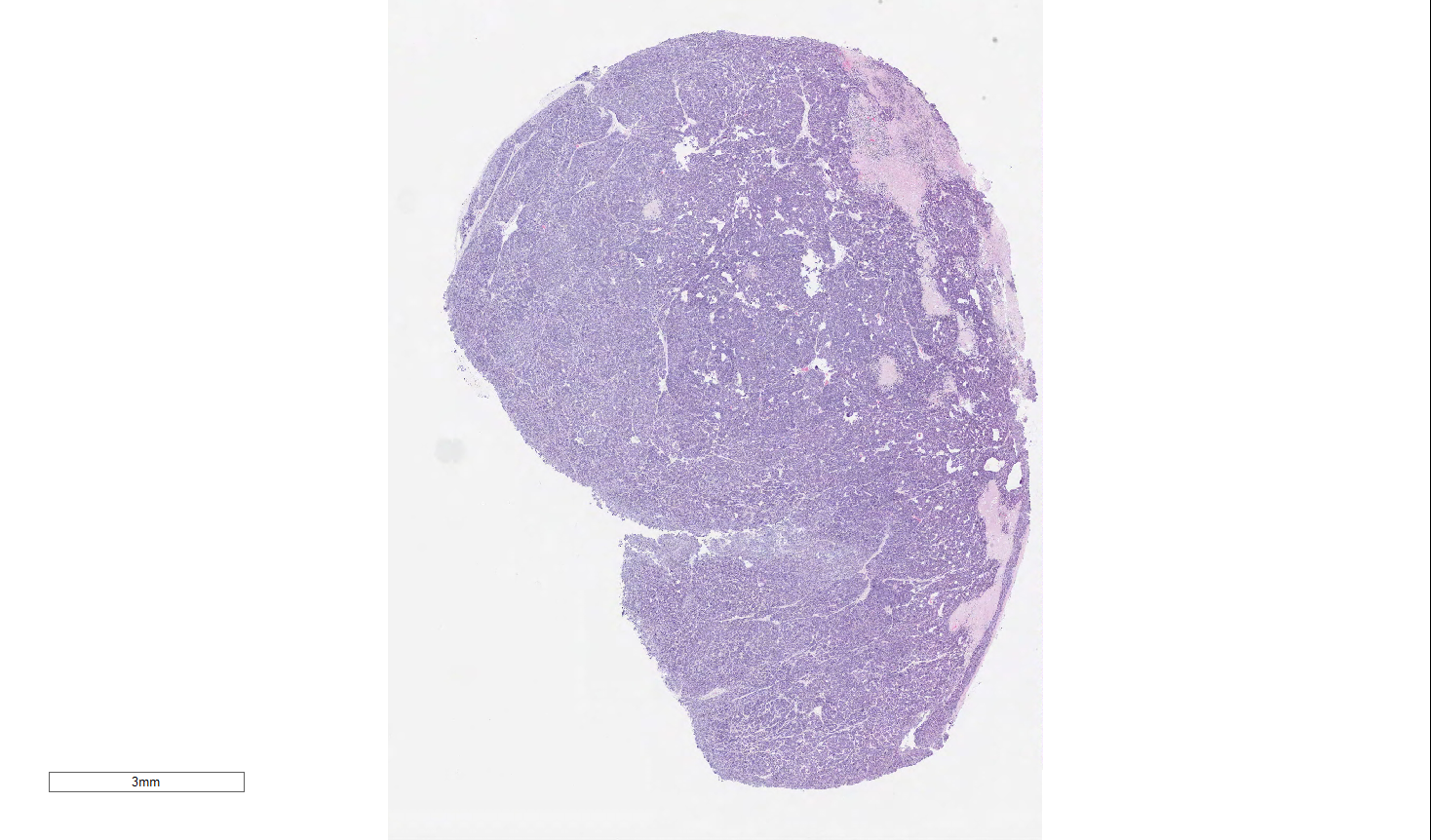

Supplement: Supplementary file 3 — Source Data for Appendix [file EMMM-14-e15729-s005.zip › 831/Mouse 54 JB348 - H+E whole.tif]

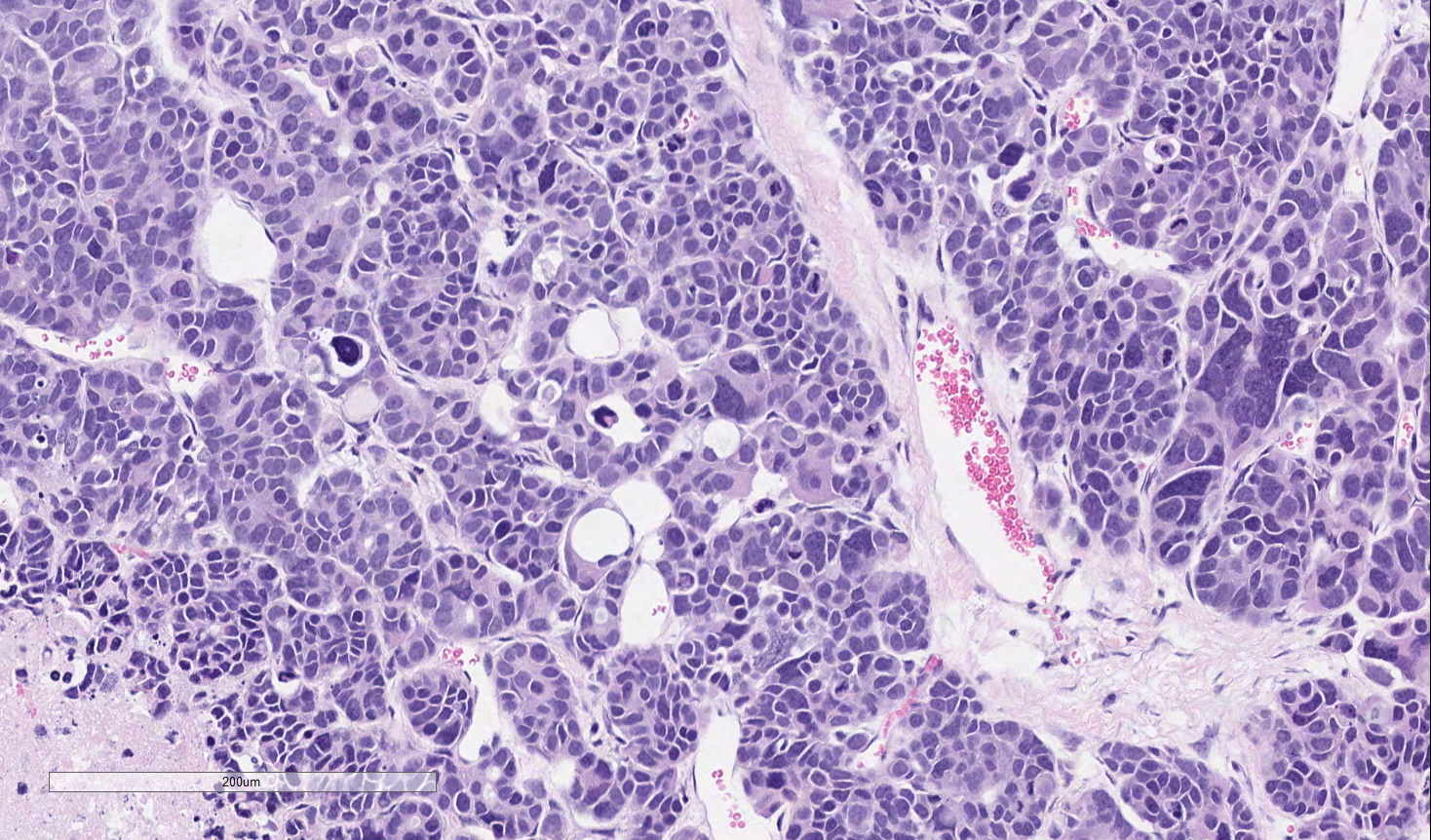

Supplement: Supplementary file 3 — Source Data for Appendix [file EMMM-14-e15729-s005.zip › 831/Mouse 54 JB348 - H+E zoom.tif]

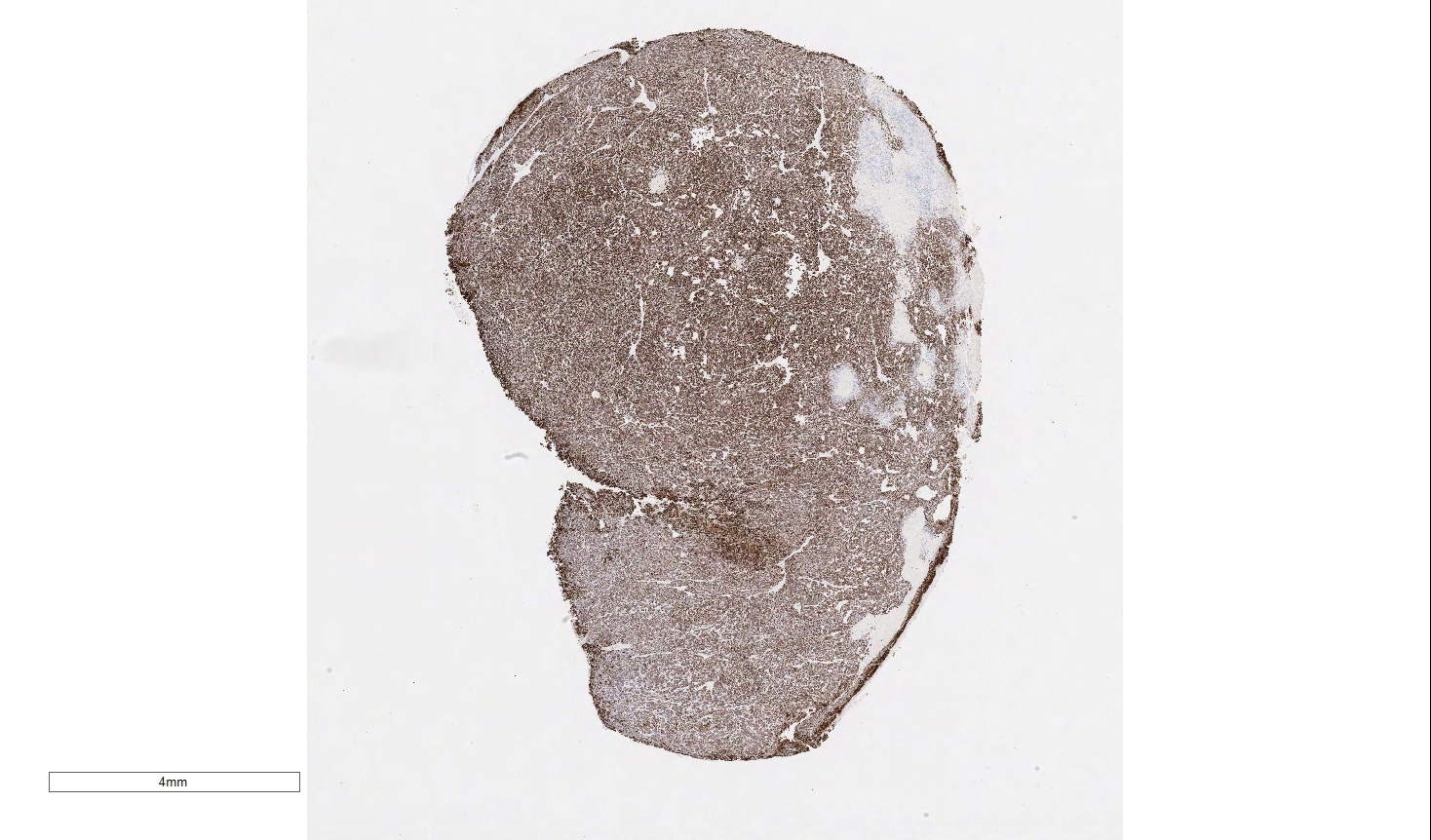

Supplement: Supplementary file 3 — Source Data for Appendix [file EMMM-14-e15729-s005.zip › 831/Mouse 54 JB348 - p53 whole.tif]

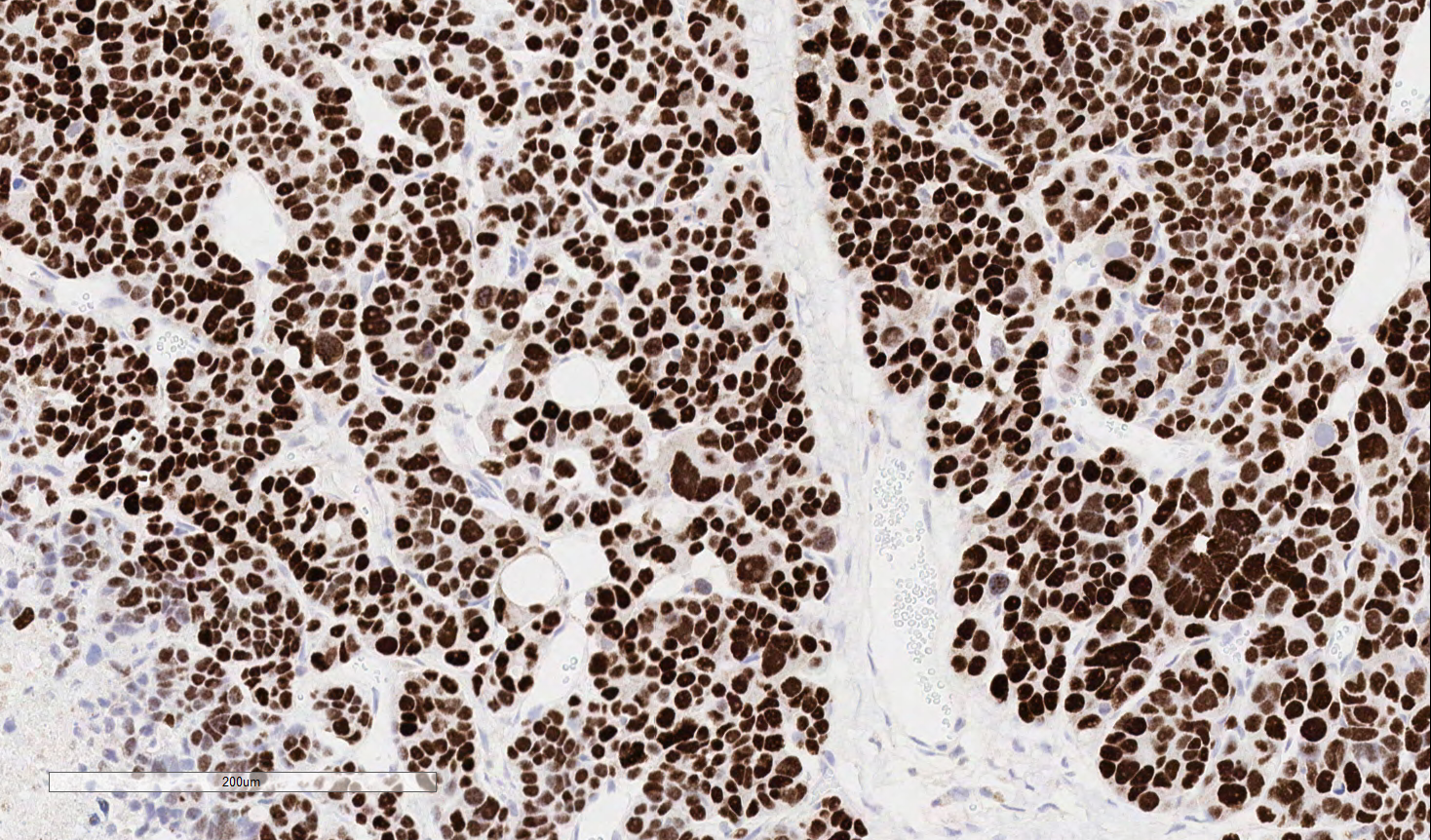

Supplement: Supplementary file 3 — Source Data for Appendix [file EMMM-14-e15729-s005.zip › 831/Mouse 54 JB348 - p53 zoom.tif]

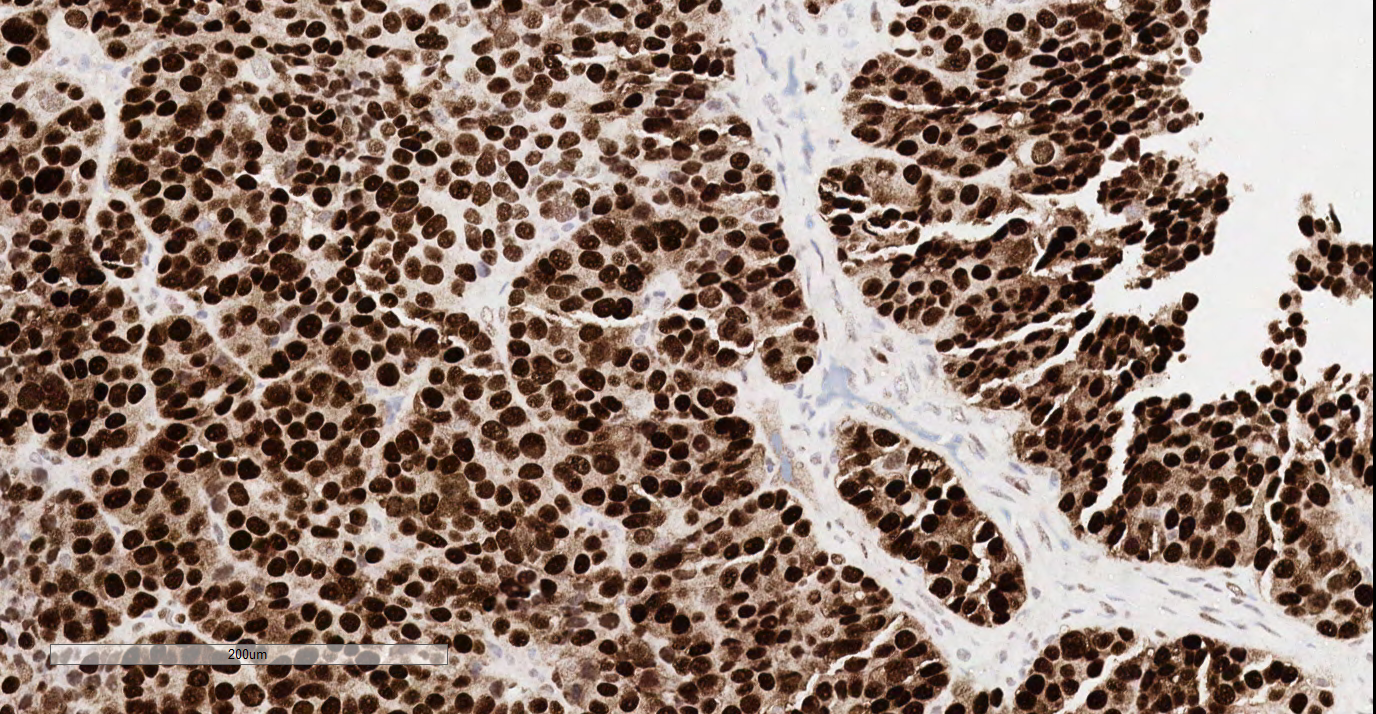

Supplement: Supplementary file 3 — Source Data for Appendix [file EMMM-14-e15729-s005.zip › 831/paitent JB348 - p53.tif]

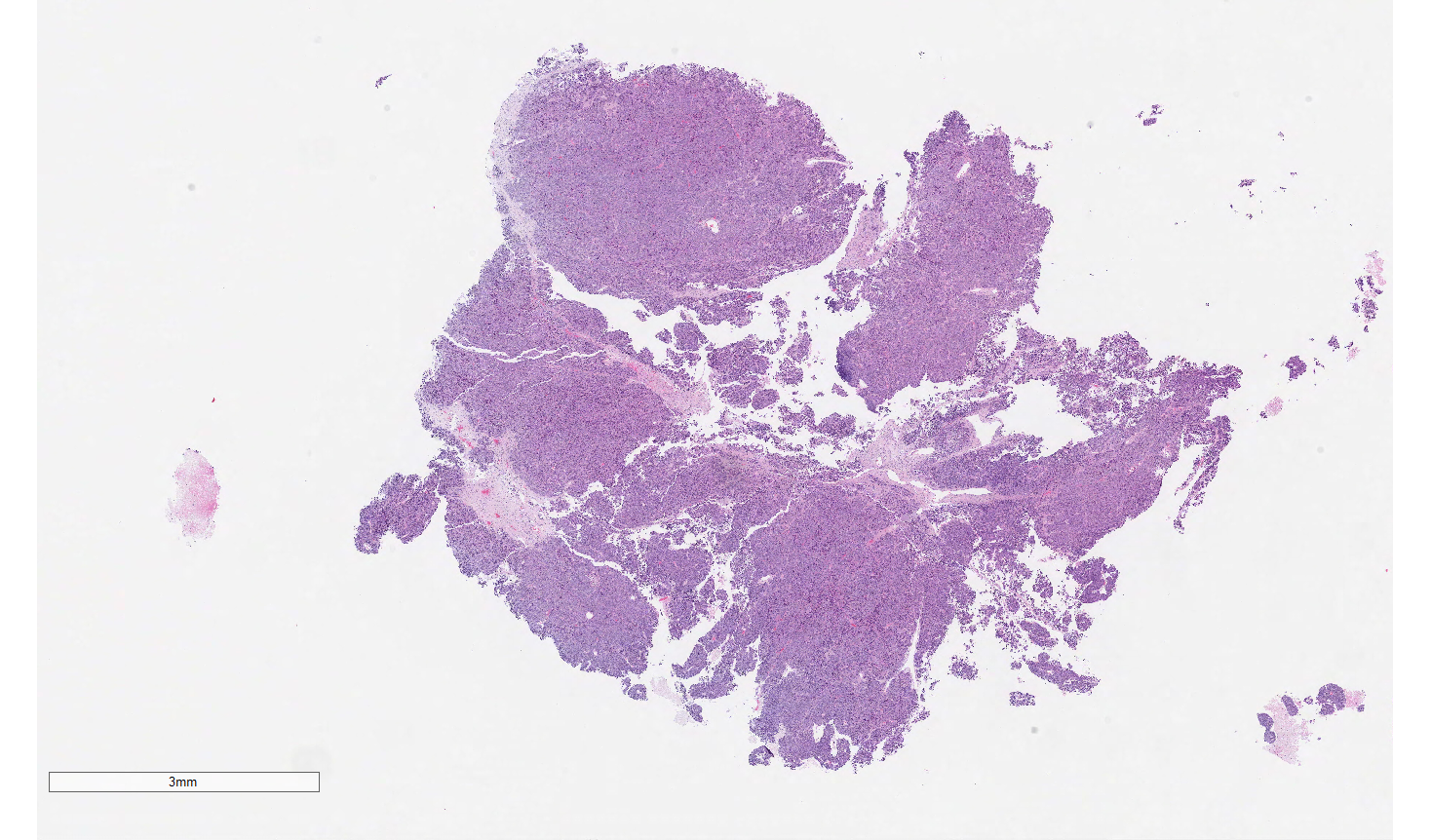

Supplement: Supplementary file 3 — Source Data for Appendix [file EMMM-14-e15729-s005.zip › 831/patient JB348 - H+E whole.tif]

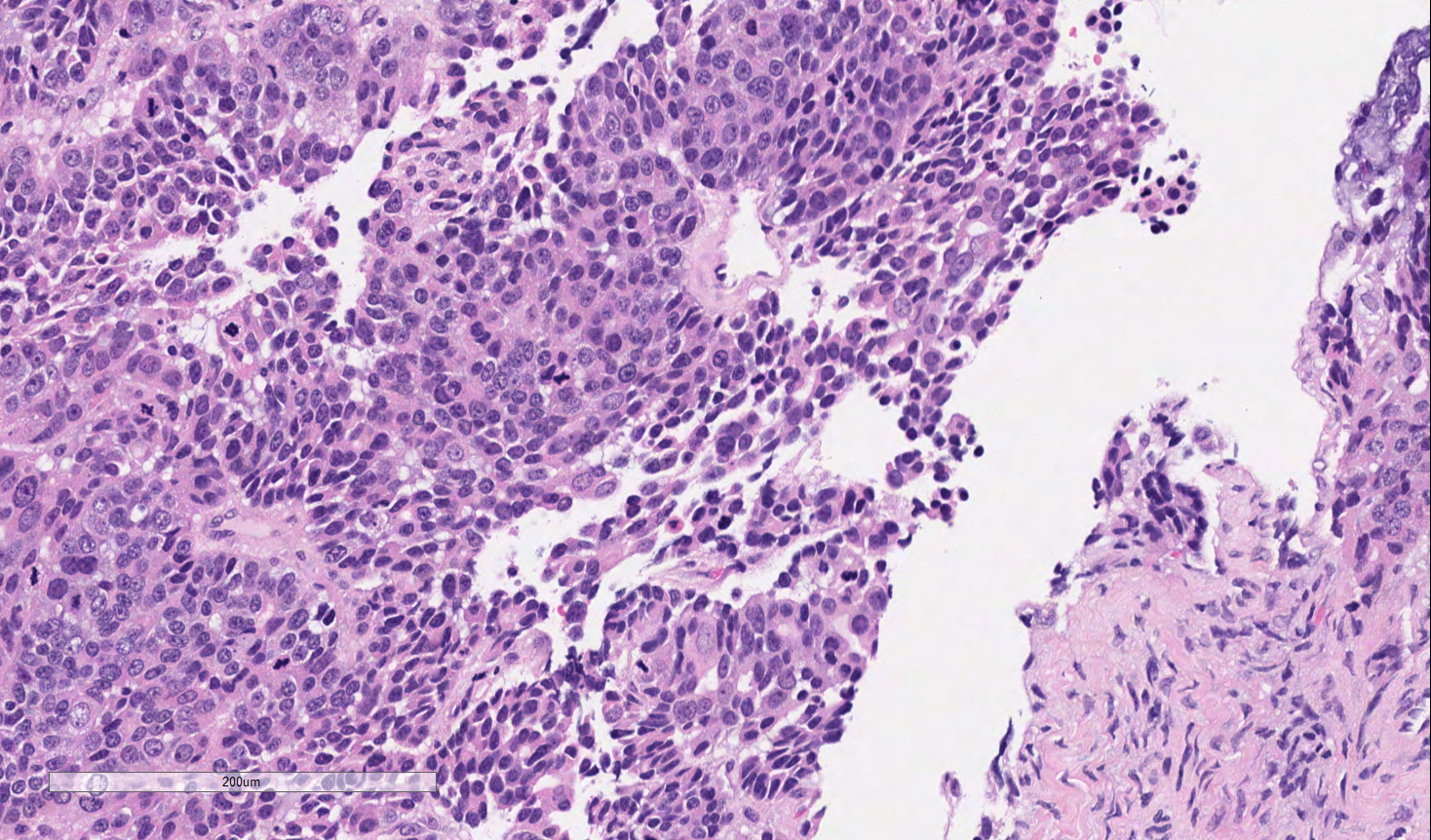

Supplement: Supplementary file 3 — Source Data for Appendix [file EMMM-14-e15729-s005.zip › 831/patient JB348 - H+E zoom.tif]

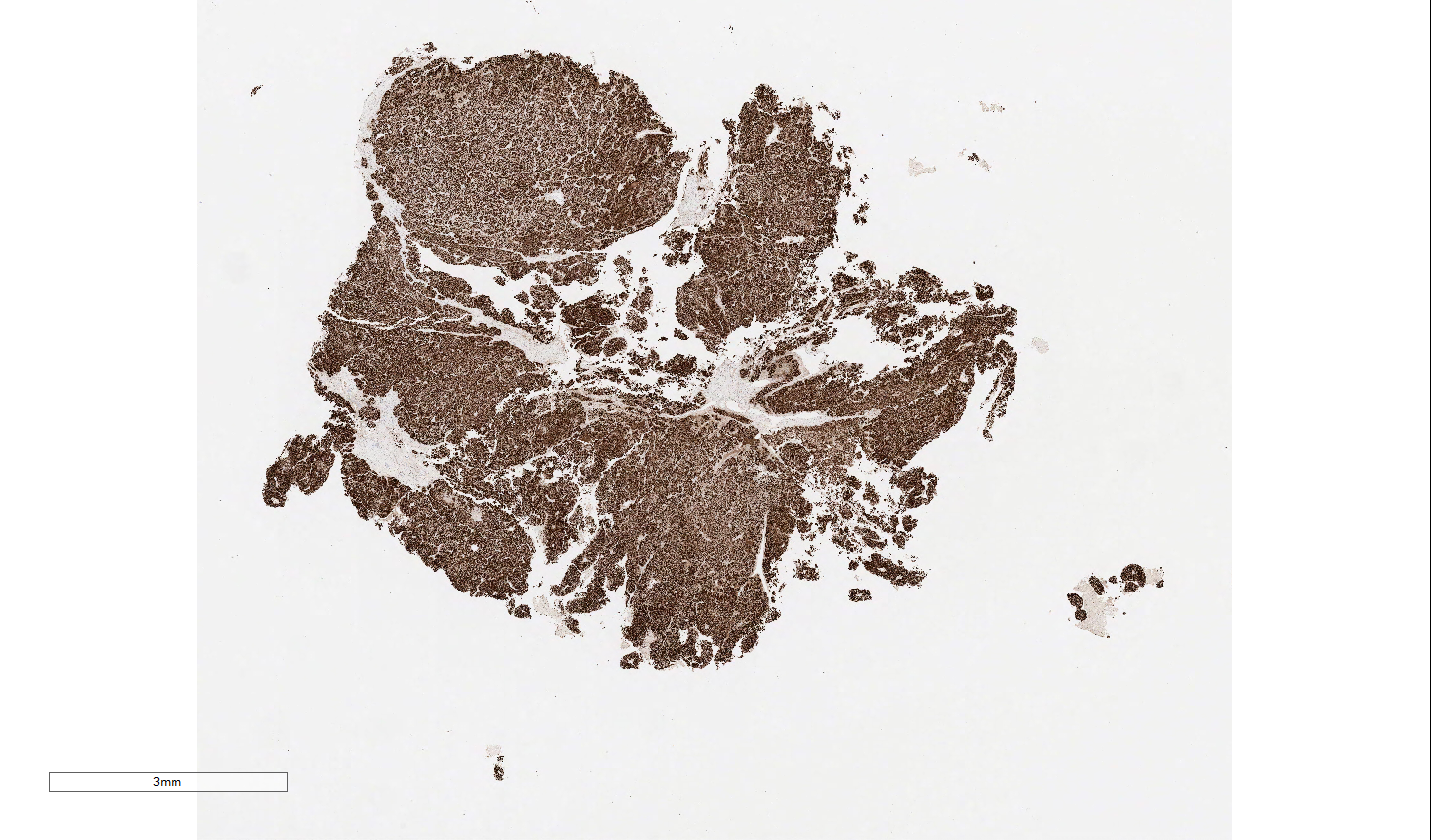

Supplement: Supplementary file 3 — Source Data for Appendix [file EMMM-14-e15729-s005.zip › 831/patient JB348 - p53 whole.tif]

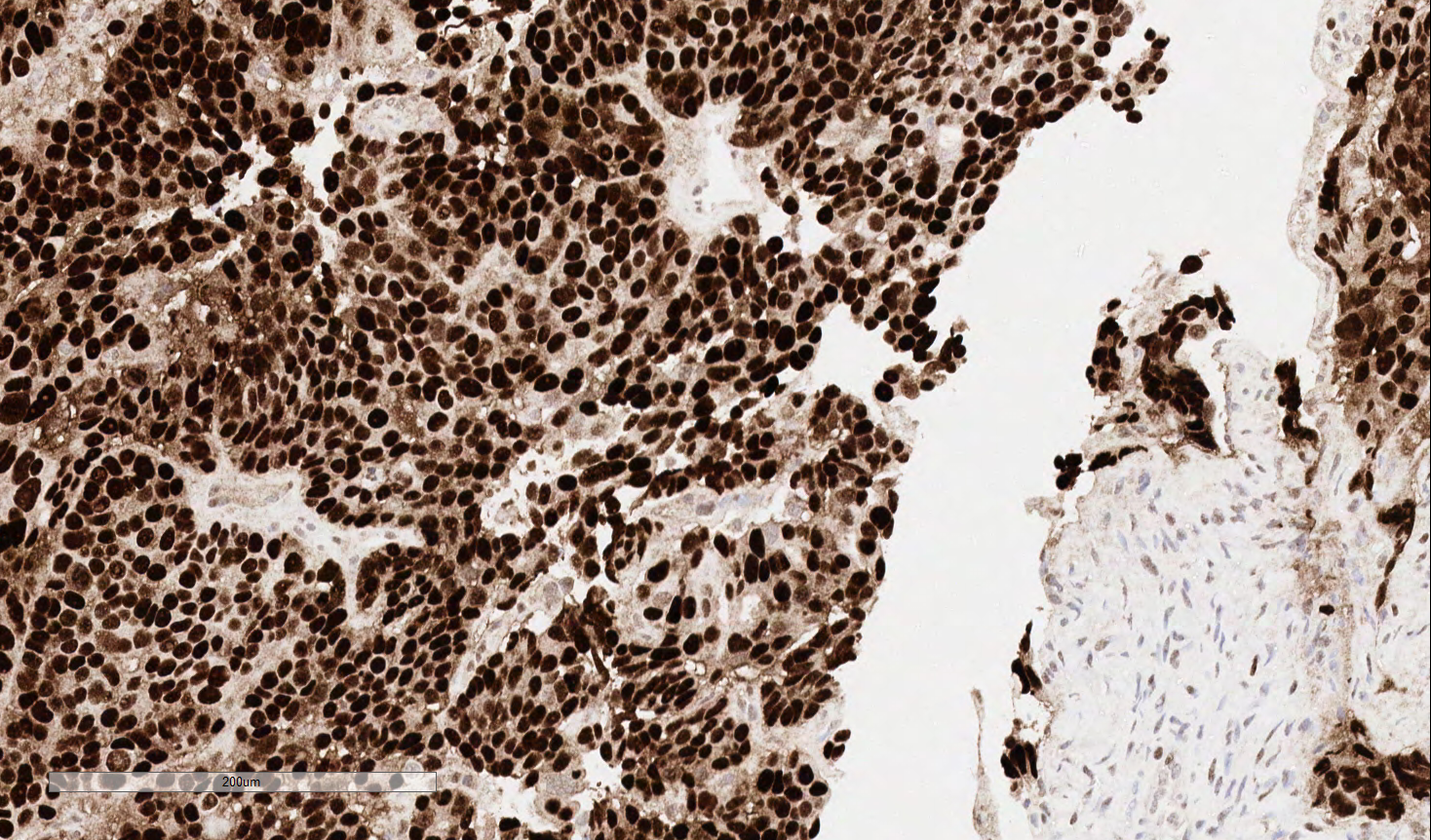

Supplement: Supplementary file 3 — Source Data for Appendix [file EMMM-14-e15729-s005.zip › 831/patient JB348 - p53 zoom.tif]
