# Supplementary material for: Longitudinal monitoring of disease burden and response using ctDNA from dried blood spots in xenograft models
Source: EMBO Mol Med. 2022 Jun 13;14(8):e15729. doi: 10.15252/emmm.202215729 (PMC9358392; doi:10.15252/emmm.202215729)
Supplement: Supplementary file 6 [file EMMM-14-e15729-s002.pdf]

EMBO
Molecular Medicine

Longitudinal monitoring of disease burden and response using ctDNA from dried blood spots in xenograft models

Carolin Sauer, Katrin Heider, Jelena Belic, Samantha Boyle, James Hall, Dominique-Laurent Couturier, Angela An, Aadhitthya Vijayaraghavan, Marika Reinius, Karen Hosking, Maria Vias, Nitzan Rosenfeld, and James Brenton
DOI: 10.15252/emmm.202215729

Corresponding author(s): James Brenton (james.brenton@cruk.cam.ac.uk) , Nitzan Rosenfeld (nitzan.rosenfeld@cruk.cam.ac.uk), Carolin Sauer (carolin.sauer@cruk.cam.ac.uk)

Review Timeline:

Submission Date:	18th Jan 22
Editorial Decision:	23rd Feb 22
Revision Received:	16th Apr 22
Editorial Decision:	9th May 22
Revision Received:	19th May 22
Accepted:	19th May 22

Editor: Jingyi Hou

Transaction Report:

(Note: With the exception of the correction of typographical or spelling errors that could be a source of ambiguity, letters and reports are not edited. Depending on transfer agreements, referee reports obtained elsewhere may or may not be included in this compilation. Referee reports are anonymous unless the Referee chooses to sign their reports.)

1st Editorial Decision 23rd Feb 2022

Thank you again for submitting your work to EMBO Molecular Medicine. We have now heard back from two of the three referees who agreed to evaluate your manuscript. Unfortunately, after a series of reminders, we did not obtain a report from Referee #3. In the interest of time, and since the recommendations of the other two referees are quite similar, I prefer to make a decision now rather than further delay the process. If we receive the comments from Referee #3, we will send them to you, and you can address the issues raised by Referee #3 together with those raised by Referees #1 and #2. As you will see from the reports below, the referees acknowledge the novelty and technical quality of the study. However, they raise a series of concerns, which we would ask you to address in a revision of the manuscript.

I think that the referees' recommendations are relatively straightforward, so there is no need to reiterate their comments. Referee #2 mentioned that the lack of correlation between xTF and tumor volume in the carboplatin-treated arm limits the potential application of the presented tool in PDXs, and they requested additional analyses in this regard. This issue needs to be carefully addressed and discussed in light of the referee's comment.

We would welcome the submission of a revised version within three months for further consideration. Please note that EMBO Molecular Medicine strongly supports a single round of revision. As acceptance or rejection of the manuscript will depend on another round of review, your responses should be as complete as possible.

EMBO Molecular Medicine has a "scooping protection" policy, whereby similar findings that are published by others during review or revision are not a criterion for rejection. Should you decide to submit a revised version, I do ask that you get in touch after three months if you have not completed it to update us on the status.

We are aware that many laboratories cannot function at full efficiency during the current COVID-19/SARS-CoV-2 pandemic and have therefore extended our "scooping protection policy" to cover the period required for a full revision to address the experimental issues. Please let me know should you need additional time, and also if you see a paper with related content published elsewhere.

Please read below for important editorial formatting and consult our author's guidelines for proper formatting of your revised article for EMBO Molecular Medicine.

***** Reviewer's comments *****

Referee #1 (Comments on Novelty/Model System for Author):

Altough this novel approach opens new avenues for experimental studies,there are still challenges and limitations of DBS-based ctDNA sampling in cancer patients. E.g., the use of DBS in cancer patients requires a high concentration of ctDNA in blood to have any analytes available for analysis in this small sample volume.

Referee #1 (Remarks for Author):

This is an interesting study that examines ctDNA monitoring from dried blood spots from PDX models generated from ovarian cancer patients, and draws conclusions regarding the usefulness of DBS and sWGS for quantifying disease burden and modelling therapeutic response in xenografts. Overall, the study has produced some interesting and potentially important results.

Specific comments:

- On line #179, the authors mention alternative methods that also allow sequential blood collection such as submandibular or saphenous bleeding. Have the authors compared the quality of the biological sample between DBS and fresh blood for WGS?
- Have the authors compared the fragment size profile of the PDX DBS to that observed in plasma DNA from fresh blood collection from mice (i.e. submandibular vein)?
- Could the authors address why cardiac puncture instead of tail vein collection was used for the blood spots of the healthy NGS mice controls? Could the authors clarify if they have done duplicates of the healthy controls DBS?
- How and for how long were the DBS stored before WGS analysis?
- Could the authors include a direct comparison between the drug response and the outcome of the patients and their derived PDX?
- It would be interesting to address the challenges and limitations of DBS-based ctDNA sampling on the translation from pre-clinical PDX monitoring to a clinical setting. E.g., the use of DBS in cancer patients requires a high concentration of ctDNA in blood to have any analytes available for analysis in this small sample volume.

Referee #2 (Comments on Novelty/Model System for Author):

The authors present state-of-the art work in which they demonstrate the use of a novel methodology to measure tumor-derived cfDNA fractions in murine models, which is generally limited by the low blood volumes in mice. This work could open perspectives to investigate, in a preclinical setting, multiple research hypotheses related to cfDNA biology and its use for cancer management.
At present, the fact that there is no correlation between xTF and tumour volume in the carboplatin-treated arm, is limiting the applications of this new tool in PDXs. The exact correlation should be further investigated to increase the utility of this model for translation cancer research.

Referee #2 (Remarks for Author):

The authors present very interesting work in which they demonstrate the use of PDX models for ctDNA analysis, based on limited amounts of ctDNA. This work could open perspectives to investigate, in a preclinical setting, multiple research hypotheses related to cfDNA biology and its use for cancer management.
It is most interesting that the initial change in xTF in the presented PDX is predictive of PFS and OS in patients, yet the fact there is no correlation between xTF and tumour volume in the carboplatin-treated arm limits the applications of this new tool in PDXs.
Furthermore, before publication, the following issues should be addressed.

Major remarks

Main text, Line 72, Determination of xTF value: is there a cut-off (in terms of number of reads) below which accurate detection fails?

Main text, Line 84 on false positive background 'Reads from healthy control mice showed <0.1% assignment as human-specific sequences'. It would be interesting to characterize these sequences that are falsely assigned as human (while being murine) - do they overlap with tumour-specific sequences?

Main text, Line 97 on fragment size analyses (Fig 1d). The figure points to difference in fragment sizes in cfDNA from human (tumour) and mice (normal) tissue. This would expected to be the case if the experiment is done after the 3rd generations in the PDX mice. Only then the only human-derived cells are tumour cells and stroma (fibroblasts, immune cells, endothelium) is then of murine origin. The authors however do not specify at which generation the experiments are done and this should be clarified.

Main text, Line 118 on the follow-up of therapy response. Why didn't the authors take a DBS sample shortly after initiation of therapy? This would allow defining more sharply the decline in ctDNA fractions.

Main text, Line 120, '... 18 mice that were treated with carboplatin showed variable decreases in xTF...'. Can the authors further clarify this. What is causing the observed variation? Is it related to the different PDX models (different cancer stages, subtypes)?

Main text, Line 126, '... xTFs increased with increasing tumour volumes and thus disease burden in untreated control mice'. Where increasing ctDNA fractions related to increasing tumour volumes or to increased tumour proliferation/cellular turnover? Do the authors have determined parameters linked to proliferation (Ki-67, mitotic count)?

Further, why was such correlation between increasing xTF and tumour volume not observed in the group of platinum-treated mice? Is there a correlation with cell death or cellular turnover?

Main text, Line 156, '...there was a strong negative correlation between xTF change rate (Fig. 2b) during the first 30 days of treatment and t2 ...'. Can the authors explain why the Mean xTF slope is flat for Pt line 600? According to tumour volume (Fig 3a) this is the model that responds worse, so one would expect an increase of the xTF?

Discussion, Line 165, '...indicates that the initial change in xTF in response to treatment is predictive of PFS and OS'. Is this referring to the patient data? This is not clear. In the PDX setting, there is no 'early' cfDNA sample present (i.e. taken shortly after initiation treatment).

Discussion, Line 185, '...indicating that mechanisms of cfDNA/ctDNA release into the blood stream is similar in 185 mice and humans'. This cannot be concluded from the current dataset, since but non-ctDNA (cfDNA) of human origin was not detected (since not present?).

Discussion, the authors should reflect about the fact that one does not observe a correlation between xTF and tumour volume in the carboplatin-treated arm. This is limiting the applications of his new tool in PDXs and also in patients, e.g. what is happening to a patient that has residual disease after surgery and/or chemo?

Method section, Line 235, the authors refer to the use of heteroscedastic models. Can the authors under build this more statistically, since this is not a commonly used model in PDX experiments.

Method section, Line 258, why did the authors collect blood from control (non-tumour bearing) mice via cardiac puncture, while blood from tumour-bearing mice was taken via the tail. Could this possibly induce any bias in the detection of murine cfDNA specimens?

Minor remarks

Abstract, line 37, 'Use of sequential DBS ctDNA 36 assays will transform future trial designs in both mice and patients'. This should be rephrased in a more prudent way ('could' transform)

Response to Reviewers' comments

Referee #1

Referee #1 (Comments on Novelty/Model System for Author):

Although this novel approach opens new avenues for experimental studies, there are still challenges and limitations of DBS-based ctDNA sampling in cancer patients. E.g., the use of DBS in cancer patients requires a high concentration of ctDNA in blood to have any analytes available for analysis in this small sample volume.

The novelty and strength of our current study is the use of minimal volumes (50µl) of blood to detect, quantify and sequence ctDNA in murine models. This has previously not been possible owing to severe constraints on blood sampling volumes in small rodents. The approaches presented in this manuscript will simplify and improve future preclinical studies in mice, provide important opportunities to investigate research questions related to cf- and ctDNA biology and provides further evidence that DBS is suitable for ctDNA analyses.

In future, we envision DBS-based ctDNA approaches to be translated into the clinic to simplify sample collection, facilitate self-sampling at home and improve patient participation. We agree that there are still limitations and challenges to be overcome regarding the sensitivity of DBS-based ctDNA sampling in cancer patients, and we are currently investigating this further in follow-up studies utilising DBS and small volumes of blood in early and late stage oesophageal cancer and ovarian cancer patients.

Referee #1 (Remarks for Author):

This is an interesting study that examines ctDNA monitoring from dried blood spots from PDX models generated from ovarian cancer patients, and draws conclusions regarding the usefulness of DBS and sWGS for quantifying disease burden and modelling therapeutic response in xenografts. Overall, the study has produced some interesting and potentially important results.

Thank you.

Specific comments:
- On line #179, the authors mention alternative methods that also allow sequential blood collection such as submandibular or saphenous bleeding. Have the authors compared the quality of the biological sample between DBS and fresh blood for WGS?

We have previously compared DBS and plasma obtained from fresh blood samples in our previous study (Heider *et al.*, 2020). The quality of sequencing and size profiles were highly comparable, and resulting copy number profiles from matched plasma and DBS samples strongly correlated ((Heider *et al.*, 2020); Pearson R = 0.97, $P<2.2\times10^{-16}$; Fig. 1C-D). We have not compared different methods of venous access for making DBS—but would not expect differences in ctDNA analysis to arise from the method of blood sampling.

- Have the authors compared the fragment size profile of the PDX DBS to that observed in plasma DNA from fresh blood collection from mice (i.e. submandibular vein)?

Please see response above. In addition, our previous study (Mouliere *et al.*, 2018; Heider *et al.*, 2020) showed PDX fragment size profiles from plasma samples and reported the same peaks around 146 and 166bp for human and mouse specific sequencing reads [(Mouliere *et al.*, 2018) Fig. 2B and D]. Furthermore, both plasma and DBS derived data show the apoptotic ladder with its 10bp periodicity (Chandrananda, Thorne and Bahlo, 2015).

- Could the authors address why cardiac puncture instead of tail vein collection was used for the blood spots of the healthy NGS mice controls? Could the authors clarify if they have done duplicates of the healthy controls DBS?

Owing to project licence limitations we were not permitted to perform blood sampling from non-experimental animals. Cardiac punctures were performed on non-tumour bearing control animals immediately after schedule 1 procedures. We have now added the following information to the methods section:

"Collection of DBS from the tail vein of living non-tumour bearing mice was not possible owing to project licence limitations. Terminal bleeds were performed on non-genetically modified mice that were removed from genotyping experiments via Schedule 1 procedures using syringes pre-flushed with EDTA, and 50µl of collected blood were subsequently spotted onto Whatman FTA™ Classic Cards."

- How and for how long were the DBS stored before WGS analysis?

A previous study showed that DNA can remain stable on filter paper cards for several years (Chaisomchit *et al.*, 2005), which allowed us to collect and store DBS cards for batch processing over several months. In our study, DBS samples were stored for a median of 124 days (IQR 80–168 days) prior to processing for sWGS analyses. This information has now been added to the methods section.

- Could the authors include a direct comparison between the drug response and the outcome of the patients and their derived PDX?

Patients for PDX model generation were selected based on distinct genomic and pathological features (line 115–117; **Fig. EV1**). As highlighted in line 140–143, all four patients were initially platinum-sensitive. It was not meaningful to directly compare clinical treatment response to responses observed in PDX treatment mice because the four patients had differences in important prognostic variables, including disease burden at diagnosis, stage, immediate or delayed surgery, residual disease after surgery and age. An overview of these variables is provided in **Fig. EV1 and EV2**. These clinical variables are not modelled in our experiments (e.g. PDX mice did not undergo surgery as part of their treatment regimen). Treatment response predictions for each of the four patient lines were based on cell autonomous genomic features, such as *BRCA* mutations and HRD signatures for each patient (line 140-143). As highlighted in line 158, observed treatment responses/outcomes in PDX mice confirmed these predictions.

- It would be interesting to address the challenges and limitations of DBS-based ctDNA sampling on the translation from pre-clinical PDX monitoring to a clinical setting. E.g., the use of DBS in cancer patients requires a high concentration of ctDNA in blood to have any analytes available for analysis in this small sample volume.

We agree, and this is the focus of ongoing and future studies in the Rosenfeld and Brenton Laboratories. We are currently investigating the detection and analysis of ctDNA from DBS in early

and late stage oesophageal and ovarian cancer patients. Preliminary data showed that ctDNA was detectable in finger prick DBS in 5/10 of advanced stage oesophageal patients using sWGS. We are exploring further improvements for DBS assay sensitivity by implementing fragmentomic (Mouliere *et al.*, 2018; Cristiano *et al.*, 2019), epigenomic features (Lehmann-Werman *et al.*, 2016) and patient-specific mutations (Abbosh *et al.*, 2017; Parsons *et al.*, 2020; Wan *et al.*, 2020; Zviran *et al.*, 2020; Kurtz *et al.*, 2021). (See Discussion line 209–212).

Referee #2

Referee #2 (Comments on Novelty/Model System for Author):

The authors present state-of-the art work in which they demonstrate the use of a novel methodology to measure tumor-derived cfDNA fractions in murine models, which is generally limited by the low blood volumes in mice. This work could open perspectives to investigate, in a preclinical setting, multiple research hypotheses related to cfDNA biology and its use for cancer management.

At present, the fact that there is no correlation between xTF and tumour volume in the carboplatin-treated arm, is limiting the applications of this new tool in PDXs. The exact correlation should be further investigated to increase the utility of this model for translation cancer research.

Response 2.1 Thank you for highlighting this important point. As discussed in the MS, the major limitation of manual size measurements in PDX experiments are confounding effects related to tissue necrosis, oedema and manual measurement error. Because of these factors, we do not expect to show precise correlation between ctDNA assays and manual tumour size measurements.

The important result, however, is that all samples combined (treated and untreated) show a strong correlation between xTF and manual tumour volume measurement ($R = 0.48$, $p < 0.001$; **Appendix Fig. S6A**). This correlation is also strongly observed in untreated samples ($R = 0.45$, $p < 0.001$), but not in treated samples ($R = 0.056$, $p = 0.78$; **Fig. 2D**). The loss of correlation in treated samples is mostly related to responses in PDX mice from patient line 831 (**Appendix Fig. S6B**). As illustrated in **Fig. 2B** (right panel) there is a steep decrease in xTF (i.e. ctDNA concentration) in treated mice from line 831 during the first 30 days of treatment. In contrast, tumour volume measurements from the same mice plateau during the first 41 days of treatment and no decrease is observed (**Fig. 3D**). Importantly, it is the change in xTF values, and not tumour volumes, during the first 30 days of treatment that strongly correlate with overall treatment outcome (**Fig. 3E**). Future work will be required to further understand these dynamics (which are likely to be patient-specific representing differences in intrinsic sensitivity and clonal expansion) and study the involvement of necrosis and tumour proliferation/cellular turnover, on both tumour volume and ctDNA shedding. Our data therefore supports the assertion that ctDNA measures (i.e. xTF values) offer a more accurate readout of initial treatment response as less prone to confounding factors on manual size measurements as discussed above that can bias tumour volume estimates. Please also refer to **Response 2.2** and **2.3. A precis of this response is now included in the Discussion.**

Referee #2 (Remarks for Author):

The authors present very interesting work in which they demonstrate the use of PDX models for ctDNA analysis, based on limited amounts of ctDNA. This work could open perspectives to

investigate, in a preclinical setting, multiple research hypotheses related to cfDNA biology and its use for cancer management.

It is most interesting that the initial change in xTF in the presented PDX is predictive of PFS and OS in patients, yet the fact there is no correlation between xTF and tumour volume in the carboplatin-treated arm limits the applications of this new tool in PDXs.

Please see **Response 2.1** above.

Furthermore, before publication, the following issues should be addressed.

Major remarks

Main text, Line 72, Determination of xTF value: is there a cut-off (in terms of number of reads) below which accurate detection fails?

The distribution of sequencing reads following Xenomapper alignment and removal of duplicate reads is illustrated in the figure below for total (purple), mouse-specific (blue) and human-specific (pink) reads across all samples. The median number of total reads was 3,037,751 (IQR 771,738–6,847,240). Importantly, human-specific reads were detected in all PDX DBS samples, resulting in xTFs values > xTFs detected in non-tumour bearing mice. As such, we have not yet observed a read-count threshold below which accurate detection failed.

We did perform in silico dilution experiments (line 88–93; **Fig. 1C**) combining sequencing reads from healthy mouse DBS and DBS collected from five cancer patients. Mouse and human fastq files were downsampled and merged to generate a seven-point dilution series with a total read count of $6.5{\times}10^6$ reads. As discussed in line 90, we were able to accurately detect human reads for all seven dilution points. The lowest tested dilution point represented an xTF of 0.01, which is equivalent to 65,000 human reads.

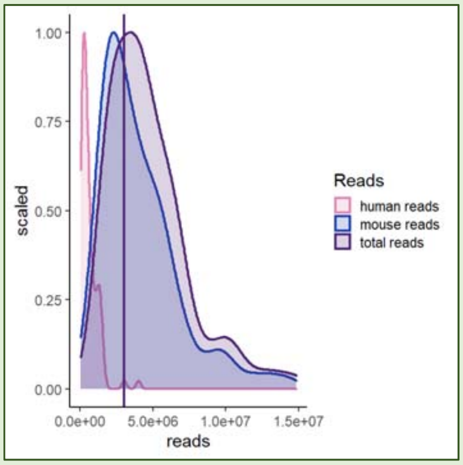

Number of sequencing reads density plot for total reads (purple), mouse-specific reads (blue) and human-specific reads (pink) from PDX DBS samples. The purple vertical line indicates the median of the number of total sequencing reads.

Main text, Line 84 on false positive background 'Reads from healthy control mice showed <0.1% assignment as human-specific sequences'. It would be interesting to characterize these sequences that are falsely assigned as human (while being murine) - do they overlap with tumour-specific sequences?

We agree with the reviewer's comment that it would be interesting to further understand why some mouse sequencing reads (<0.1%) from non-tumour bearing mice incorrectly align with the human genome, when using Xenomapper (Wakefield, 2016) following the authors' guidelines and instructions (https://github.com/genomematt/xenomapper). Importantly, sequencing reads were aligned to the mouse (mm10) and human (hg19) reference genomes (line 321–327). Samples were not aligned to tumour-specific sequences from matched patient tumour tissues.

To address this question further, we analysed fragment size profiles of mouse-specific and (falsely identified) human-specific reads obtained from DBS samples from healthy non-tumour bearing mice (**Figure below**). In comparison to human reads obtained from PDX DBS samples (**Fig. 1D**), falsely identified human reads from healthy mice did not display the characteristic fragment size pattern for ctDNA (Underhill *et al.*, 2016; Mouliere *et al.*, 2018; Heider *et al.*, 2020). Instead, human read fragments were significantly smaller (peak <100bp) with the majority of reads showing fragment sizes of <50bp (note that fragments <30bp fragment length were excluded from all analyses as they had increased noise and ambiguous alignments).
In contrast, mouse reads from both sample types (PDX DBS and healthy mouse DBS) showed the characteristic fragment size distribution for cfDNA (Underhill *et al.*, 2016; Mouliere *et al.*, 2018; Heider *et al.*, 2020). Together, this suggests that the small fraction of falsely identified human reads in healthy/non-tumour bearing mice are predominantly caused by small DNA fragments resulting in ambiguous species-specific alignment. This has now been added to the text (line 98) and as **Appendix Figure S1** to the main manuscript.

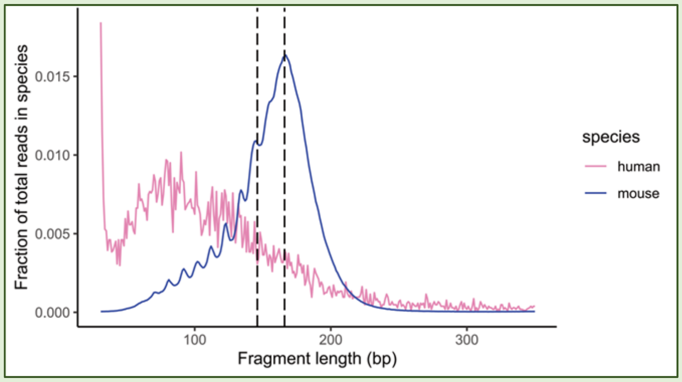

Fragment length distributions of human- (pink) and mouse- (blue) specific reads from a DBS sample obtained from a healthy non-tumour bearing mouse. Two vertical lines indicate 146 and 166 bp, the observed peaks for ctDNA and cfDNA, respectively.

Main text, Line 97 on fragment size analyses (Fig 1d). The figure points to difference in fragment sizes in cfDNA from human (tumour) and mice (normal) tissue. This would expected to be the case if the experiment is done after the 3rd generations in the PDX mice. Only then the only human-derived cells are tumour cells and stroma (fibroblasts, immune cells, endothelium) is then of murine origin. The authors however do not specify at which generation the experiments are done and this should be clarified.

Thank you for pointing this out. This information has now been added to the methods section. Indeed, all treatment experiments were carried out in 3rd and 4th generation PDX mice. This information has been added to the methods and included in the discussion.

Main text, Line 118 on the follow-up of therapy response. Why didn't the authors take a DBS sample shortly after initiation of therapy? This would allow defining more sharply the decline in ctDNA fractions.

We agree that this would indeed have been an interesting timepoint to look at. Currently, little is known about ctDNA kinetics during the first 24–48 hours post treatment. The methods presented in this manuscript will certainly allow more focussed experiments to explore this further. However, in this study, the number of samples and their timepoints were limited by project license and home office regulations, which permitted "blood collection via superficial vein (optional and on more than one occasion) at a volume of 10% of Total Blood Volume (i.e. for a 20g mouse 0.105mL) on any one occasion and no more than 15% within 28 days" (i.e. for a 20g mouse 0.157mL, which equals three DBS at three timepoints).

Main text, Line 120, '... 18 mice that were treated with carboplatin showed variable decreases in xTF...'. Can the authors further clarify this? What is causing the observed variation? Is it related to the different PDX models (different cancer stages, subtypes)?

Variable decreases in xTFs were observed across the four different PDX lines (from the four patients). We have now clarified this in the text. All PDX models were derived from HGSOC tumours and were of the same subtypes. An overview of disease stages and other clinical variables is given in **Fig. EV1 and EV2**. As discussed in line 140–145 and the following paragraph, the variation in treatment response between the different PDX lines is most likely caused by differences in intrinsic sensitivity to carboplatin therapy from *BRCA* mutation status and HRD signatures.

Main text, Line 126, '... xTFs increased with increasing tumour volumes and thus disease burden in untreated control mice'. Where increasing ctDNA fractions related to increasing tumour volumes or to increased tumour proliferation/cellular turnover? Do the authors have determined parameters linked to proliferation (Ki-67, mitotic count)?

Response 2.2 We were not able to look at cellular parameters since our study was focussed on non-invasive approaches to monitor ctDNA and treatment response over several timepoints. This prohibited the simultaneous collection of tumour material which would have required major surgery or culling of the animal. We are currently working on optimising fine needle aspiration approaches to collect biopsies at the same time as DBS samples. Together with the methods presented in this manuscript, this will allow focussed studies into understanding the biology of cfDNA and factors that influence DNA shedding into the blood in untreated and treated conditions.

Further, why was such correlation between increasing xTF and tumour volume not observed in the group of platinum-treated mice? Is there a correlation with cell death or cellular turnover?

Response 2.3 As outlined above, investigations of parameters affecting DNA shedding into the blood was out of scope for the current study. Our methodologies and approaches presented in this manuscript, however, will provide an important platform for future research into cfDNA biology and the factors that influence cfDNA/ctDNA release into the blood in the presence and absence of treatment. Please also see **Response 2.1** and **2.2**.

Main text, Line 156, '...there was a strong negative correlation between xTF change rate (Fig. 2b) during the first 30 days of treatment and t2 ...'. Can the authors explain why the Mean xTF slope is flat for Pt line 600? According to tumour volume (Fig 3a) this is the model that responds worse, so one would expect an increase of the xTF?

While this model (patient line 600) was the worst responder, this does not mean that there was no response at all (see **Fig. 2** and **3**). Treatment response was defined based on multiple parameters, including the duration of treatment response (t_2-t_1) and the time until tumour regrowth (t_2), both of which were the shortest in PDXs from patient line 600. As shown in **Fig. 3a**, there was an initial response in tumour volume, with regrowth starting around day 23. Similarly, there was an initial decrease in xTF for PDX mice (day 16) from patient line 600. xTF values increased again by day 29 (the final DBS sample collection timepoint). The mean xTF slope was calculated as the average rate of change (across all treatment mice within each patient line) between day 1 (baseline) and day 29. For patient line 600, this rate of change (slope) resulted in a flat line. As shown in **Fig. 3e**, these rate of change estimates (Mean xTF slope, as shown on the y-axis) was strongly anti-correlated with the time to regrowth (t_2) and the time to endpoint. For example, patient line 828 showed the best response to treatment, had the steepest xTF change rate (slope) and showed the longest time to regrowth and endpoint.

Discussion, Line 165, '...indicates that the initial change in xTF in response to treatment is predictive of PFS and OS'. Is this referring to the patient data? This is not clear. In the PDX setting, there is no 'early' cfDNA sample present (i.e. taken shortly after initiation treatment).

Thank you for pointing this out, we have now clarified this in the text. The PFS and OS here are referring to PDX mice, as estimated by the "time until tumour regrowth" (PFS; t_2; see line 156) and "time until endpoint" (OS). Please also refer to **Fig. 3e**.

Discussion, Line 185, '...indicating that mechanisms of cfDNA/ctDNA release into the blood stream is similar in mice and humans'. This cannot be concluded from the current dataset, since but non-ctDNA (cfDNA) of human origin was not detected (since not present?).

This conclusion was based on previous knowledge of size profiles of human ctDNA and cfDNA. The murine cfDNA detected in our study shows the same size profile as previously reported for human cfDNA, while the human ctDNA detected in our PDX mice showed the same size profile as previously reported for human ctDNA from cancer patients (Underhill *et al.*, 2016; Mouliere *et al.*, 2018; Heider *et al.*, 2020). We have now improved the wording and added the reference to the main text of the manuscript.

Discussion, the authors should reflect about the fact that one does not observe a correlation between xTF and tumour volume in the carboplatin-treated arm. This is limiting the applications of his new tool in PDXs and also in patients, e.g. what is happening to a patient that has residual disease after surgery and/or chemo?

Please see **Response 2.1**.

Method section, Line 235, the authors refer to the use of heteroscedastic models. Can the authors under build this more statistically, since this is not a commonly used model in PDX experiments?

We agree with the reviewer that heteroscedastic models are less commonly used than models that assume equality of variance (i.e. homoscedastic fits). However, we note that heteroscedastic growth mixed models are taught (see, for example, (Pinheiro and Bates, 2000) Sections 4.3.1 and 5.2 and

(Bliese and Ployhart, 2002)) and used in practice (in the modelling, for example, of tree (Aguirre-Calderón, 2018), bacteria population (Cao, Francisco-Fernández and Quinto, 2010) and tumour growth (Ros *et al.*, 2020)). More importantly, the danger of under-specifying the model by preferring a homoscedastic fit when errors are heteroscedastic is well documented in the specific setting of growth linear mixed models (Fu, Huang and Ou, 2015), with the authors stating that this issue is 'critical in biomedical research'.

The presence of heteroscedasticity in (growth) linear mixed models is typically assessed through model checks visually comparing the variance of the residuals between conditions. More formal ways consider likelihood ratio tests comparing the likelihood of fits with and without modelling the variance of the error terms ((Pinheiro and Bates, 2000), page 224) or Bartlett tests of homogeneity of variances of the residuals. If no heteroscedasticity is present, p-values of these tests follow a uniform distribution which could be assessed with a Kolmogorov-Smirnov test. For the 4 patients considered in our study, the Bartlett test p-values were 0.2605 (patient 600), 0.01324 (patient 771), 0.3923 (patient 828) and 0.1496 (patient 831) leading to a Kolmogorov-Smirnov test p-value of 0.061, suggesting, together with our model check assessment, that the residual variance may indeed be a function of treatment group. Therefore, to be on the safe side, we preferred heteroscedastic fits.

Method section, Line 258, why did the authors collect blood from control (non-tumour bearing) mice via cardiac puncture, while blood from tumour-bearing mice was taken via the tail. Could this possibly induce any bias in the detection of murine cfDNA specimens?

Owing to project licence limitations we were not permitted to perform blood sampling from non-experimental animals. Cardiac punctures were performed on non-tumour bearing control animals immediately after schedule 1 procedures. We added the following information to the methods section:

"Collection of DBS from the tail vein of living non-tumour bearing mice was not possible owing to project licence limitations prohibiting scientific procedures on non-specified/study-registered animals. Terminal bleeds were performed on non-genetically modified mice that were removed from genotyping experiments via Schedule 1 procedures using syringes pre-flushed with EDTA, and 50µl of collected blood were subsequently spotted onto Whatman FTA™ Classic Cards."

The non-tumour bearing control mice in this study were used to test and confirm the specificity of our approach by showing that no (<0.1%) human-specific cfDNA was detected in non-tumour bearing control mice. We have no reason to believe that the sampling site of blood could affect the quantity or quality of cfDNA in untreated, healthy mice. In addition, a recent study on experimental post-operative sepsis in porcine models has shown that there was no difference in cfDNA concentration collected from the artery, hepatic vein, portal vein and jugular bulb (see Fig. 2) in untreated pigs (Nyberg *et al.*, 2020).

Minor remarks

Abstract, line 37, 'Use of sequential DBS ctDNA 36 assays will transform future trial designs in both mice and patients'. This should be rephrased in a more prudent way ('could' transform)

We agree, and have changed the wording accordingly.

References

Abbosh, C. *et al.* (2017) 'Phylogenetic ctDNA analysis depicts early-stage lung cancer evolution', *Nature*, 545(7655), pp. 446–451. doi: 10.1038/nature22364.

Aguirre-Calderón (2018) 'How to correct the heteroscedasticity and autocorrelation of residuals in taper and height growth models?', *Revista Mexicana de Ciencias Forestales*, 9(49), pp. 1–31.

Bliese, P. D. and Ployhart, R. E. (2002) 'Growth modeling using random coefficient models: Model building, testing, and illustrations', *Organizational Research Methods*, 5, pp. 362–387.

Cao, R., Francisco-Fernández, M. and Quinto, E. J. (2010) 'A random effect multiplicative heteroscedastic model for bacterial growth', *BMC Bioinformatics*, 11(77), pp. 1–12.

Chaisomchit, S. *et al.* (2005) 'Stability of genomic DNA in dried blood spots stored on filter paper', *Southeast Asian Journal of Tropical Medicine and Public Health*, 36(1), pp. 270–273.

Chandrananda, D., Thorne, N. P. and Bahlo, M. (2015) 'High-resolution characterization of sequence signatures due to non-random cleavage of cell-free DNA', *BMC Medical Genomics*, 8(1), pp. 1–19. doi: 10.1186/s12920-015-0107-z.

Cristiano, S. *et al.* (2019) 'Genome-wide cell-free DNA fragmentation in patients with cancer', *Nature*, 570(7761), pp. 385–389. doi: 10.1038/s41586-019-1272-6.

Fu, C. Y., Huang, H.-Y. and Ou, Y.-H. (2015) 'The effects of missing serial effects and/or heteroscedastic errors on mixed models using repeated growth data', *Journal of Statistical Computation and Simulation*, 85(16), pp. 3367–3382.

Heider, K. *et al.* (2020) 'Detection of ctDNA from Dried Blood Spots after DNA Size Selection', *Clinical Chemistry*, 66(5), pp. 697–705. doi: 10.1093/clinchem/hvaa050.

Kurtz, D. M. *et al.* (2021) 'Enhanced detection of minimal residual disease by targeted sequencing of phased variants in circulating tumor DNA', *Nature Biotechnology*. doi: 10.1038/s41587-021-00981-w.

Lehmann-Werman, R. *et al.* (2016) 'Identification of tissue-specific cell death using methylation patterns of circulating DNA', *Proceedings of the National Academy of Sciences of the United States of America*, 113(13), pp. E1826–E1834. doi: 10.1073/pnas.1519286113.

Mouliere, F. *et al.* (2018) 'Enhanced detection of circulating tumor DNA by fragment size analysis', *Science Translational Medicine*, 10(466), pp. 1–14. doi: 10.1126/scitranslmed.aat4921.

Nyberg, A. *et al.* (2020) 'Lung-protective ventilation suppresses systemic and hepatic vein levels of cell-free DNA in porcine experimental post-operative sepsis', *BMC Pulmonary Medicine*, 20(1), pp. 1–12. doi: 10.1186/s12890-020-01239-y.

Parsons, H. A. *et al.* (2020) 'Sensitive Detection of Minimal Residual Disease in Patients Treated for Early-Stage Breast Cancer', *Clinical Cancer Research*, 26(11), pp. 2556–2564. doi: 10.1158/1078-0432.CCR-19-3005.

Pinheiro and Bates (2000) *Mixed-Effects Models in S and S-PLUS*. Springer. Available at: https://link.springer.com/book/10.1007/b98882.

Ros, S. *et al.* (2020) 'Metabolic Imaging Detects Resistance to PI3Kα Inhibition Mediated by Persistent FOXM1 Expression in ER+ Breast Cancer', *Cancer Cell*, 38(4), pp. 516–533.

Underhill, H. R. *et al.* (2016) 'Fragment Length of Circulating Tumor DNA', *PLoS Genetics*, 12(7), pp. 1–24. doi: 10.1371/journal.pgen.1006162.

Wakefield, M. J. (2016) 'Xenomapper: Mapping reads in a mixed species context', *The Journal of*

Open Source Software, 1(1), p. 18. doi: 10.21105/joss.00018.

Wan, J. C. M. *et al.* (2020) 'ctDNA monitoring using patient-specific sequencing and integration of variant reads', *Science Translational Medicine*, 12(548), pp. 1–17. doi: 10.1126/scitranslmed.aaz8084.

Zviran, A. *et al.* (2020) 'Genome-wide cell-free DNA mutational integration enables ultra-sensitive cancer monitoring', *Nature Medicine*, 26(7), pp. 1114–1124. doi: 10.1038/s41591-020-0915-3.

Thank you for the submission of your revised manuscript to EMBO Molecular Medicine. We have now received the enclosed report from the two referees who agreed to re-assess it. As you will see, the referees are now overall supportive, and I am pleased to inform you that we will be able to accept your manuscript pending the following amendments:

1. Please address the remaining concerns from Referee #2.

On a more editorial level.

***** Reviewer's comments *****

Referee #1 (Comments on Novelty/Model System for Author):

Please see my original comment. This is a revised version.

Referee #1 (Remarks for Author):

The authors have addressed my comments in a satisfactory way and the quality of this interesting manuscript has improved. I have no further comments.

Referee #2 (Remarks for Author):

The authors addressed the majority of our comments well and now present a nice piece of work which may serve the scientific community. Given our lack of expertise in the statistics model applied in the tumour growth curve modelling, we cannot correctly judged the soundness of the tumour growth data. In the initial review process we asked the authors to elaborate on this method, yet this was not addressed.

Some textual remarks should be addressed before publication.

*Results, line 125, the authors added the word 'patient-specific', yet this is misleading as it might give the impression that the xTF observations in the PDX models concur with corresponding measurements in samples from the patients from which the PDX models were derived.

*Results, line 132-135, "This correlation was also strongly observed in untreated mice. [...], mostly related to responses in PDX mice from patient line 831". These lines are not clearly formulated. The authors should rephrase, stating more explicitly that a direct correlation between xTF and tumour volumes was observed in all untreated mice, but not in all treated mice.

Response to Reviewers' comments – round 2

Referee #1

Referee #1 (Comments on Novelty/Model System for Author):

Please see my original comment. This is a revised version.

Referee #1 (Remarks for Author):

The authors have addressed my comments in a satisfactory way and the quality of this interesting manuscript has improved. I have no further comments.

Thank you!

Referee #2

Referee #2 (Remarks for Author):

The authors addressed the majority of our comments well and now present a nice piece of work which may serve the scientific community. Given our lack of expertise in the statistics model applied in the tumour growth curve modelling, we cannot correctly judged the soundness of the tumour growth data. In the initial review process we asked the authors to elaborate on this method, yet this was not addressed.

Please refer to our previous **Response 2.**4, in which we provide detailed scientific justification for the statistical approach used to model tumour growth.

Some textual remarks should be addressed before publication.

*Results, line 125, the authors added the word 'patient-specific', yet this is misleading as it might give the impression that the xTF observations in the PDX models concur with corresponding measurements in samples from the patients from which the PDX models were derived.

We have now addressed this in the main manuscript text.

*Results, line 132-135, "This correlation was also strongly observed in untreated mice. [...], mostly related to responses in PDX mice from patient line 831". These lines are not clearly formulated. The authors should rephrase, stating more explicitly that a direct correlation between xTF and tumour volumes was observed in all untreated mice, but not in all treated mice.

We have now addressed this in the main manuscript text.

Accepted **19th May 2022**

We are pleased to inform you that your manuscript is accepted for publication and is now being sent to our publisher to be included in the next available issue of EMBO Molecular Medicine.

EMBO Press Author Checklist

Corresponding Author Name: Carolin M. Sauer, Nitzan Rosenfeld, James D. Brenton	
Journal Submitted to: EMBO Molecular Medicine	
Manuscript Number: EMM-2022-15729	

USEFUL LINKS FOR COMPLETING THIS FORM
The EMBO Journal - Author Guidelines
EMBO Reports - Author Guidelines
Molecular Systems Biology - Author Guidelines
EMBO Molecular Medicine - Author Guidelines

Reporting Checklist for Life Science Articles (updated January 2022)
This checklist is adapted from Materials Design Analysis Reporting (MDAR) Checklist for Authors. MDAR establishes a minimum set of requirements in transparent reporting in the life sciences (see Statement of Task: 10.31222/osf.io/9sm4x). Please follow the journal's guidelines in preparing your manuscript.
Please note that a copy of this checklist will be published alongside your article.

Abridged guidelines for figures
1. Data
The data shown in figures should satisfy the following conditions:
- the data were obtained and processed according to the field's best practice and are presented to reflect the results of the experiments in an accurate and unbiased manner.
- ideally, figure panels should include only measurements that are directly comparable to each other and obtained with the same assay.
- plots include clearly labeled error bars for independent experiments and sample sizes. Unless justified, error bars should not be shown for technical replicates.
- if n<5, the individual data points from each experiment should be plotted. Any statistical test employed should be justified.
- Source Data should be included to report the data underlying figures according to the guidelines set out in the authorship guidelines on Data Presentation.

2. Captions
Each figure caption should contain the following information, for each panel where they are relevant:
- a specification of the experimental system investigated (eg cell line, species name).
- the assay(s) and method(s) used to carry out the reported observations and measurements.
- an explicit mention of the biological and chemical entity(ies) that are being measured.
- an explicit mention of the biological and chemical entity(ies) that are altered/varied/perturbed in a controlled manner.
- the exact sample size (n) for each experimental group/condition, given as a number, not a range;
- a description of the sample collection allowing the reader to understand whether the samples represent technical or biological replicates (including how many animals, litters, cultures, etc.).
- a statement of how many times the experiment shown was independently replicated in the laboratory.
- definitions of statistical methods and measures:
  - common tests, such as t-test (please specify whether paired vs. unpaired), simple χ2 tests, Wilcoxon and Mann-Whitney tests, can be unambiguously identified by name only, but more complex techniques should be described in the methods section;
  - are tests one-sided or two-sided?
  - are there adjustments for multiple comparisons?
  - exact statistical test results, e.g., P values = x but not P values < x;
  - definition of 'center values' as median or average;
  - definition of error bars as s.d. or s.e.m.

Please complete ALL of the questions below.
Select "Not Applicable" only when the requested information is not relevant for your study.

Materials

Newly Created Materials	Information included in the manuscript?	In which section is the information available? (Reagents and Tools Table, Materials and Methods, Figures, Data Availability Section)
New materials and reagents need to be available; do any restrictions apply?	Not Applicable	

Antibodies	Information included in the manuscript?	In which section is the information available? (Reagents and Tools Table, Materials and Methods, Figures, Data Availability Section)
For **antibodies** provide the following information:<br>- Commercial antibodies: RRID (if possible) or supplier name, catalogue number and or/clone number<br>- Non-commercial: RRID or citation	Yes	Materials and Methods

DNA and RNA sequences	Information included in the manuscript?	In which section is the information available? (Reagents and Tools Table, Materials and Methods, Figures, Data Availability Section)
**Short novel DNA or RNA including primers, probes**: provide the sequences.	Not Applicable	

Cell materials	Information included in the manuscript?	In which section is the information available? (Reagents and Tools Table, Materials and Methods, Figures, Data Availability Section)
**Cell lines:** Provide species information, strain. Provide accession number in repository **OR** supplier name, catalog number, clone number, and/**OR** RRID.	Not Applicable	
**Primary cultures:** Provide species, strain, sex of origin, genetic modification status.	Not Applicable	
Report if the cell lines were recently **authenticated** (e.g., by STR profiling) and tested for mycoplasma contamination.	Not Applicable	

Experimental animals	Information included in the manuscript?	In which section is the information available? (Reagents and Tools Table, Materials and Methods, Figures, Data Availability Section)
**Laboratory animals or Model organisms:** Provide species, strain, sex, age, genetic modification status. Provide accession number in repository **OR** supplier name, catalog number, clone number, **OR** RRID.	Yes	Materials and Methods
**Animal observed in or captured from the field:** Provide species, sex, and age where possible.	Not Applicable	
Please detail **housing and husbandry conditions**.	Yes	Materials and Methods

Plants and microbes	Information included in the manuscript?	In which section is the information available? (Reagents and Tools Table, Materials and Methods, Figures, Data Availability Section)
**Plants:** provide species and strain, ecotype and cultivar where relevant, unique accession number if available, and source (including location for collected wild specimens).	Not Applicable	
**Microbes:** provide species and strain, unique accession number if available, and source.	Not Applicable	

Human research participants	Information included in the manuscript?	In which section is the information available? (Reagents and Tools Table, Materials and Methods, Figures, Data Availability Section)
If collected and within the bounds of privacy constraints report on age, sex and gender or ethnicity for all study participants.	Yes	Materials and Methods; EV Fig. 2

Core facilities	Information included in the manuscript?	In which section is the information available? (Reagents and Tools Table, Materials and Methods, Figures, Data Availability Section)
If your work benefited from core facilities, was their service mentioned in the acknowledgments section?	Yes	Acknowledgments section

Design

Study protocol	Information included in the manuscript?	In which section is the information available? (Reagents and Tools Table, Materials and Methods, Figures, Data Availability Section)
If study protocol has been **pre-registered, provide DOI in the manuscript**. For clinical trials, provide the trial registration number **OR** cite DOI.	Not Applicable	
Report the **clinical trial registration number** (at ClinicalTrials.gov or equivalent), where applicable.	Not Applicable	

Laboratory protocol	Information included in the manuscript?	In which section is the information available? (Reagents and Tools Table, Materials and Methods, Figures, Data Availability Section)
Provide DOI OR other citation details if **external detailed step-by-step protocols** are available.	Not Applicable	

Experimental study design and statistics	Information included in the manuscript?	In which section is the information available? (Reagents and Tools Table, Materials and Methods, Figures, Data Availability Section)
Include a statement about **sample size** estimate even if no statistical methods were used.	Yes	Main text; Materials and Methods; Figure legends
Were any steps taken to minimize the effects of subjective bias when allocating animals/samples to treatment (e.g. **randomization procedure**)? If yes, have they been described?	Not Applicable	
Include a statement about **blinding** even if no blinding was done.	Not Applicable	
Describe **inclusion/exclusion criteria** if samples or animals were excluded from the analysis. Were the criteria pre-established?<br><br>If sample or data points were omitted from analysis, report if this was due to attrition or intentional exclusion and provide justification.	Not Applicable	
For every figure, are **statistical tests** justified as appropriate? Do the data meet the assumptions of the tests (e.g., normal distribution)? Describe any methods used to assess it. Is there an estimate of variation within each group of data? Is the variance similar between the groups that are being statistically compared?	Yes	Materials and Methods; Figure legends

Sample definition and in-laboratory replication	Information included in the manuscript?	In which section is the information available? (Reagents and Tools Table, Materials and Methods, Figures, Data Availability Section)
In the figure legends: state number of times the experiment was **replicated** in laboratory.	Yes	Figure legends
In the figure legends: define whether data describe **technical or biological replicates**.	Yes	Figure legends

Ethics

Ethics	Information included in the manuscript?	In which section is the information available? (Reagents and Tools Table, Materials and Methods, Figures, Data Availability Section)
Studies involving **human participants**: State details of **authority granting ethics approval** (IRB or equivalent committee(s), provide reference number for approval.	Yes	Materials and Methods (08/H0306/61)
Studies involving **human participants**: Include a statement confirming that **informed consent** was obtained from all subjects and that the experiments conformed to the principles set out in the WMA Declaration of Helsinki and the Department of Health and Human Services Belmont Report.	Yes	Materials and Methods
Studies involving **human participants:** For publication of **patient photos**, include a statement confirming that consent to publish was obtained.	Not Applicable	
Studies involving experimental **animals**: State details of **authority granting ethics approva**l (IRB or equivalent committee(s), provide reference number for approval. Include a statement of compliance with ethical regulations.	Yes	Materials and Methods
Studies involving **specimen and field samples:** State if relevant **permits** obtained, provide details of authority approving study; if none were required, explain why.	Not Applicable	

Dual Use Research of Concern (DURC)	Information included in the manuscript?	In which section is the information available? (Reagents and Tools Table, Materials and Methods, Figures, Data Availability Section)
Could your study fall under dual use research restrictions? Please check biosecurity documents and list of **select agents and toxins** (CDC): https://www.selectagents.gov/sat/list.htm	Not Applicable	
If you used a select agent, is the security level of the lab appropriate and reported in the manuscript?	Not Applicable	
If a study is subject to dual use research of concern regulations, is the name of the **authority granting approval and reference number** for the regulatory approval provided in the manuscript?	Not Applicable	

Reporting

The MDAR framework recommends adoption of discipline-specific guidelines, established and endorsed through community initiatives. Journals have their own policy about requiring specific guidelines and recommendations to complement MDAR.

Adherence to community standards	Information included in the manuscript?	In which section is the information available? (Reagents and Tools Table, Materials and Methods, Figures, Data Availability Section)
State if relevant guidelines or checklists (e.g., **ICMJE, MIBBI, ARRIVE, PRISMA**) have been followed or provided.	Not Applicable	
For **tumor marker prognostic studies,** we recommend that you follow the **REMARK** reporting guidelines (see link list at top right). See author guidelines, under 'Reporting Guidelines'. Please confirm you have followed these guidelines.	Not Applicable	
For **phase II and III randomized controlled trials**, please refer to the **CONSORT** flow diagram (see link list at top right) and submit the CONSORT checklist (see link list at top right) with your submission. See author guidelines, under 'Reporting Guidelines'. Please confirm you have submitted this list.	Not Applicable	

Data Availability

Data availability	Information included in the manuscript?	In which section is the information available? (Reagents and Tools Table, Materials and Methods, Figures, Data Availability Section)
Have **primary datasets** been deposited according to the journal's guidelines (see 'Data Deposition' section) and the respective accession numbers provided in the Data Availability Section?	Yes	Materials and Methods; Data Availability Section
Were **human clinical and genomic datasets** deposited in a public access-controlled repository in accordance to ethical obligations to the patients and to the applicable consent agreement?	Yes	Materials and Methods; Data Availability Section
Are **computational models** that are central and integral to a study available without restrictions in a machine-readable form? Were the relevant accession numbers or links provided?	Yes	Materials and Methods
If publicly available data were reused, provide the respective **data citations in the reference list**.	Not Applicable	
